# Sex-Specific Development in Haplodiploid Honeybee Is Controlled by the Female-Embryo-Specific Activation of Thousands of Intronic LncRNAs

**DOI:** 10.3389/fcell.2021.690167

**Published:** 2021-08-06

**Authors:** Miao Wang, Dong Chen, Huoqing Zheng, Liuwei Zhao, Xiaofeng Xue, Fengyun Yu, Yu Zhang, Chao Cheng, Qingsheng Niu, Shuai Wang, Yi Zhang, Liming Wu

**Affiliations:** ^1^Institute of Apicultural Research, Chinese Academy of Agricultural Sciences, Beijing, China; ^2^ABLife BioBigData Institute, Wuhan, China; ^3^Laboratory for Genome Regulation and Human Health, ABLife Inc., Wuhan, China; ^4^College of Animal Science, Zhejiang University, Hangzhou, China; ^5^Department of Scientific Research, Jilin Province Institute of Apicultural Science, Jilin, China

**Keywords:** embryonic development, haplodiploid honeybee, long non-coding RNAs, sex determination, zygotic genome activation

## Abstract

Embryonic development depends on a highly coordinated shift in transcription programs known as the maternal-to-zygotic transition (MZT). It remains unclear how haploid and diploid embryo coordinate their genomic activation and embryonic development during MZT in haplodiploid animals. Here, we applied a single-embryo RNA-seq approach to characterize the embryonic transcriptome dynamics in haploid males vs. diploid females of the haplodiploid insect honeybee (*Apis mellifera*). We observed typical zygotic genome activation (ZGA) occurred in three major waves specifically in female honeybee embryos; haploid genome activation was much weaker and occurred later. Strikingly, we also observed three waves of transcriptional activation for thousands of long non-coding transcripts (lncRNA), 73% of which are transcribed from intronic regions and 65% were specific to female honeybee embryos. These findings support a model in which introns encode thousands of lncRNAs that are expressed in a diploid-embryo-specific and ZGA-triggered manner that may have potential functions to regulate gene expression during early embryonic development in the haplodiploid insect honeybee.

## Graphical Abstract

Current working model of the three waves of ZGA in diploid embryos, and the slow maternal degradation in haploid embryos.

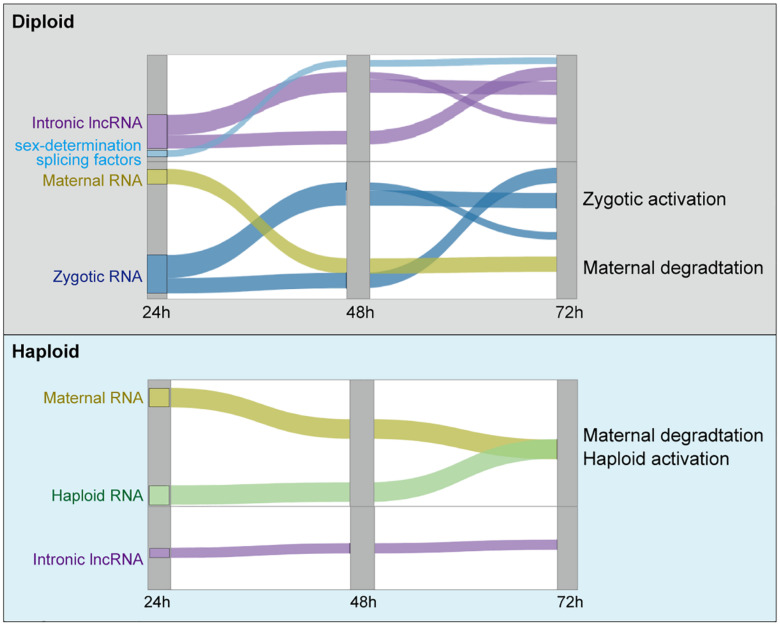

## Introduction

In metazoans, the early stage of embryonic development following fertilization is instructed by the maternal RNAs and proteins from the female gamete, while the zygotic genome remains quiescent transcriptionally. Subsequently, a process known as zygotic genome activation (ZGA) occurs, and ZGA is known to tightly associated with the degradation of maternal instructions. This transcriptional switch drives the early development of all metazoans, and is known as the maternal-to-zygotic transition (MZT; Tadros and Lipshitz, 2009; Langley et al., 2014; Lee et al., 2014; Despic and Neugebauer, 2018). Recent progresses in single-cell RNA-seq and single-cell imaging in the living embryos of model organisms have advanced our understanding of spatio-temporal transcription and chromatin dynamics during the MZT to unprecedented depths (Briggs et al., 2018; Farrell et al., 2018; Wagner et al., 2018). Notably, current knowledge about ZGA during embryonic development has largely been obtained from studies of fertilized eggs; so, it remains unclear how naturally unfertilized eggs coordinate their genomic activation and embryonic development, as for example in haplodiploid animals like rotifers, spider mites, and honeybees.

About 15–20% of animal species are haplodiploid (Bull, 1983; Hedrick and Parker, 1997). In haplodiploid animals, the fertilized diploid embryos generally develop into females while unfertilized embryos develop into males. Western honeybees (*Apis mellifera*) are probably the best-studied haplodiploid animals; these well-known social insects are a model for ecological and social behavioral studies and are also of enormous economic importance because of their essential contributions to pollination in agriculture (Robinson et al., 2005; Honeybee Genome Sequencing., 2006; Mattila and Seeley, 2007; Wallberg et al., 2014). Under natural conditions, the queen very precisely deposits two different types of eggs into hexagonal differentially sized cells of a comb: fertilized (female/worker; diploid) eggs are placed into smaller cells, while unfertilized (male/drone; haploid) eggs are placed into larger cells (Ratnieks and Keller, 1998). The embryonic stage of honeybees – lasting about 70 h – starts after the egg is laid (“*AEL*”) by the queen and ends before the new larvae hatch (Fleig and Sander, 1985, 1986). The developmental times for development from embryos to through the larval and pupal stages before emerging as adults differs markedly for queens (16 days), workers (21 days), and drones (24 days), although there are no phenotypic differences in embryos or throughout the first four larval stages (Bertholf, 1925; Rembold et al., 1980).

It seems to generally be the case in insects that the sex determination occurs based on alternative splicing (AS) regulation of the sex-determination genes (SDGs) (Gempe and Beye, 2011), which have been exemplified in fruit fly and honeybee (Salz, 2011). In the model species *Drosophila*, transcriptional activation of the sex-determination splicing factor *Sex lethal* (*Sxl*) occurs in female embryos but not males (Bell et al., 1988). *Sxl* controls AS of *transformer* (*tra*), which (together with *tra2*) in turn controls the AS of the *doublesex* (*dsx*) in a female-specific manner (Schutt and Nothiger, 2000; Salz and Erickson, 2010). The sex determination mechanism of honeybee (*A. mellifera*) is partly conserved with *Drosophila* in AS hierarchy, but have its haplodiploid-specific SDGs. There are two honeybee-specific SDGs – *complementary sex determination* (*csd*) and *feminizer* (*fem*) – that control the female-specific AS of *Am-dsx*, thus determining the female developmental pathway in honeybee during embryonic development (Beye et al., 2003; Hasselmann et al., 2008). Both *csd* and *fem* encode SR-type splicing factors which are *tra* homologs (Beye et al., 2003; Hasselmann et al., 2008; Gempe et al., 2009). Two copies of *Sxl* are present in *A. mellifera*, but they all have no obvious sex-determining function despite being conserved (Dearden et al., 2006). *Am*-Tra2 proteins are required to promote female splicing of *fem* and *Am-dsx* as well as the male splicing of *fem*, which are distinct from its function in *Drosophila* (Nissen et al., 2012). So far, the specificality of haplodiploid animals in sex-determination remains further revealed.

An understanding is emerging that the complexity of the transcriptional landscape in all animals is increased by the presence of long non-coding RNAs (lncRNAs) (Quinn and Chang, 2016). LncRNAs are noncoding RNA sequences of > 200 nucleotides, and these molecules can act as signals, decoys, guides, or scaffolds to regulate the expression of their target genes (Devaux et al., 2015). In addition to the intergenic regions and antisense strands of protein coding genes, intronic regions have been proposed as reservoirs for sequences encoding lncRNAs (Morris and Mattick, 2014; Mattick and Rinn, 2015). For example, the intronic lncRNA CHRF acts as a decoy of miR-489 and thereby functions to stimulate cardiac hypertrophy (Wang et al., 2014). Tadano et al. (2009) reported a honeybee lncRNA, *Nb-1*, which is encoded by the antisense strand of an intron of another multi-exonic lncRNA; its expression dynamics in worker (female) brains are associated with the division of labor in normal colonies (Tadano et al., 2009). LncRNAs are highly regulated during embryonic development (Batista and Chang, 2013; Fatica and Bozzoni, 2014) as well as in the post-natal brain development, wherein sex-specific expression of lncRNA expression has been observed (Liu et al., 2017). LncRNAs expressed in the promoter region has been reported to influence *Sxl* expression in *Drosophila* (Mulvey et al., 2014). The retrotransposon LINE1 specifically expressed in the pre-implantation mouse embryo was recently reported to encode a nuclear RNA regulating transcriptional program specific to the mouse 2-cell embryo (Percharde et al., 2018). LINE RNA represses the DUX-family transcription factors that regulate ZGA in placental mammals (De Iaco et al., 2017). Further, in female mouse cells, *Xist* encodes a non-coding RNA that initiates the chromosomal silencing process of X inactivation early in embryonic development [at the 4- to 8-cell stage during which ZGA occurs (Wutz et al., 2002; Jeon and Lee, 2011; Chu et al., 2015; McHugh et al., 2015; Wang et al., 2016; Borensztein et al., 2017; da Rocha and Heard, 2017; Sunwoo et al., 2017; Sahakyan et al., 2018]. However, any involvement of lncRNAs in sex-determination and embryogenic development of haplodiploid animals has not been reported yet.

In this study, we used a single-embryo RNA-seq strategy to explore the transcriptional dynamics of both mRNAs and lncRNAs, with the aim of characterizing the differences between the diploid female and haploid male *A. mellifera* embryos. We sampled embryos at 24, 48, and 72 h *AEL* from both larger and smaller cells. We defined three waves of female-specific ZGA of thousands of mRNAs and intronic lncRNAs between 24 and 72 h *AEL*, wherein the transcriptional activation of *csd*, *fem*, and *dsx* occurred between 24 and 48 h. The haploid genome activation occurs 1 day later, lacking the activation of female-specific SDGs and lncRNAs. Instead, expression of the highly abundant *Nb-1* was increased during haploid (male) genome activation. Moreover, intron-retention shows strong sex-specific dynamics during embryonic development.

## Materials and Methods

### Sample Collection

The honeybee, *A. mellifera*, colonies were maintained at the Institute of Apicultural Research, Chinese Academy of Agricultural Sciences, Beijing, China, according to standard beekeeping technique. To control the egg laying, a mated queen was confined on an egg-free frame for 3 h before placed back into the hive. Developing Embryos were collected at 24, 48, and 72 h after laying, and 3 embryos (biological replicates) were sampled for each time point. Worker (female) and drone (male) embryos (Supplementary Figure 1B) were collected from the distinct type of frame (Supplementary Figure 1A) laid by the same queen. We sampled two groups of worker (female) and drone (male) embryos laid by two different honeybee queens. Then a total of 36 embryos were collected for single-embryo RNA-seq.

### Whole Mount *in situ* Hybridization

Embryos were fixed in 4% paraformaldehyde for 4 h, dehydrated in methanol, and stored in 100% MeOH at −20°C until use. Samples were rehydrated, pretreated with proteinase K, and hybridized with DIG-labeled RNA probes followed by washing with 2 × SSC/50% formamide three times at 70°C. The signal was detected using an alkaline phosphatase-conjugated anti-DIG antibody (11093274910; Roche). Tissues were incubated in the BM Purple alkaline phosphatase substrate (11442074001; Roche) at 4°C for several hours until the signal developed to the desired extent. Probes for each gene were generated using DIG RNA Labeling Kit (11 175 025 910; Roche). Primers for probe generation were listed in Supplementary Table 4.

### Real-Time Quantitative Polymerase Chain Reaction (PCR)

In this study, to elucidate the validity of the RNA-seq data, real-time quantitative PCR (RT-qPCR) was performed for some selected genes, and normalized with an external reference sequence. The same RNA samples for RNA-seq were used for qRT-PCR. The PCR conditions are consisted of denaturing at 95°C for 10 min, 40 cycles of denaturing at 95°C for 15 s, annealing and extension at 60°C for 1 min. PCR amplifications were performed in triplicate for each sample.

Meanwhile, RT-qPCR assay was used to analyze alternative polyadenylation sites (APAs) for *tra2* gene. To detect one of the alternative isoforms, one primer is designed in the alternative exon, and an opposing primer is designed in a constitutive exon. To detect the other of the alternative isoform, a boundary-spanning primer for the sequence encompassing the exon–exon junction with the opposing primer in a constitutive exon is used. Relative quantification was achieved by normalization to the amount of internal reference with the 2^–ΔΔ*Ct*^ method (Livak and Schmittgen, 2001). We have followed the MIQE guidelines to present RT-qPCR results (Bustin et al., 2009). Primers for qPCR analysis were listed in Supplementary Table 4.

### Library Construction

High-quality, full-length cDNA was generated directly from single honeybee embryo by the SMART-Seq v4 Ultra Low Input RNA Kit for Sequencing (Cat. Nos. 634890). cDNA was amplified by LD PCR. RNA can be transcribed by T7 promoter from ds cDNA, RNA was treated with RQ1 DNase (promega) to remove DNA. Quantity of the purified DNA were determined by Qubit. For each sample, 200 ng RNA was used for RNA-seq library preparation. RNAs were iron fragmented at 95°C followed by end repair and 5′ adaptor ligation. Then reverse transcription was performed with RT primer harboring 3′ adaptor sequence and randomized hexamer. The cDNAs were purified and amplified and PCR products corresponding to 200–500 bps were purified, quantified and stored at −80°C until used for sequencing.

For high-throughput sequencing, the libraries were prepared following the manufacturer’s instructions and applied to NextSeq 500 system for 151 nt pair-end sequencing (ABlife Inc.). Six biological replicate RNA-seq samples from two honeybee queens were obtained for each of the three time points.

### Raw Data Processing

The raw reads were first removed of their adaptor sequence by cutadapt (Martin, 2011)(v1.8.1). The sense and antisense of t7 promoter were also cut. To remove other contamination during library construction, the left and right most 15 nt bases were also removed. The bases whose quality were lower than 20 were also removed by FASTX-Toolkit (v0.0.14). The reads length less than 16 nt were filtered. Last, the front 65 bp of the reads were kept as cleaned reads. The filtered reads were then mapped to the *A. mellifera* (Amel_4.5) genome sequence (Honeybee Genome Sequencing., 2006) (Honeybee Genome Sequencing., 2006)^1^ by TopHat2 with read-edit-dist 4-N 4. We also aligned the filtered reads to the new genome assembly sequence of honey bee (Amel_HAv3) (Wallberg et al., 2019), and found only one percentage improvement of total aligned reads. Because there was no untranslated region (UTR) annotation in new genome assembly, we used Amel_4.5 aligning result and only extracted the reads that were unambiguously aligned to the genome. We then calculated reads number and RPKM value (RPKM represents reads per kilobase and per million) for each gene (Mortazavi et al., 2008).

### Measure of Poly-A Tails Length

To measure of poly-A tails length, we used a published method, named pA-finder (Yu et al., 2020). Sequencing reads with poly-A tails were kept for analysis. Preliminary poly-A regions were defined with at least 5A sequences at both sides. Other parameters were the same as described in Yu et al.

### Differentially Expressed Genes Analysis

After getting the expression level and reads of all genes in all samples, differentially expressed genes between the paired groups were analyzed by using edgeR (Robinson et al., 2010) embedded in R software. We calculated the significance *p*-value based on the model of negative binomial distribution for each gene. We also estimated fold changes of gene expression within the edgeR statistical package. The threshold value for Differential Expressed Gene (DEG) has been set as fold change > 2 or < 0.5 and *p*-value < 0.01.

### Alternative Splicing Analysis

The splicing junction (SJ) reads with gaps while aligned to the genome by TopHat2 were obtained. We defined known SJs from all annotated isoforms in honeybee genome annotation and others not matched annotated junction sites are novel SJs. The reads walking through the site and each side of intron boundary region with no less than 8 nt were defined as boundary reads. The junctions located inside the coordinates of annotated genes were regarded as genic SJs, which can be classified into one of the nine types of AS events (ASE). Seven of the canonical ASEs were skipped exons (ES), cassette exon (CE), alternative 5′-splice sites (A5SS), alternative 3′-splice sites (A3SS), mutually exclusive exons (MXE), alternative first exons (AFE or 5′MXE) and alternative last exons (ALE or 3′MXE) according to the models described previously (Wang et al., 2008). AS regulation analysis was performed by running *in silico* ABL as pipeline as previously described (Xia et al., 2017).

### LncRNA Prediction and Direction Identification

To systematically analyze the lncRNA expression pattern, we used a pipeline for lncRNAs identification similar as we previously reported (Liu et al., 2017), which was constructed based on the cufflinks software (Trapnell et al., 2012). All steps of the pipeline have been shown in Supplementary Figure 2C. We calculated coding potential score (CPS) to filter the coding potential transcripts (Kong et al., 2007). When filtering the single exon lncRNAs, we set two thresholds: 1,000 nt to obtain longer single exon lncRNAs, and 500 nt to keep more single exon lncRNAs.

We then used the polyadenylation signal to detect the transcriptional direction of lncRNAs. Firstly, we selected reads whose tail was with more than 10 A or T, allowing 0.1 error rate. Then we aligned the reads longer than 20 nt to the genome sequence. We discarded the aligned reads if their poly (A) tails come from the genomic sequence (internal polyA). The terminal aligned positions of reads were clustered together if they were located within 20 bp on the genome locus, and the clusters were regarded as polyadenylation sites (PASs). We then compared the genomic locations of predicted lncRNAs and PASs. If the PASs were located at the downstream of lncRNAs, the direction of lncRNAs were the same with reference direction, and vice versa. If there were poly (T) sites, the direction of lncRNAs was reversed.

### Co-expression Analysis

To fully understand the gene expression pattern during embryogenesis of honeybee, we applied weighted gene co-expression network analysis (WGCNA; Langfelder and Horvath, 2008) to cluster genes that have similar expression pattern with default parameters. All expressed genes were used as input data. Eigengenes for each clustering module was used as the representative expression pattern of genes in each module. To explore the regulatory mode between lncRNAs and their host mRNAs, we calculated the Pearson’s correlation coefficients (PCCs) between them and classified their relation into three class: positive correlated, negative correlated and non-correlated based on the PCCs value.

### *Drosophila* Transcriptome Analysis

We analyzed the transcriptome data of *Drosophila* from Lott et al. (2011). Transcriptome data were aligned to *Drosophila* genome (BDGP6.22). Gene annotations were download from Ensembl database (Ensembl release 95). The analysis pipeline was the same as transcriptome data from honeybee. Sex-determination genes were annotated by blast software (Altschul et al., 1990).

### Other Statistical Analysis

Grouped by the female and male during the different embryonic development, the RPKM value of all the genes in all samples were used to conduct the principal component analysis (PCA), which was performed by R package prcomp (Ma and Dai, 2011) to show the clustering of samples with the first two components. After normalizing the reads by TPM (tags per million) of each gene in samples, in house-script (sogen) was used for visualization of next-generation sequence data and genomic annotations. The pheatmap package^1^ in R was used to perform the clustering based on Euclidean distance. To assess the functional enrichment of a given gene set, we aligned the protein sequence of honeybee to the gene ontology (GO) and KEGG databases. Then we used hypergeometric test to calculate the enrichment of a given gene set, and all genes were regarded as background.

## Results

### Activation of Developmental Transcription Programs Differs Between Diploid Female and Haploid Male Embryos

A previous study of seven transcriptomes from honeybee (*A. mellifera*) within 24 h *AEL* only revealed zygotic activation of a small number of genes (Pires et al., 2016). To study ZGA dynamics in honeybee, we here used single-embryo RNA-seq to explore the transcriptional landscape and differences between diploid female and haploid male honeybee embryos from 24 to 72 h *AEL*. The single-embryo RNA-seq data we analyzed from the developing *Drosophila* embryos for mitotic cycles 10–14, thereby collectively covering the embryonic stages after cleavage up until the completion of cellularization.

**FIGURE 1:**
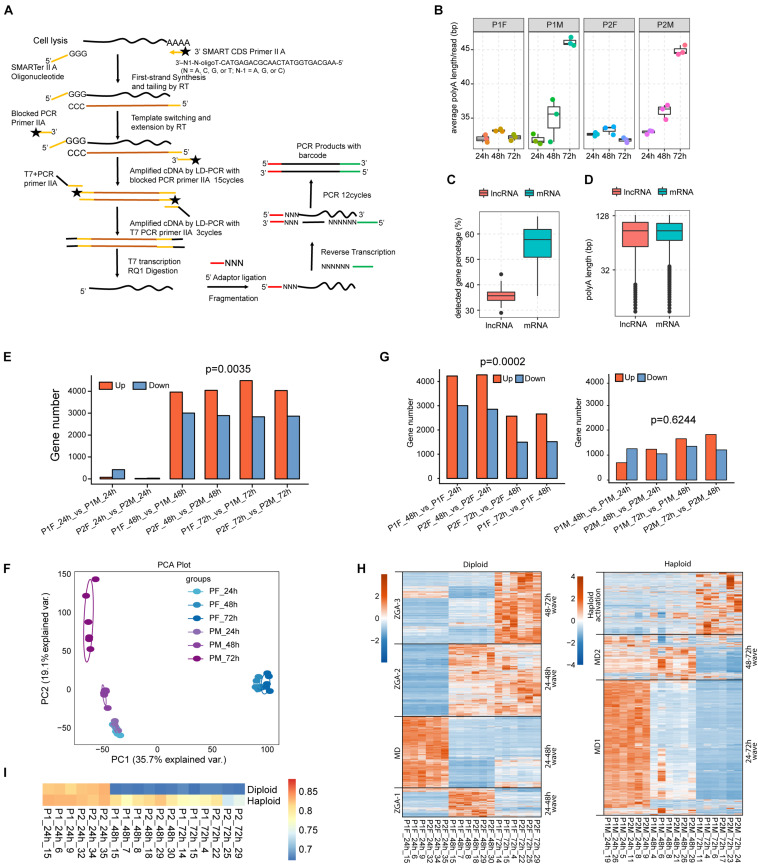
The difference in the developmental transcription program activation between diploid female and haploid male embryos. **(A)** Schematic plot showing the workflow of single-embryonic library construction. **(B)** Box plot of average poly (A) length showing that the poly (A) length of male embryos increased during embryonic development (*p*-value < 0.001, one way ANOVA test for both datasets), while that of female kept constant (*p*-value > 0.01, one way ANOVA test for both datasets). **(C)** Box plot showing that the percentage of lncRNAs with poly (A) tails was lower than that of mRNAs (*p*-value < 0.0001, *t*-test). **(D)** Box plot of the poly (A) length showing that there was no significant difference of average poly (A) length (middle line in box plot) between lncRNAs and mRNAs (*p*-value > 0.05, *t*-test). **(E)** Barplot of DEGs between male and female embryos. More up-regulated DEGs were observed in female embryos after 24 h (up-regulated DEGs number vs. down-regulated DEGs number, *p*-value = 0.0035, *t*-test). **(F)** PCA analysis showing the divergent gene expression pattern between male and female embryos after 24 h. **(G)** Barplot of DEGs between adjacent time points for female (left) and male (right) embryos, respectively. More up-regulated DEGs were observed in female embryos during development (up-regulated DEGs number vs. down-regulated DEGs number, *p*-value = 0.0002, *t*-test). **(H)** Hierarchical clustering heatmap showing the expression waves of all DEGs detected in this study. Male and female samples were separated for the plotting. Expression waves were separated by black lines. The dendrogram was not shown. MD: maternal degradation. **(I)** The Pearson’s correlation coefficients (PCCs) was shown by heatmap for each of the single-embryonic samples with the published oocyte sample (Pires et al., 2016).

Our single-embryo RNA-seq approach was designed to study the transcriptional dynamics of both mRNAs and lncRNAs in male and female honeybee embryos (Figure 1A) (see section “Materials and Methods”). A total of 36 single-embryo RNA-seq transcriptomes were generated from embryos laid by 2 queens (P1 and P2). Among the 18 transcriptomes from each of the two queens, three female embryos (F) were collected from the female cells and three male embryos (M) from the male cells at each time point of 24, 48, and 72 h *AEL* (Supplementary Figures 1A,B). The sequencing depth and expression profiles of the 36 single-embryo RNA-seq transcriptomes are summarized in Supplementary Tables 1, **2**, respectively. In each sample, around 73–89% of honeybee genes were detected (RPKM > 0.1). Gene expression was normalized using a dividing size factor by the DESeq method (Anders and Huber, 2010). The 75th percentile levels of genes were identical between samples after normalization (Supplementary Figure 1C), similar with previously published single-embryo RNA-seq data from *Drosophila* (Lott et al., 2011) (Supplementary Figure 1D). Almost all of the currently known lncRNAs (99.26%, 2412/2434) and most of the annotated protein coding genes (98.23%,10436/10623) were expressed at least in one honeybee embryonic sample (Supplementary Figures 1E,F), indicating that single-embryo RNA-seq data from the developing honeybee embryos was of sufficient quality to enable further study of their mRNA and lncRNA transcriptomic dynamics. We analyzed the dynamics of the polyA-tails of embryonic transcripts by counting the A numbers of reads aligned to the end of transcripts (Yu et al., 2020), demonstrating that the poly(A) tail lengths of male embryos increased during embryonic development while those of female embryos did not change (Figures 1B–D).

Principal component analysis, PCCs, and DEG analysis based on the edgeR method (Robinson et al., 2010) using FDR < 0.05 and |log_2_FC| > 1 were performed to explore major transcriptional trends during honeybee embryonic development (Figures 1E,F and Supplementary Figures 2A,B). The transcriptomes of all 12 embryos, 6 for each sex, at 24 h *AEL* were closely clustered, and they were highly distinct from all the other embryos; the latter transcriptomes for both female and male embryos subsequently exhibited tremendous divergence in their expression patterns (Figure 1F and Supplementary Figures 2A,B). Diploid female embryos (both 48 and 72 h) clustered and were well-separated by the first component (35.7% explained variation), while the haploid male embryos were separated by the second principal component (19.1% explained variation) (Figure 1F).

DEG analysis revealed that more genes were significantly upregulated than downregulated between 48 and 24 h, and between 72 and 48 h (*p*-value = 0.0002, *t*-test), and the similar results were observed for two independent sample groups (*n* = 2) laid by two different queens (Figure 1G, left panel). These findings are consistent with strong transcriptional activation during female embryonic development. Hierarchical clustering analysis revealed three waves of female-specific activation: that proceeded from 24 to 48 h *AEL* (Wave 1, 916 genes), from 24 to 72 h *AEL* (Wave 2, 2,731 genes), and from 48 to 72 h *AEL* (Wave 3, 2,649 genes) (Figure 1H, left panel). Wave 1 is characterized by a strong activation between 24 and 48 h *AEL*, which was then re-silenced after 48 h *AEL*. In contrast, the activation in Wave 2 lasted until 72 h *AEL*. The activation in Wave 3 primarily occurred between 48 and 72 h *AEL* (Figure 1H, left panel).

In male embryos between 48 and 24 h *AEL*, there were about 2 × more differentially expressed downregulated genes (569) than upregulated genes (Figure 1G, right panel), supporting the idea of maternal repression rather than a “haploid activation” (Figure 1G, right panel). Moreover, the downregulation of maternal RNA occurred more slowly in haploid than in diploid embryos (Figures 1F,I). The poly(A) tail lengths of female embryos (Figures 1B–D) were also consistent with slower maternal degradation. There was a single activation wave in the haploid embryos that occurred between 48 to 72 h *AEL* (Figure 1H, right panel).

### A Majority of the lncRNAs Expressed During Embryonic Development Are Intronic

**FIGURE 2:**
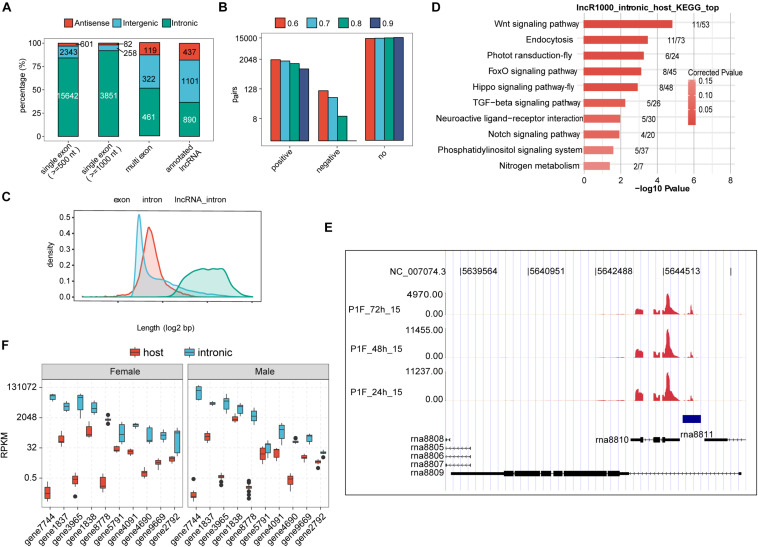
Embryonic honeybee lncRNAs are dominantly intronic. **(A)** Bar plot showing the genomic locus distribution of the newly identified and previously annotated lncRNAs in honeybee. Single exonic lncRNAs were selected with two thresholds (1,000 and 500-nt). The genomic distribution of the newly identified single-exonic, multiple-exonic lncRNAs and the annotated lncRNAs is shown. **(B)** Bar plot showing the number of co-expressed pairs between intronic lncRNAs and their host mRNAs by PCC calculation. Four PCC thresholds were shown in the figure. No indicated the pairs that were not satisfied with the threshold. **(C)** Density of the length distribution of exons and introns of honeybee. Introns containing lncRNAs (500-nt threshold for single exon lncRNAs) were plotted separately. The length density distribution was generated by density function in R. **(D)** The top 10 functional enriched KEGG pathways of genes encoding intronic lncRNAs (1,000-nt threshold for single exon lncRNAs). **(E)** Reads density plot showing the prevalent transcription in intronic region. Blue frame indicates the newly predicted transcript in the intronic region of rna8809. The annotated intronic lncRNAs rna8810 and rna8811 were transcribed extensively as well. **(F)** Box plot showing the higher expression levels of intronic transcripts than their host exonic mRNAs in both female and male embryos.

Before we profiled the expression dynamics of embryonic honeybee lncRNAs, we decided to perform a *de novo* lncRNA prediction from the 36 single-embryo transcriptomes. We here adopted a cufflinks-based lncRNA identification procedure similar to a previous report (Liu et al., 2017) (Supplementary Figure 2C). We thusly identified 902 multi-exonic lncRNAs and 4,191 candidate single-exonic lncRNA with a minimum length of 1,000-nt. Most of the single-exonic lncRNAs (91.9%) were derived from the intronic regions, and 51.1% of multi-exonic lncRNAs were from the intronic regions. Note that most of the annotated lncRNAs from honeybees reported in previous studies are multi-exonic; only 36.7% were reported to be from intronic regions (Figure 2A).

Interestingly, the previously reported lncRNA *Nb-1*, whose expression dynamics in worker brains was associated with the division of labor in normal colonies, is 599-nt in length, single-exonic, and located in the intronic region of an annotated multi-exonic lncRNA (*LOC102654021*) (Tadano et al., 2009). Seeking to identify lncRNAs with similar characteristics to *Nb-1* lncRNA, we retained all of the predicted single-exonic lncRNA genes longer than 500-nt; 18,568 single-exonic lncRNAs, including *Nb-1*, were thusly obtained (Figure 2A). Notably, the same criteria resulted in a dramatically smaller number of novel *Drosophila* embryonic lncRNAs (Supplementary Figure 2D). Most of the annotated *Drosophila* lncRNAs were single exonic, and generally smaller than 1,000-nt in length (Supplementary Figure 2E). Our results thus indicate that *Drosophila* embryos expressed an order of magnitude fewer lncRNAs than do honeybee embryos.

To validate the authenticity of predicted lncRNAs and investigate the relationship between lncRNAs and their host gene, we further analyzed the poly(A) tail sequences at the 3′-end to determine the lncRNA direction (Supplementary Figure 2F). Because sequences with poly (A) tail were only a small fraction of total aligned reads, we thus only determined the transcription direction of 1,382 (7.4%) lncRNAs. Among these, 1,159 were intronic lncRNAs, and 1,143 were from protein-coding genes. Interestingly, 37.2% (310) were expressed from the antisense direction of a protein coding gene (Supplementary Table 3), suggesting an independent expression manner of these lncRNAs. Based on gene annotation of honeybee, half of the genome sequences are intronic regions (Supplementary Figure 2G), with a large population (27,602, 14.47%) longer than 2,000 nt. The majority of honeybee lncRNAs expressed during embryonic development were from long introns (Figure 2C). Expression correlation analysis showed that the expression of most (over 85%) intronic lncRNAs was not correlated with the expression of their host genes (Figure 2B and Supplementary Figure 3A), indicating their independent transcription from their host genes. By dividing single exon lncRNAs into different classes according to their expression, we found the number of higher expressed lncRNAs was not decreased but increased in female samples (Supplementary Figure 3B). Notably, the host gene containing intronic lncRNAs in honeybee embryos were highly enriched in developmental-control related signaling pathways (Figure 2D). Among the top-10 genes whose introns harboring mostly expressed lncRNAs, four encode neuron-related functions, including Neurexin 1 (gene3965), neuromusculin (gene8778), discs large 1 (gene9669), and an RNA binding protein RBFox1 (*A2bp1*, gene2792). The expression from these 10-top host gene exons was about one-to-six orders of magnitude lower that from their intronic RNA regions (Figures 2E,F).

### Honeybee lncRNAs Are Extensively Activated in Female but Not Male Embryos

**FIGURE 3:**
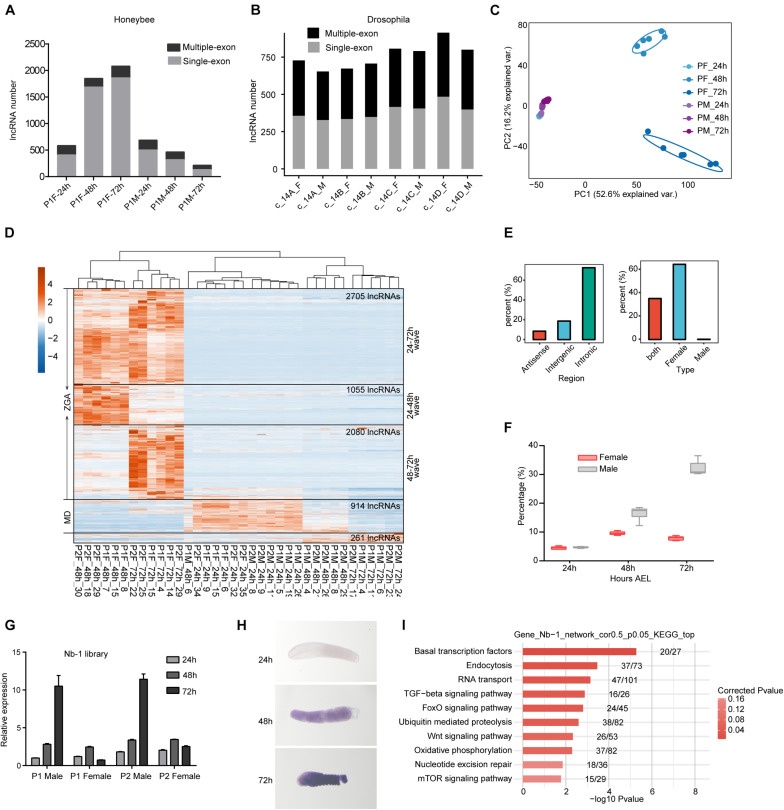
LncRNAs are extensively activated during the embryonic development of honeybee female embryos. **(A)** The numbers of detected lncRNAs (1,000-nt threshold for single exon lncRNAs) were increased in female embryos, and decreased in male embryos. In each sample group, we randomly selected 20 million mapped reads from each sample to perform lncRNAs prediction pipeline. Results of 18 samples from one queen were shown. The results of other 18 samples were similar. **(B)** The numbers of detected lncRNAs were not obviously changed in female and male embryos of fruit fly. The same analysis method was executed as in panel (A). **(C)** PCA analysis showing the divergent lncRNA expression pattern specifically for female embryos after 24 h (1,000-nt threshold for single exon lncRNAs). **(D)** Hierarchical clustering heatmap showing the zygotic expression waves of lncRNAs in female embryos (1,000-nt threshold for single exon lncRNAs). **(E)** The percentage of intronic lncRNAs (left) and female specific lncRNAs (right) among the zygotic activated lncRNAs shown in panel **(D)**. **(F)** Boxplot showing the increased percentage of *Nb-1* reads relative to the total reads aligned to gene regions in honeybee female and male embryos. **(G)** RT-qPCR validation of *Nb-1* expression showing high consistence with the sequenced libraries. **(H)** *In situ* hybridization experiment validated the robust increase of *Nb-1* expression in male embryos. **(I)** Bar plot showing the top ten enriched KEGG pathways for genes whose expressions were correlated with *Nb-1* (*p*-value < 0.05 and PCCs > 0.5).

We then profiled the expression dynamics of embryonic honeybee lncRNAs. We observed that the detected number of lncRNAs was increased during the developmental course in female embryos but decreased in male embryos (Figure 3A). This large sex-difference was not observed in *Drosophila* (Figure 3B). PCCs and PCA analyses of the expression profiles of lncRNAs showed that their expression patterns in female honeybee embryos at 24, 48, and 72 h *AEL* were highly divergent. In contrast, the global lncRNA expression patterns in male embryos were strikingly similar (Figure 3C and Supplementary Figure 2C). Heatmap clustering also revealed the globally-activated expression of honeybee lncRNAs specifically in female embryos, although maternally repressed lncRNAs were similar in both female and male embryos (Figure 3D). Importantly, there were three obvious waves of lncRNA activation for 24–48, 24–72, and 48–72 h, just like what we observed in the mRNA data for diploid females (Figures 3D, left, 1H, left). Statistics of the lncRNAs specifically activated in zygotic female embryos showed that 72.7% of them were transcribed from intronic regions and 64.6% were specifically expressed in female embryos (Figure 3E).

The most highly expressed lncRNAs in both female and male was *Nb-1* lncRNA, which occupied as high as 36.49% of the total reads mapped to the previously annotated gene regions (Figure 3F). Strikingly, *Nb-1* expression was increased in both sexes at 48 h *AEL*; however, its expression increased robustly and specifically in male embryos at 72 h *AEL* (Figure 3F). We validated the male-specific increase in the expression of *Nb-1* by both qPCR and *in situ* hybridization (ISH) experiments (Figures 3G,H). Although *Nb-1* expression was highly regulated, expression of its host gene was consistently low (Supplementary Figure 3C). To predict potential targets of lncRNA *Nb-1*, we used co-expression network analysis, and revealed a negative correlation between *Nb-1* and 95% of its co-expressed genes (*p*-value < 0.05 and | PCCs| > 0.5). The co-expressed genes showed enrichment for basal transcription factors, endocytosis, RNA transport, as well as for several developmental-control signaling pathways (Figure 3I), implying the potential functions of *Nb-1* in embryonic development of honeybee.

### The Dynamics of Embryonic Intron Splicing Are Associated With the Expression of Known Sex-Determination Splicing Factors Like *csd*, *fem*, *tra2*, and *Sxl*

**FIGURE 4:**
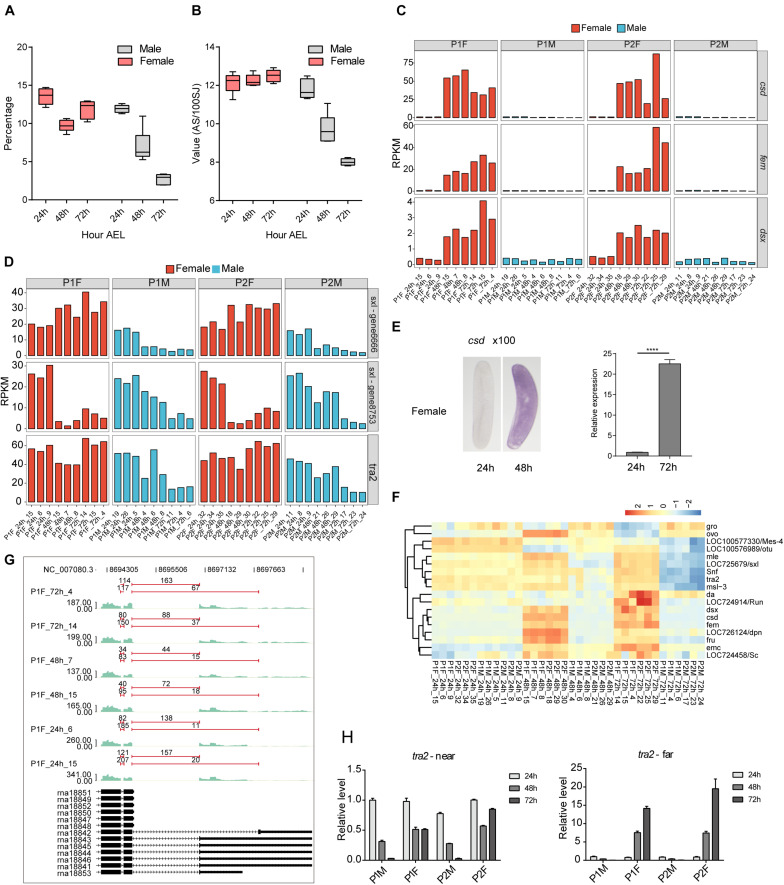
Alternative splicing (AS) is regulated in a sex-specific pattern during honeybee embryonic development. **(A)** Boxplot showing the percentage of spliced reads among total uniquely mapped reads for each group. **(B)** Boxplot showing the detection efficiency of AS events in female and male eggs. We used the detected ASEs number per 100 SJs as the detection efficiency. **(C)** Bar plot showing the time-dependent expression dynamics of *Sxl*, *tra*, and *dsx* in male and female embryos. **(D)** Bar plot showing the time-dependent expression dynamics of *tra2* and two copies of *Sxl* in male and female embryos. **(E)** ISH and RT-qPCR validation results of the increased expression level of *csd* gene after 24 h *AEL* in female embryos. The embryos were amplified by 100 times, and the purple color represented the intensity signal of *csd* (ISH, left). **(F)** Hierarchical clustering heatmap of the expression level of SDGs revealed two types of expression patterns: maternal expressed (upper cluster) and zygotic activated (lower cluster) **(G)** Reads density plot showing the APA regulation of *tra2* transcripts in female embryos. The numbers above the red line indicate the counts of splicing junction reads. **(H)** Bar plot showed the RT-qPCR validation of APA usage for *tra2* transcripts. Left panel showing the proximal PAS signal, and right panel for distal PAS signal.

Our observation of a large population of intronic lncRNAs, in both sense and antisense strands, suggests that there may be interplay between intron splicing and lncRNA expression. Interestingly, global splicing overview revealed that splicing efficiency was relatively constant in diploid females but was time-dependently reduced in haploid males during embryonic development (Figure 4A). Further, the detected AS events were constant in females, but substantially decreased in male embryos, and did so in a time-dependent manner (Figure 4B). The large difference in the AS activity between female and male embryos was coincident with the activation of thousands of lncRNAs in female but not male embryos.

We here compared our honeybee data to a published single-embryo transcriptomes of fruit fly (see section “Materials and Methods”) to delineate any links between the expression of SDGs and the expression or AS dynamics of mRNAs and lncRNAs at each of the embryonic developmental stages. In *Drosophila*, we noted that *Sxl* expression was low in both males and females at mitotic cycle 10–13, but was robustly activated at the 14th mitotic cycle specific in females (Supplementary Figure 3D), indicative of a strong sex-specific zygotic activation. Expression of *tra2* showed no sex-specificity, showing a time-dependent maternal degradation in both sexes of diploid embryos (Supplementary Figure 3D). Expression of *dsx* was at a consistently very low level, yet zygotic activation occurred in both sexes of diploid embryos from mitotic cycle 11–14 in a time-dependent manner (Supplementary Figure 3D).

In honeybee embryos, *csd*, *fem*, and *dsx* showed very low expression at 24 h *AEL*, yet the expression of all three of these genes was strongly activated in diploids from 24 to 48 h *AEL*, which did not occur in haploids (Figure 4C). ISH and qPCR confirmed the induced expression of *csd* in females (Figure 4E). The expression of the other two aforementioned sex-determination splicing factors (*tra2* and *Sxl*) was high in 24 h embryos and oocytes (Figure 4D and Supplementary Figure 3E), indicative of their maternal expression. We found evidence for the maternal degradation of transcript from one copy of *Sxl* gene (gene8753) in diploid honeybee embryos and for all the *tra2* and *Sxl* genes in the haploid embryos. Note that the expression of the *tra2* gene and the one *Sxl* copy (gene6666) was constantly high throughout embryonic development in female (Figure 4D). This robust transcriptional activation of the female-specific splicing factors *csd* and *fem*, viewed alongside the constantly high levels of *tra2* and *Sxl* in female embryos (but not male embryos) from 24 to 48 h *AEL*, together support the findings that highly efficient splicing occurs, specifically in females, throughout embryonic development.

We also examined the expression levels of all 18 genes previously reported to function in sex-determination (Sanchez, 2008; Salz, 2011). Clustering analysis of their expression profiles revealed two distinct groups: one group appeared strongly influenced by maternal expression while the other seemed to be controlled by the female-specific activation waves (Figure 4F). To further verify this transcriptional profile, we re-analyzed our RNA-seq data in the context of a previously published proteome dataset from the embryos of *A. mellifera* that were collected at similar time-points during embryogenesis (Fang et al., 2015) and found that our data included mRNA transcripts corresponding to 98.48% of the proteins identified in that dataset (Supplementary Figure 3F). Notably in the proteome dataset, three SDG proteins – Gro, Snf, and an Sxl homolog (gene6666) – were detected at all stages of male and female embryos (Supplementary Figure 3G). However, the Tra2 protein was specifically detected at 48 and 72 h *AEL* only in female embryos, supporting the result from our single embryo RNA-seq.

Alternative splicing regulation of SDGs is known to be key regulatory influence during sex determination (Sanchez, 2008; Salz, 2011; Haussmann et al., 2016), so we detected AS events for the *csd*, *fem*, *dsx* and other SDGs in female embryos and found that splicing isoforms that were consistent with previous reports (Hasselmann et al., 2008; Gempe et al., 2009) (Supplementary Figure 4 and Supplementary Table 5). There are multiple AS events observed among these SDGs: *csd* contain 8 types of ASEs, including MXE, alternative 3′ splice site (A3SS), and intron retention events (IR); we only observed two IR events of *fem* in our data; *dsx* had ASEs of IR and alternative 5′ splice site (A5SSA) (Supplementary Table 5). The fruitless (*fru*) gene were reported functioning in the sex-specific neuronal differentiation of *Drosophila* (Ryner et al., 1996; Demir and Dickson, 2005). We also found the *fru* homolog in honeybee, but no obvious AS differentiation was observed between female and male embryos. We found 16 ASEs in *fru*, including the types of MXE, A5SSA, A3SS, and exon skipping (ES) (Supplementary Table 5). AS of *Sxl* and *tra2* were similar in both female and male embryos (Supplementary Figure 4). However, female and male embryos differed significantly at their respective AS products with the last exon of *tra2*, which was present as two mRNA isoforms with APAs (Figure 4G, *p*-value = 0.003, Fisher’s exact test). At 24 h *AEL*, both female and male embryos primarily used the proximal 3′ss and polyadenylation site, leading to the dominant form of shorter 3′UTR. At 48 and 72 h AEL stages, the usage of the distal 3′ss and polyadenylation site in female embryos was significantly increased, but remained largely unchanged in male embryos (Figure 4G). RT-qPCR experiments validated the APA events as well as the specific usage of the APA site in female embryos (Figure 4H).

## Discussion

Based on our results, we propose a model in which the inactivation of the extra set of chromosomes carried by diploid female honeybees may be controlled influenced by ZGA-specific lncRNA activation during embryonic development. Haploid male embryonic development lacks such a zygotic activation program; moreover, transcriptional activation is weak in haploids, and maternal degradation occurs more slowly than in diploids, which is also observed in *Drosophila* embryos (Bashirullah et al., 1999), suggesting a possible similar mechanism of RNA degradation in *Drosophila* and honeybee. During diploid embryonic development, at least three waves of ZGA occur, with the expression of thousands of protein coding genes. The ZGA-activated expression of sex-determination splicing factors (*csd*, *fem*, *tra2*, and *Sxl*) occurs in the early wave. Importantly, we also found that the ZGA program simultaneously occurred with the expression of thousands of intronic lncRNAs. Based on the regulatory functions of lncRNAs in many species (Morris and Mattick, 2014; Statello et al., 2021), One possible explanation is that these lncRNAs may function to participate in ZGA program in the diploids. Given the lack of ZGA program in haploids, it is consistent that we did not observe zygotic activated lncRNA expression. This model suggests a unique dose compensation mechanism for haplodiploid animals that involves over a thousand lncRNAs which may apparently involve in the expression regulation of genes during the ZGA program (Graphical Abstract).

During embryonic development, MZT controls a coordinated cascade of genetically encoded events (Lee et al., 2014). In fertilized female honeybee embryos, we found that the maternal transcripts are globally degraded from 24 to 48 h *AEL*, and robust ZGA in honeybee occurs in waves throughout the late blastoderm and gastrulation stages ranging from 24 to 72 h (Nelson, 1915; Fleig and Sander, 1985), which resembles the previously reported ZGA program in *Drosophila* (Pritchard and Schubiger, 1996). The process of sex determination in *Drosophila* is known to be controlled by the transcriptional activation of the *Sxl* splicing factor in female embryos (Schutt and Nothiger, 2000) and *Sxl*-initiated short splicing regulation cascade composed of three genes *Sxl*, *tra2*, and *dsx* (Sanchez, 2008; Gempe et al., 2009; Salz, 2011; Bopp et al., 2014; Zhang et al., 2014). From our data, we observed a more complex splicing regulation network comprising *Sxl*, *tra2*, *csd*, *fem*, and *dsx* in honeybee embryos, all of which are under ZGA program control in female embryos. Additionally, we found that *tra2* expression may be controlled via a previously unknown AS-coupled APA. Taken together, it is likely that honeybee and *Drosophila* use evolutionally divergent mechanisms to control *dsx* expression. We propose that the ZGA-activated expressions of sex-determining splicing factors in the early wave may function to support the high splicing efficiency and the intronic lncRNA activation that we observed in diploid embryos.

The involvement of lncRNAs in regulating early embryonic development is conserved among animals (Fatica and Bozzoni, 2014). *Xist/XIST* RNA is directly involved in the repression of chromatin formation; *Xist* is specifically expressed in early female mouse and human embryonic cells to orchestrate X-chromosome inactivation (XCI) (da Rocha and Heard, 2017; Sahakyan et al., 2018). In agreement with a role for lncRNAs in chromosome dose compensation, we here demonstrate that the transcription of thousands of lncRNAs is specifically and temporally observed in female honeybee during embryonic development, implying lncRNA activation may be controlled by the same ZGA program and has the potential to regulate thousands of protein-coding genes.

Our study provides evidence that different genome activation programs are exerted in female vs. male embryos that illustrates that these programs affect very large populations of both mRNAs and lncRNAs. lncRNAs are known to function in establishing cell epigenetic states that control cell fate specification during early embryonic development (Burton and Torres-Padilla, 2014). For example, some lncRNAs regulate the expression of HOX genes, and the latter are transcription factors highly conserved and specify the development embryo body plans (Barber and Rastegar, 2010; Mallo and Alonso, 2013; Fatica and Bozzoni, 2014). A recent study showed the potential contribution of epigenetic differences to sex-biased gene expression in adult males and females of two haplodiploid animals (Wang et al., 2015). It is thus conceivable that the prevalence of sex-specific lncRNA expression during honeybee embryonic development may contribute to the establishment of epigenetic states for different cell fate specification programs that control their later developmental paths toward queens, workers, and drones.

We found here that honeybee introns apparently encode thousands of lncRNAs, and the prevalent transcriptional activation of these intronic lncRNAs during ZGA in female honeybee embryos implies a model in which the female-specific lncRNAs may activate zygotic genome transcription and regulate the gene expression in honeybee. Further studies on the epigenetic level and interaction between lncRNAs and genomic DNA regions of different genes in developing embryos and during other developmental stages of honeybee and other haplodiploid animals could be conducted to explore the lncRNA interaction profiles and mechanisms (Wang et al., 2015; Grath and Parsch, 2016). The zygotic activation of female-specific intronic lncRNAs is supported by the constantly high splicing efficiency and the activation of sex-determining splicing factors specifically in female embryos. The lack of the ZGA during blastoderm formation in haploid males apparently thusly escapes the activation of the female-specific lncRNAs and its attendant lncRNA-mediated gene expression. The insights gained from our study illuminate a new path with many intriguing questions that can be addressed experimentally and can thereby help decipher both the haploid genome activation and the unique female-specific lncRNA mechanisms underlying chromosome silencing and ZGA in these genetically and behaviorally fascinating social insects.

## Data Availability Statement

The raw sequencing data has been deposited in GEO and are accessible through accession numbers GSE101741 for all RNA-seq sequencing reads.

## Author Contributions

LW, YiZ, HZ, and MW conceived the project. MW, XX, FY, and LZ designed and performed the experiments. DC, HZ, YuZ, MW, LZ, CC, and YiZ analyzed the data. XX, LW, QN, and SW contributed to the reagents, materials, and analysis tools. YiZ, LW, DC, MW, and HZ wrote the manuscript. All authors contributed to the article and approved the submitted version.

## Conflict of Interest

DC, FY, YuZ, CC, and YiZ were employed by the company ABLife Inc. The remaining authors declare that the research was conducted in the absence of any commercial or financial relationships that could be construed as a potential conflict of interest.

## Publisher’s Note

All claims expressed in this article are solely those of the authors and do not necessarily represent those of their affiliated organizations, or those of the publisher, the editors and the reviewers. Any product that may be evaluated in this article, or claim that may be made by its manufacturer, is not guaranteed or endorsed by the publisher.

## Footnotes

1
https://ftp.ncbi.nlm.nih.gov/genomes/all/GCA/000/002/195/GCA_000002195.1_Amel_4.5/

## References

[B1] Altschul S. F. Gish W. Miller W. Myers E. W. Lipman D. J. (1990). Basic local alignment search tool. *J. Mol. Biol.* 215 403–410. 10.1016/s0022-2836(05)80360-2 2231712

[B2] Anders S. Huber W. (2010). Differential expression analysis for sequence count data. *Genome Biol.* 11:R106. 10.1186/gb-2010-11-10-r106 PMC3218662 20979621

[B3] Barber B. A. Rastegar M. (2010). Epigenetic control of Hox genes during neurogenesis, development, and disease. *Ann. Anat.* 192 261–274. 10.1016/j.aanat.2010.07.009 20739155

[B4] Bashirullah A. Halsell S. R. Cooperstock R. L. Kloc M. Karaiskakis A. Fisher W. W. (1999). Joint action of two RNA degradation pathways controls the timing of maternal transcript elimination at the midblastula transition in *Drosophila* melanogaster. *Embo J.* 18 2610–2620. 10.1093/emboj/18.9.2610 10228172 PMC1171340

[B5] Batista P. J. Chang H. Y. (2013). Long noncoding RNAs: cellular address codes in development and disease. *Cell* 152 1298–1307. 10.1016/j.cell.2013.02.012 23498938 PMC3651923

[B6] Bell L. R. Maine E. M. Schedl P. Cline T. W. (1988). Sex-lethal, a *Drosophila* sex determination switch gene, exhibits sex-specific RNA splicing and sequence similarity to RNA binding proteins. *Cell* 55 1037–1046. 10.1016/0092-8674(88)90248-6 3144435

[B7] Bertholf L. M. (1925). The moults of the honeybee. *J. Econ. Entomol.* 18 380–384. 10.1093/jee/18.2.380

[B8] Beye M. Hasselmann M. Fondrk M. K. Page R. E. Omholt S. W. (2003). The gene csd is the primary signal for sexual development in the honeybee and encodes an SR-type protein. *Cell* 114 419–429. 10.1016/s0092-8674(03)00606-8 12941271

[B9] Bopp D. Saccone G. Beye M. (2014). Sex determination in insects: variations on a common theme. *Sex Dev.* 8 20–28. 10.1159/000356458 24335049

[B10] Borensztein M. Syx L. Ancelin K. Diabangouaya P. Picard C. Liu T. (2017). Xist-dependent imprinted X inactivation and the early developmental consequences of its failure. *Nat. Struct. Mol. Biol.* 24 226–233. 10.1038/nsmb.3365 28134930 PMC5337400

[B11] Briggs J. A. Weinreb C. Wagner D. E. Megason S. Peshkin L. Kirschner M. W. (2018). The dynamics of gene expression in vertebrate embryogenesis at single-cell resolution. *Science* 360 eaar5780. 10.1126/science.aar5780 29700227 PMC6038144

[B12] Bull J. J. (1983). *Evolution of Sex Determining Mechanisms.* San Francisco CA: The Benjamin/Cummings Publishing Company, Inc.

[B13] Burton A. Torres-Padilla M. E. (2014). Chromatin dynamics in the regulation of cell fate allocation during early embryogenesis. *Nat. Rev. Mol. Cell Biol.* 15 723–734. 10.1038/nrm3885 25303116

[B14] Bustin S. A. Benes V. Garson J. A. Hellemans J. Huggett J. Kubista M. (2009). The MIQE guidelines: minimum Information for Publication of quantitative real-time PCR experiments. *Clin. Chem.* 55 611–622. 10.1373/clinchem.2008.112797 19246619

[B15] Chu C. Zhang Q. C. Da Rocha S. T. Flynn R. A. Bharadwaj M. Calabrese J. M. (2015). Systematic discovery of Xist RNA binding proteins. *Cell* 161 404–416. 10.1016/j.cell.2015.03.025 25843628 PMC4425988

[B16] da Rocha S. T. Heard E. (2017). Novel players in X inactivation: insights into Xist-mediated gene silencing and chromosome conformation. *Nat. Struct. Mol. Biol.* 24 197–204. 10.1038/nsmb.3370 28257137

[B17] De Iaco A. Planet E. Coluccio A. Verp S. Duc J. Trono D. (2017). DUX-family transcription factors regulate zygotic genome activation in placental mammals. *Nat. Genet.* 49 941–945. 10.1038/ng.3858 28459456 PMC5446900

[B18] Dearden P. K. Wilson M. J. Sablan L. Osborne P. W. Havler M. Mcnaughton E. (2006). Patterns of conservation and change in honey bee developmental genes. *Genome Res.* 16 1376–1384. 10.1101/gr.5108606 17065607 PMC1626639

[B19] Demir E. Dickson B. J. (2005). fruitless splicing specifies male courtship behavior in *Drosophila*. *Cell* 121 785–794. 10.1016/j.cell.2005.04.027 15935764

[B20] Despic V. Neugebauer K. M. (2018). RNA tales – how embryos read and discard messages from mom. *J. Cell Sci.* 131:jcs201996. 10.1242/jcs.201996 29467249

[B21] Devaux Y. Zangrando J. Schroen B. Creemers E. E. Pedrazzini T. Chang C. P. (2015). Long noncoding RNAs in cardiac development and ageing. *Nat. Rev. Cardiol.* 12 415–425. 10.1038/nrcardio.2015.55 25855606

[B22] Fang Y. Feng M. Han B. Qi Y. Hu H. Fan P. (2015). Proteome analysis unravels mechanism underling the embryogenesis of the honeybee drone and its divergence with the worker (*Apis mellifera* lingustica). *J. Proteome Res.* 14 4059–4071. 10.1021/acs.jproteome.5b00625 26260241

[B23] Farrell J. A. Wang Y. Riesenfeld S. J. Shekhar K. Regev A. Schier A. F. (2018). Single-cell reconstruction of developmental trajectories during zebrafish embryogenesis. *Science* 360:eaar3131. 10.1126/science.aar3131 29700225 PMC6247916

[B24] Fatica A. Bozzoni I. (2014). Long non-coding RNAs: new players in cell differentiation and development. *Nat. Rev. Genet.* 15 7–21. 10.1038/nrg3606 24296535

[B25] Fleig R. Sander K. (1985). Blastoderm development in honey bee embryogenesis as seen in the scanning electron microscope. *Int. J. Invertebr. Reprod.* 8 279–286. 10.1080/01688170.1985.10510156

[B26] Fleig R. Sander K. (1986). Embryogenesis of the honeybee *Apis mellifera* l. (hymenoptera : apidae): an sem study. *Int. J. Insect Morphol. Embryol.* 15 449–462. 10.1016/0020-7322(86)90037-1

[B27] Gempe T. Beye M. (2011). Function and evolution of sex determination mechanisms, genes and pathways in insects. *Bioessays* 33 52–60. 10.1002/bies.201000043 21110346 PMC3040292

[B28] Gempe T. Hasselmann M. Schiott M. Hause G. Otte M. Beye M. (2009). Sex determination in honeybees: two separate mechanisms induce and maintain the female pathway. *PLoS Biol.* 7:e1000222. 10.1371/journal.pbio.1000222 19841734 PMC2758576

[B29] Grath S. Parsch J. (2016). Sex-biased gene expression. *Annu. Rev. Genet.* 50 29–44. 27574843 10.1146/annurev-genet-120215-035429

[B30] Hasselmann M. Gempe T. Schiøtt M. Nunes-Silva C. G. Otte M. Beye M. (2008). Evidence for the evolutionary nascence of a novel sex determination pathway in honeybees. *Nature* 454 519–522. 10.1038/nature07052 18594516

[B31] Haussmann I. U. Bodi Z. Sanchez-Moran E. Mongan N. P. Archer N. Fray R. G. (2016). m6A potentiates Sxl alternative pre-mRNA splicing for robust *Drosophila* sex determination. *Nature* 540 301–304. 10.1038/nature20577 27919081

[B32] Hedrick P. W. Parker J. D. (1997). Evolutionary genetics and genetic variation of haplodiploids and X-linked genes. *Annu. Rev. Ecol. Syst.* 28 55–83. 10.1146/annurev.ecolsys.28.1.55

[B33] Honeybee Genome Sequencing. (2006). Insights into social insects from the genome of the honeybee *Apis mellifera*. *Nature* 443 931–949. 10.1038/nature05260 17073008 PMC2048586

[B34] Jeon Y. Lee J. T. (2011). YY1 tethers Xist RNA to the inactive X nucleation center. *Cell* 146 119–133. 10.1016/j.cell.2011.06.026 21729784 PMC3150513

[B35] Kong L. Zhang Y. Ye Z. Q. Liu X. Q. Zhao S. Q. Wei L. (2007). CPC: assess the protein-coding potential of transcripts using sequence features and support vector machine. *Nucleic Acids Res.* 35 W345–W349. 17631615 10.1093/nar/gkm391 PMC1933232

[B36] Langfelder P. Horvath S. (2008). WGCNA: an R package for weighted correlation network analysis. *BMC Bioinformatics* 9:559. 10.1186/1471-2105-9-559 PMC2631488 19114008

[B37] Langley A. R. Smith J. C. Stemple D. L. Harvey S. A. (2014). New insights into the maternal to zygotic transition. *Development* 141 3834–3841. 10.1242/dev.102368 25294937

[B38] Lee M. T. Bonneau A. R. Giraldez A. J. (2014). Zygotic genome activation during the maternal-to-zygotic transition. *Annu. Rev. Cell Dev. Biol.* 30 581–613. 10.1146/annurev-cellbio-100913-013027 25150012 PMC4303375

[B39] Liu S. Wang Z. Chen D. Zhang B. Tian R. Wu J. (2017). Annotation and cluster analysis of spatiotemporal- and sex-related lncRNA expression in Rhesus macaque brain. *Genome Res.* 27 1608–1620. 10.1101/gr.217463.116 28687705 PMC5580719

[B40] Livak K. J. Schmittgen T. D. (2001). Analysis of relative gene expression data using real-time quantitative PCR and the 2(T)(-Delta Delta C) method. *Methods* 25 402–408. 10.1006/meth.2001.1262 11846609

[B41] Lott S. E. Villalta J. E. Schroth G. P. Luo S. Tonkin L. A. Eisen M. B. (2011). Noncanonical compensation of zygotic X transcription in early *Drosophila* melanogaster development revealed through single-embryo RNA-seq. *PLoS Biol.* 9:e1000590. 10.1371/journal.pbio.1000590 21346796 PMC3035605

[B42] Ma S. Dai Y. (2011). Principal component analysis based methods in bioinformatics studies. *Brief Bioinform.* 12 714–722. 10.1093/bib/bbq090 21242203 PMC3220871

[B43] Mallo M. Alonso C. R. (2013). The regulation of Hox gene expression during animal development. *Development* 140 3951–3963. 10.1242/dev.068346 24046316

[B44] Martin M. (2011). Cutadapt removes adapter sequences from high-throughput sequencing reads. *EMBnet J.* 17 10–12. 10.14806/ej.17.1.200

[B45] Mattick J. S. Rinn J. L. (2015). Discovery and annotation of long noncoding RNAs. *Nat. Struct. Mol. Biol.* 22 5–7. 10.1038/nsmb.2942 25565026

[B46] Mattila H. R. Seeley T. D. (2007). Genetic diversity in honey bee colonies enhances productivity and fitness. *Science* 317 362–364. 10.1126/science.1143046 17641199

[B47] McHugh C. A. Chen C. K. Chow A. Surka C. F. Tran C. Mcdonel P. (2015). The Xist lncRNA interacts directly with SHARP to silence transcription through HDAC3. *Nature* 521 232–236. 10.1038/nature14443 25915022 PMC4516396

[B48] Morris K. V. Mattick J. S. (2014). The rise of regulatory RNA. *Nat. Rev. Genet.* 15 423–437. 10.1038/nrg3722 24776770 PMC4314111

[B49] Mortazavi A. Williams B. A. Mccue K. Schaeffer L. Wold B. (2008). Mapping and quantifying mammalian transcriptomes by RNA-Seq. *Nat. Methods* 5 621–628. 10.1038/nmeth.1226 18516045 PMC13303166

[B50] Mulvey B. B. Olcese U. Cabrera J. R. Horabin J. I. (2014). An interactive network of long non-coding RNAs facilitates the *Drosophila* sex determination decision. *Biochim. Biophys. Acta* 1839 773–784. 10.1016/j.bbagrm.2014.06.007 24954180 PMC4134978

[B51] Nelson J. A. (1915). Embryology Of The Honey Bee. Worcestershire: Read Books Ltd press.

[B52] Nissen I. Müller M. Beye M. (2012). The Am-tra2 gene is an essential regulator of female splice regulation at two levels of the sex determination hierarchy of the honeybee. *Genetics* 192 1015–1026. 10.1534/genetics.112.143925 22942126 PMC3522149

[B53] Percharde M. Lin C. J. Yin Y. Guan J. Peixoto G. A. Bulut-Karslioglu A. (2018). A LINE1-nucleolin partnership regulates early development and ESC identity. *Cell* 174:e319. 10.1016/j.cell.2018.05.043 PMC6046266 29937225

[B54] Pires C. V. Freitas F. C. Cristino A. S. Dearden P. K. Simoes Z. L. (2016). Transcriptome analysis of honeybee (*Apis Mellifera*) haploid and diploid embryos reveals early zygotic transcription during cleavage. *PLoS One* 11:e0146447. 10.1371/journal.pone.0146447 26751956 PMC4713447

[B55] Pritchard D. K. Schubiger G. (1996). Activation of transcription in *Drosophila* embryos is a gradual process mediated by the nucleocytoplasmic ratio. *Genes Dev.* 10 1131–1142. 10.1101/gad.10.9.1131 8654928

[B56] Quinn J. J. Chang H. Y. (2016). Unique features of long non-coding RNA biogenesis and function. *Nat. Rev. Genet.* 17 47–62. 10.1038/nrg.2015.10 26666209

[B57] Ratnieks F. L. W. Keller L. (1998). Queen control of egg fertilization in the honey bee. *Behav. Ecol. Sociobiol.* 44 57–61. 10.1007/s002650050514

[B58] Rembold H. Kremer J. P. Ulrich G. M. (1980). Characterization of postembryonic developmental stages of the female castes of the honey bee, *Apis mellifera* L. *Apidologie* 11 29–38. 10.1051/apido:19800104

[B59] Robinson G. E. Grozinger C. M. Whitfield C. W. (2005). Sociogenomics: social life in molecular terms. *Nat. Rev. Genet.* 6 257–270. 10.1038/nrg1575 15761469

[B60] Robinson M. D. Mccarthy D. J. Smyth G. K. (2010). edgeR: a Bioconductor package for differential expression analysis of digital gene expression data. *Bioinformatics* 26 139–140. 10.1093/bioinformatics/btp616 19910308 PMC2796818

[B61] Ryner L. C. Goodwin S. F. Castrillon D. H. Anand A. Villella A. Baker B. S. (1996). Control of male sexual behavior and sexual orientation in *Drosophila* by the fruitless gene. *Cell* 87 1079–1089. 10.1016/s0092-8674(00)81802-4 8978612

[B62] Sahakyan A. Yang Y. Plath K. (2018). The role of xist in X-chromosome dosage compensation. *Trends Cell Biol.* 28 999–1013. 10.1016/j.tcb.2018.05.005 29910081 PMC6249047

[B63] Salz H. K. (2011). Sex determination in insects: a binary decision based on alternative splicing. *Curr. Opin. Genet. Dev.* 21 395–400. 10.1016/j.gde.2011.03.001 21474300 PMC3134629

[B64] Salz H. K. Erickson J. W. (2010). Sex determination in *Drosophila*: the view from the top. *Fly (Austin)* 4 60–70. 10.4161/fly.4.1.11277 20160499 PMC2855772

[B65] Sanchez L. (2008). Sex-determining mechanisms in insects. *Int. J. Dev. Biol.* 52 837–856. 10.1387/ijdb.072396ls 18956315

[B66] Schutt C. Nothiger R. (2000). Structure, function and evolution of sex-determining systems in dipteran insects. *Development* 127 667–677. 10.1242/dev.127.4.667 10648226

[B67] Statello L. Guo C. J. Chen L. L. Huarte M. (2021). Gene regulation by long non-coding RNAs and its biological functions. *Nat. Rev. Mol. Cell Biol.* 22 96–118. 33353982 10.1038/s41580-020-00315-9 PMC7754182

[B68] Sunwoo H. Colognori D. Froberg J. E. Jeon Y. Lee J. T. (2017). Repeat E anchors Xist RNA to the inactive X chromosomal compartment through CDKN1A-interacting protein (CIZ1). *Proc. Natl. Acad. Sci. U.S.A.* 114 10654–10659. 10.1073/pnas.1711206114 28923964 PMC5635913

[B69] Tadano H. Yamazaki Y. Takeuchi H. Kubo T. (2009). Age- and division-of-labour-dependent differential expression of a novel non-coding RNA, Nb-1, in the brain of worker honeybees, *Apis mellifera* L. *Insect Mol. Biol.* 18 715–726. 10.1111/j.1365-2583.2009.00911.x 19817910

[B70] Tadros W. Lipshitz H. D. (2009). The maternal-to-zygotic transition: a play in two acts. *Development* 136 3033–3042. 10.1242/dev.033183 19700615

[B71] Trapnell C. Roberts A. Goff L. Pertea G. Kim D. Kelley D. R. (2012). Differential gene and transcript expression analysis of RNA-seq experiments with TopHat and Cufflinks. *Nat. Protoc.* 7 562–578. 10.1038/nprot.2012.016 22383036 PMC3334321

[B72] Wagner D. E. Weinreb C. Collins Z. M. Briggs J. A. Megason S. G. Klein A. M. (2018). Single-cell mapping of gene expression landscapes and lineage in the zebrafish embryo. *Science* 360 981–987. 10.1126/science.aar4362 29700229 PMC6083445

[B73] Wallberg A. Bunikis I. Pettersson O. V. Mosbech M. B. Childers A. K. Evans J. D. (2019). A hybrid de novo genome assembly of the honeybee, *Apis mellifera*, with chromosome-length scaffolds. *BMC Genomics* 20:275. 10.1186/s12864-019-5642-0 PMC6454739 30961563

[B74] Wallberg A. Han F. Wellhagen G. Dahle B. Kawata M. Haddad N. (2014). A worldwide survey of genome sequence variation provides insight into the evolutionary history of the honeybee *Apis mellifera*. *Nat. Genet.* 46 1081–1088. 10.1038/ng.3077 25151355

[B75] Wang E. T. Sandberg R. Luo S. Khrebtukova I. Zhang L. Mayr C. (2008). Alternative isoform regulation in human tissue transcriptomes. *Nature* 456 470–476. 10.1038/nature07509 18978772 PMC2593745

[B76] Wang F. Shin J. Shea J. M. Yu J. Boskovic A. Byron M. (2016). Regulation of X-linked gene expression during early mouse development by Rlim. *Elife* 5:e19127. 10.7554/eLife.19127 PMC5059138 27642011

[B77] Wang K. Liu F. Zhou L. Y. Long B. Yuan S. M. Wang Y. (2014). The long noncoding RNA CHRF regulates cardiac hypertrophy by targeting miR-489. *Circ. Res.* 114 1377–1388. 10.1161/circresaha.114.302476 24557880

[B78] Wang X. Werren J. H. Clark A. G. (2015). Genetic and epigenetic architecture of sex-biased expression in the jewel wasps *Nasonia vitripennis* and giraulti. *Proc. Natl. Acad. Sci. U.S.A.* 112 E3545–E3554. 26100871 10.1073/pnas.1510338112 PMC4500217

[B79] Wutz A. Rasmussen T. P. Jaenisch R. (2002). Chromosomal silencing and localization are mediated by different domains of Xist RNA. *Nat. Genet.* 30 167–174. 10.1038/ng820 11780141

[B80] Xia H. Chen D. Wu Q. Wu G. Zhou Y. Zhang Y. (2017). CELF1 preferentially binds to exon-intron boundary and regulates alternative splicing in HeLa cells. *Biochim. Biophys. Acta* 1860 911–921. 10.1016/j.bbagrm.2017.07.004 28733224

[B81] Yu F. Y. Zhang Y. Cheng C. Wang W. Q. Zhou Z. S. Rang W. L. (2020). Poly(A)-seq: a method for direct sequencing and analysis of the transcriptomic poly(A)-tails. *PLoS One* 15:e0234696. 10.1371/journal.pone.0234696 32544193 PMC7297374

[B82] Zhang Z. Klein J. Nei M. (2014). Evolution of the sex-lethal gene in insects and origin of the sex-determination system in *Drosophila*. *J. Mol. Evol.* 78 50–65. 10.1007/s00239-013-9599-3 24271947

