# Supplementary material for: Sex-Specific Development in Haplodiploid Honeybee Is Controlled by the Female-Embryo-Specific Activation of Thousands of Intronic LncRNAs
Source: Front Cell Dev Biol. 2021 Aug 6;9:690167. doi: 10.3389/fcell.2021.690167 (PMC8377728; doi:10.3389/fcell.2021.690167)
Supplement: Supplementary file 1 [file Data_Sheet_1.docx]

Supplementary Material

**
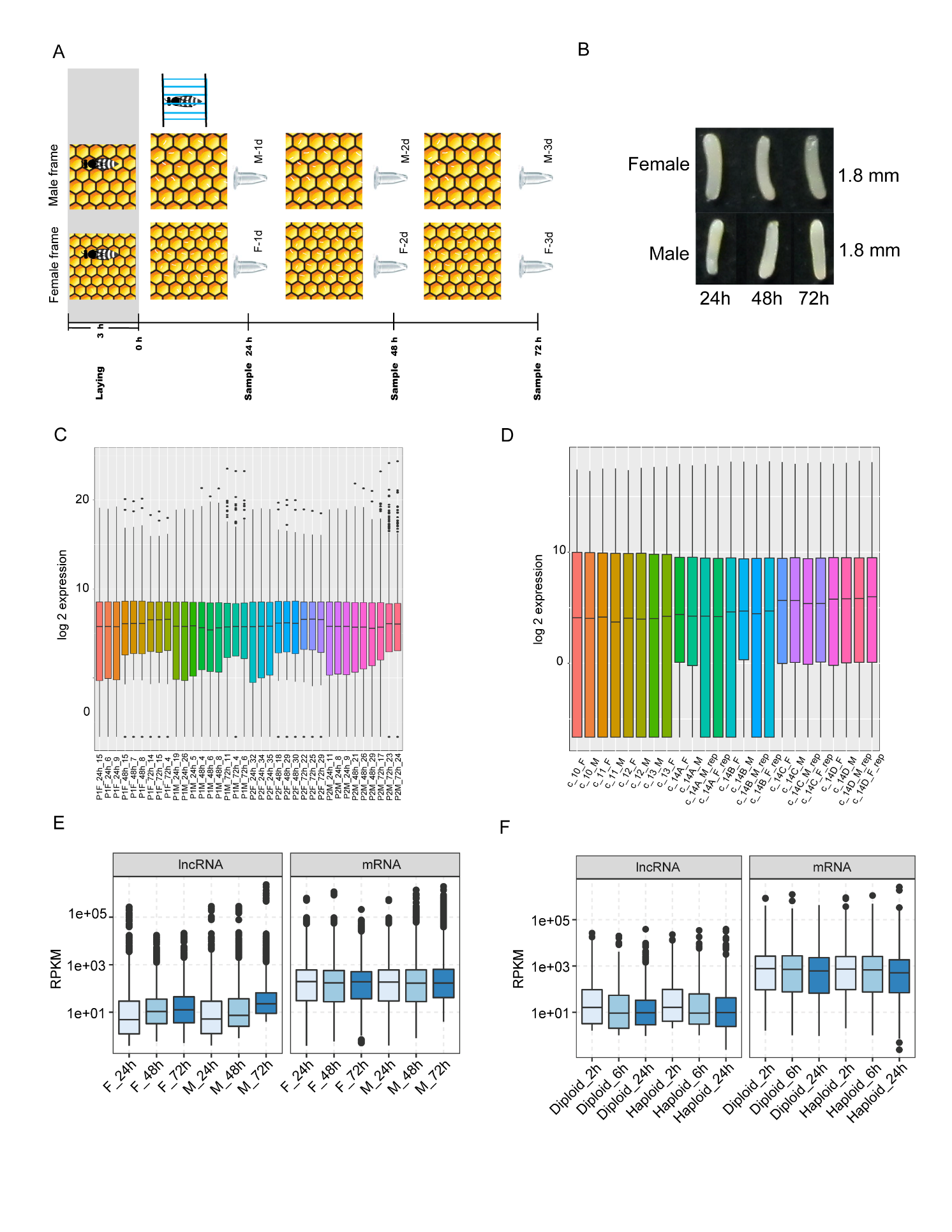
**

**Supplementary Figure 1.** Sample collection procedure and gene expression levels in honey bee embryos from three developmental time points. **(A)** Egg collection procedure. Two different queens were isolated after laying eggs, and we collected eggs from small (female) and big (male) honeycomb at three time points, respectively. **(B)** There was no difference between male and female eggs from the appearance observation. **(C)** Box plot showing the expression level of all expressed genes in 36 single-embryo samples after normalization. **(D)** Boxplot of the normalized expression level of all expressed genes in 24 fruit fly single-embryo samples from Lott et al (Lott et al., 2011). **(E)** Box plot showing the discrepant expression level between annotated lncRNAs and mRNAs in honeybee embryos. **(F)** Box plot showing the more discrepant expression level between lncRNAs and mRNAs from mix embryos of honeybee (Pires et al., 2016) compared with single embryo in (E).


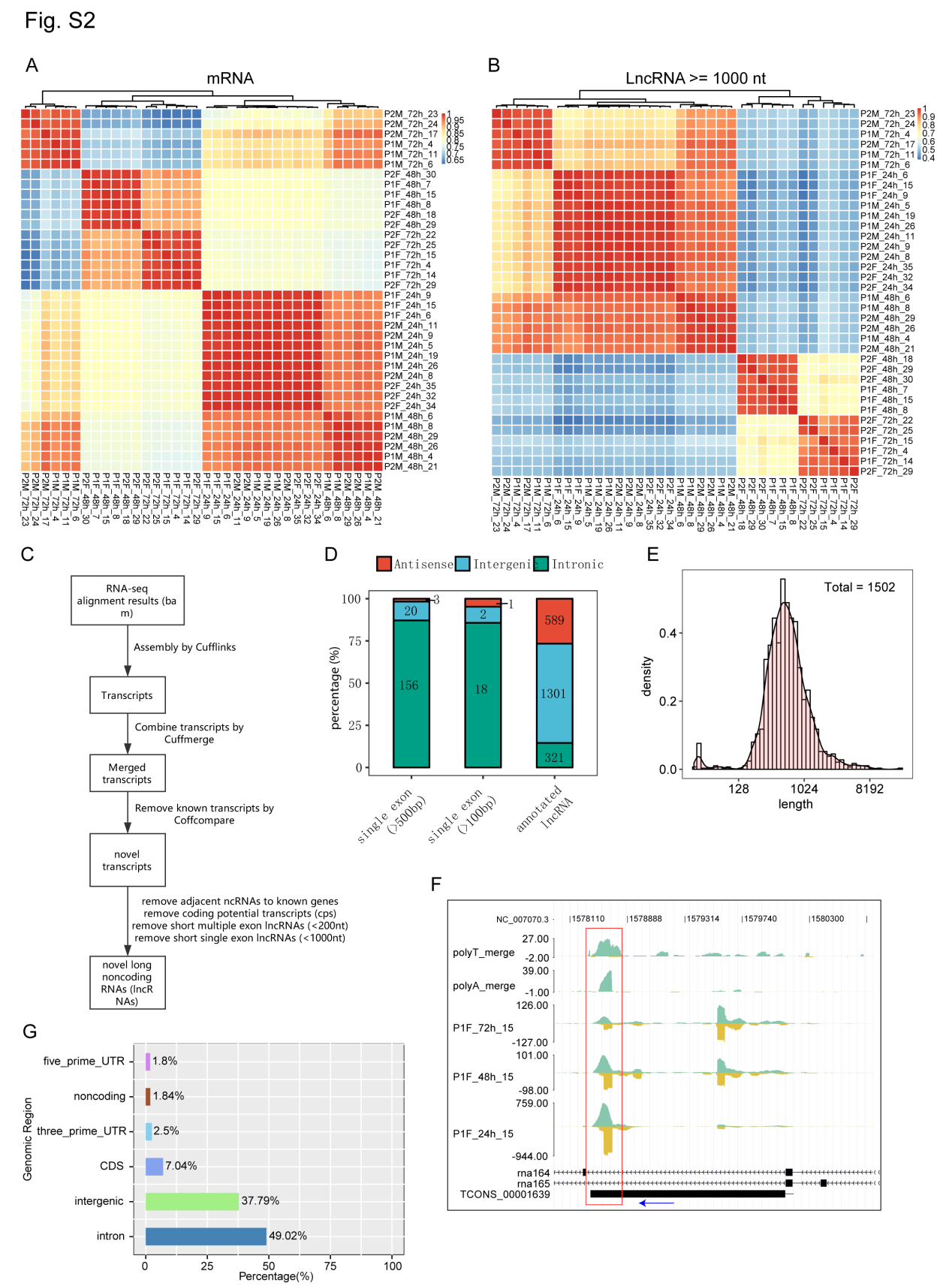


**Supplementary Figure 2.** Embryonic honeybee lncRNAs are dominantly intronic. **(A)** Hierarchical clustering heatmap showing the distribution pattern of sequenced samples. Pearson’s correlation coefficients (PCCs) of expressed mRNA genes for all sample pairs were used as input. The left color bar represents color of each module. **(B)** The same with (A) but for the expressed lncRNAs. **(C)** Analysis pipeline of lncRNAs prediction. For single exon lncRNAs, we set two length thresholds: > 500nt and > 1000nt. **(D)** Bar plot showing the genomic locus of novel and known lncRNAs in fruit fly. LncRNAs were classified as predicted single exonic with two threshold and annotated. **(E)** The length distribution of single exon lncRNAs that were already annotated in fruit fly genome. **(F)** Schematic diagram showing the transcriptional direction identification of single exon lncRNAs with poly (A) tails. The bottom three tracks were reads density in three samples, and the top two tracks were poly (A) or poly (T) (reverse mapped) signals of predicted lncRNAs. The blue arrow was the transcriptional direction of lncRNAs TCONS_00001639. **(G)** The percentage distribution of each genomic regions of honey bee genome sequence.


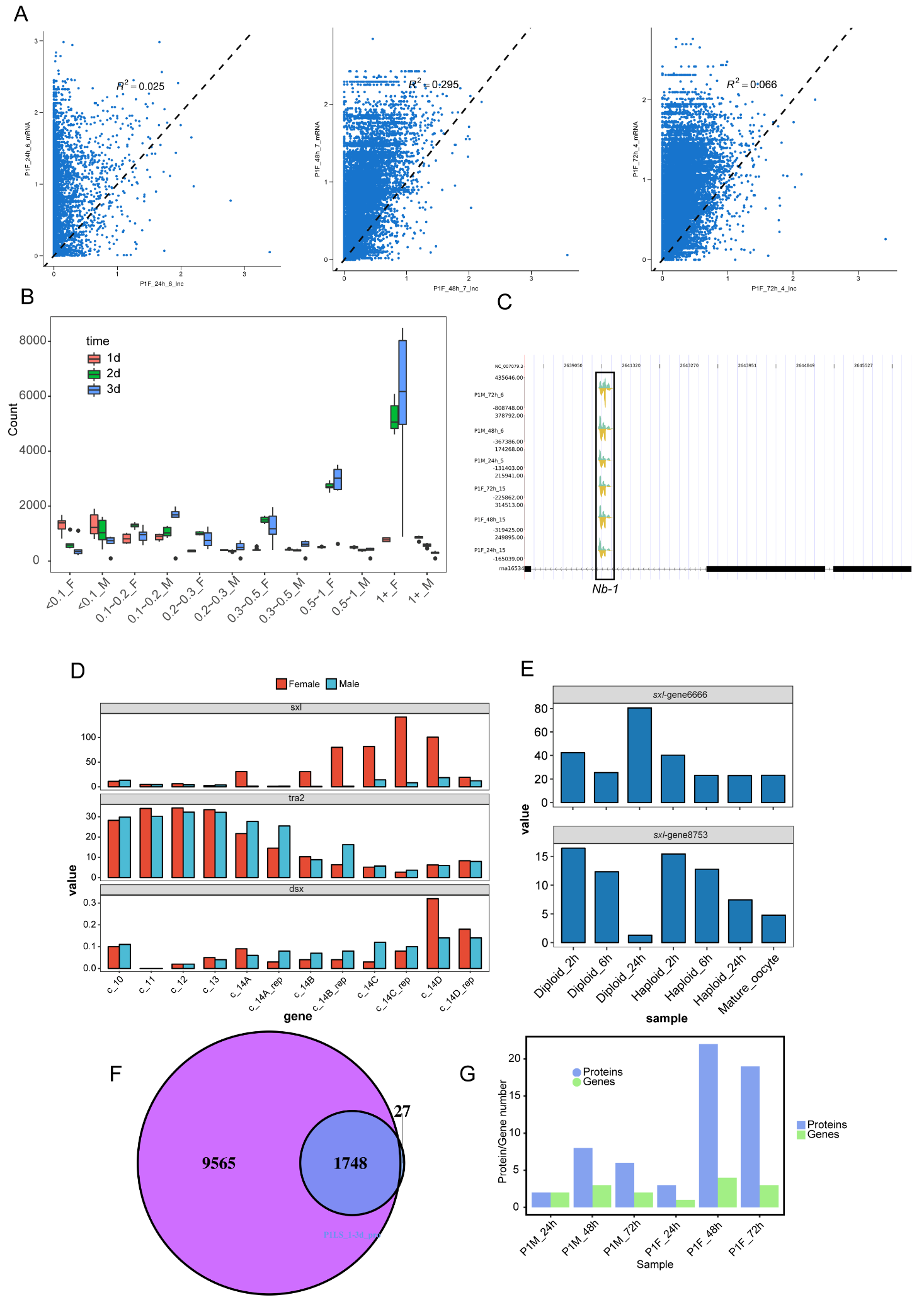


**Supplementary Figure 3.** Expression analysis of lncRNAs and SDGs. (A) Expression correlation analysis showed that intronic lncRNAs was not correlated with their host gene in the aspect of transcription. Expression level of introinc lncRNAs and their host mRNAs in three sequenced samples were shown as examples. X axis represents the lncRNAs expression level, and Y axis represents the mRNAs expression level. **(B)** Box plot showing the number of single-exonic lncRNAs with different expression levels (RPKM value, X-axis) in male (M) and female (F) samples. **(C)** Reads distribution of *Nb-1* and its host lncRNA. Very little transcriptional signal can be observed at the exons (black rectangular frames) of host lncRNAs. **(D)** Expression pattern of *Sxl*, *tra*, and *dsx* during embryogenesis in fruit fly. **(E)** Expression level of two copy of *Sxl* genes in honeybee from the data of Pires et al. **(F)** Venn diagram showing the overlapped genes/proteins between transcriptome and proteome data. **(G)** The detected peptides (proteins) and genes of SDGs from Fang *et al*. One SDG can have more than one peptide.


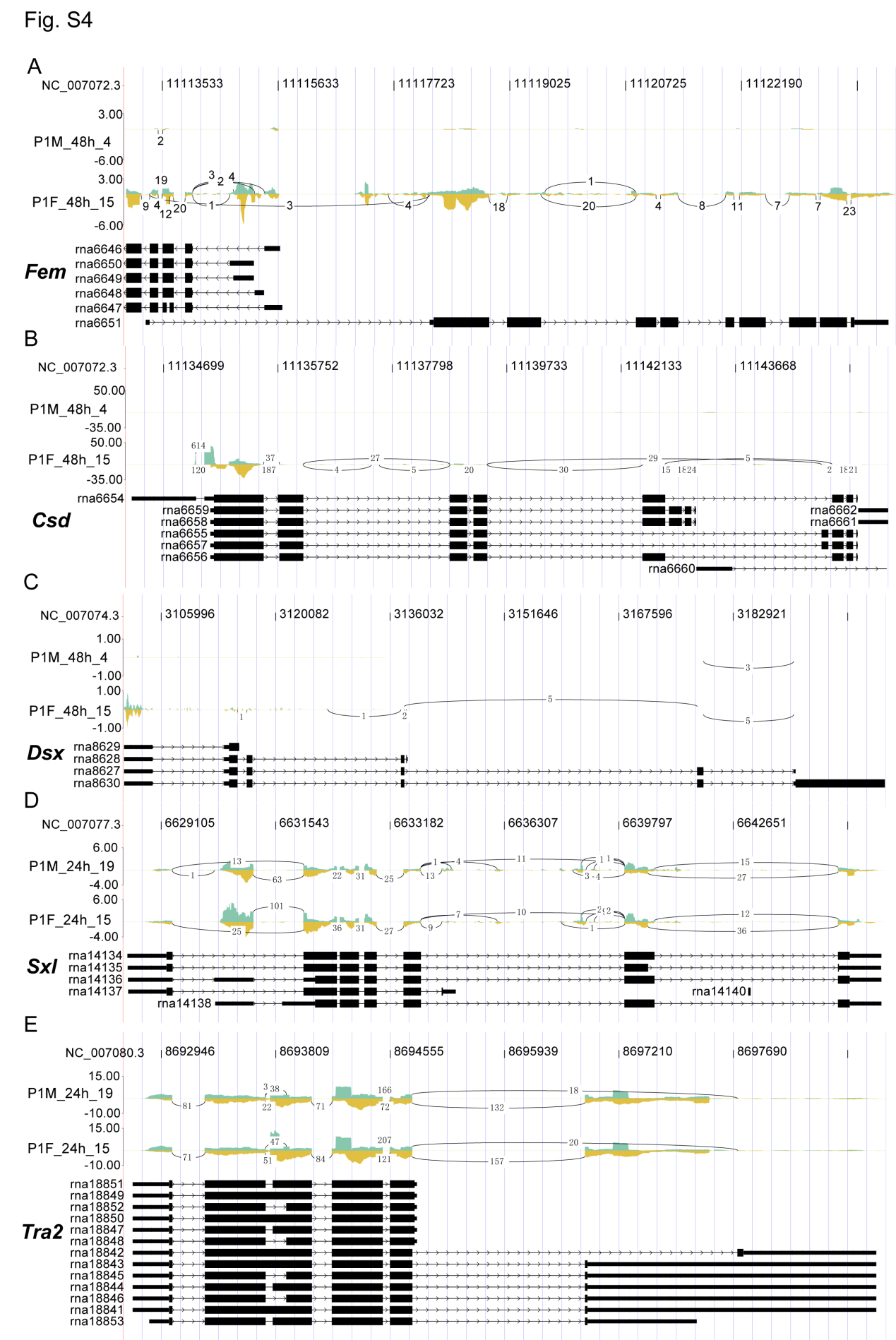


**Supplementary Figure 4.** Plots of the reads density and splicing junction reads from five sex determination genes. **(A)** Reads density and splicing junction reads plot of Fem gene in female and male 48h samples. Splice junction reads were shown in the figure. **(B)** The same with (A) but for the *Csd* gene. **(C)** The same with (A) but for the *Dsx* gene. **(D)** The same with (A) but for the *Sxl* gene. **(E)** The same with (A) but for the *Tra2* gene.

**Table S1.** The RNA-seq sequencing information of this study. “Filtered reads” represent the reads after quality filtering. “uniqtag” represents the reads number after removing the PCR duplicates.

| **Sample** | **Raw reads** | **Filtered reads** | **Filtered Per** | **uniqtag** |
| --- | --- | --- | --- | --- |
| P1F_24h_15 | 34000354 | 27366010 | 80.49% | 13816582(50.49%) |
| P1F_24h_6 | 29104840 | 23331820 | 80.16% | 11832435(50.71%) |
| P1F_24h_9 | 30254568 | 24852285 | 82.14% | 12106755(48.71%) |
| P1F_48h_15 | 32669406 | 23886303 | 73.12% | 13052361(54.64%) |
| P1F_48h_7 | 23604592 | 17402043 | 73.72% | 10262547(58.97%) |
| P1F_48h_8 | 25513010 | 18696177 | 73.28% | 10695796(57.21%) |
| P1F_72h_14 | 23600308 | 17727678 | 75.12% | 11103513(62.63%) |
| P1F_72h_15 | 24055672 | 17781856 | 73.92% | 10543225(59.29%) |
| P1F_72h_4 | 24177076 | 18078024 | 74.77% | 11001614(60.86%) |
| P1M_24h_19 | 32934318 | 26287516 | 79.82% | 13175108(50.12%) |
| P1M_24h_26 | 35032484 | 28241551 | 80.62% | 13750786(48.69%) |
| P1M_24h_5 | 28629002 | 21322198 | 74.48% | 11820655(55.44%) |
| P1M_48h_4 | 22241188 | 14410712 | 64.79% | 7124149(49.44%) |
| P1M_48h_6 | 30735866 | 22248839 | 72.39% | 10799701(48.54%) |
| P1M_48h_8 | 25619582 | 17272972 | 67.42% | 8365267(48.43%) |
| P1M_72h_11 | 26506578 | 15726159 | 59.33% | 5529666(35.16%) |
| P1M_72h_4 | 23122172 | 13421286 | 58.05% | 4915721(36.63%) |
| P1M_72h_6 | 24570452 | 15124585 | 61.56% | 5576823(36.87%) |
| P2F_24h_32 | 21892076 | 16558735 | 75.64% | 9600429(57.98%) |
| P2F_24h_34 | 29407456 | 22321578 | 75.90% | 12485844(55.94%) |
| P2F_24h_35 | 27253668 | 20085787 | 73.70% | 11591218(57.71%) |
| P2F_48h_18 | 25330610 | 18437947 | 72.79% | 11200267(60.75%) |
| P2F_48h_29 | 27246866 | 19623470 | 72.02% | 11150187(56.82%) |
| P2F_48h_30 | 26813650 | 19446280 | 72.52% | 11134670(57.26%) |
| P2F_72h_22 | 24809006 | 18064035 | 72.81% | 11141254(61.68%) |
| P2F_72h_25 | 31568034 | 23235697 | 73.61% | 13912662(59.88%) |
| P2F_72h_29 | 26553300 | 20000856 | 75.32% | 12058565(60.29%) |
| P2M_24h_11 | 24892022 | 18457202 | 74.15% | 10677608(57.85%) |
| P2M_24h_8 | 24441350 | 17821813 | 72.92% | 10094477(56.64%) |
| P2M_24h_9 | 25337762 | 18962862 | 74.84% | 10824603(57.08%) |
| P2M_48h_21 | 29082270 | 19437083 | 66.83% | 8699452(44.76%) |
| P2M_48h_26 | 38099440 | 25183283 | 66.10% | 11842846(47.03%) |
| P2M_48h_29 | 27393514 | 18669475 | 68.15% | 8940074(47.89%) |
| P2M_72h_17 | 25429386 | 15723154 | 61.83% | 5900000(37.52%) |
| P2M_72h_23 | 25027476 | 15451781 | 61.74% | 5112232(33.09%) |
| P2M_72h_24 | 23438910 | 14266115 | 60.87% | 4625886(32.43%) |

**Table S2** Reads distribution across the genomic regions. “Nc_exon” represents the noncoding exons belonging to noncoding genes (miRNA, lncRNA, snRNA, snoRNA and so on).

| **Sample** | **5'UTR** | **3'UTR** | **CDS** | **Nc_exon** | **Introns** | **Intergenic** |
| --- | --- | --- | --- | --- | --- | --- |
| P1F_24h_15 | 3149940(14.35%) | 2758856(12.57%) | 11305123(51.50%) | 1187749(5.41%) | 2060050(9.38%) | 1490511(6.79%) |
| P1F_24h_6 | 2708707(14.35%) | 2352723(12.47%) | 9387961(49.75%) | 1234542(6.54%) | 1875688(9.94%) | 1312406(6.95%) |
| P1F_24h_9 | 2693576(13.29%) | 2746632(13.55%) | 10533142(51.95%) | 1180182(5.82%) | 1755038(8.66%) | 1366546(6.74%) |
| P1F_48h_15 | 1672758(8.87%) | 2824894(14.97%) | 7314135(38.77%) | 1377589(7.30%) | 4057232(21.51%) | 1617579(8.57%) |
| P1F_48h_7 | 1254407(9.26%) | 1930912(14.25%) | 5042949(37.21%) | 948309(7.00%) | 3149576(23.24%) | 1225233(9.04%) |
| P1F_48h_8 | 1162078(8.04%) | 2256462(15.61%) | 5332222(36.90%) | 1145421(7.93%) | 3286295(22.74%) | 1269355(8.78%) |
| P1F_72h_14 | 1300485(9.56%) | 1636918(12.03%) | 5878376(43.21%) | 881450(6.48%) | 2527443(18.58%) | 1379673(10.14%) |
| P1F_72h_15 | 1249232(9.14%) | 1728526(12.65%) | 5931892(43.40%) | 981835(7.18%) | 2492727(18.24%) | 1283671(9.39%) |
| P1F_72h_4 | 1487359(10.46%) | 1709100(12.02%) | 6227071(43.81%) | 786485(5.53%) | 2609332(18.36%) | 1395058(9.81%) |
| P1M_24h_19 | 2499180(11.64%) | 3448070(16.06%) | 9836764(45.82%) | 1686778(7.86%) | 2248093(10.47%) | 1750016(8.15%) |
| P1M_24h_26 | 2806204(12.15%) | 3556212(15.39%) | 10728530(46.43%) | 1820225(7.88%) | 2347605(10.16%) | 1846573(7.99%) |
| P1M_24h_5 | 1869913(10.75%) | 3086277(17.74%) | 7512482(43.18%) | 1511584(8.69%) | 1893140(10.88%) | 1523825(8.76%) |
| P1M_48h_4 | 787033(7.74%) | 1398124(13.74%) | 2697331(26.51%) | 2009701(19.75%) | 2403196(23.62%) | 878588(8.64%) |
| P1M_48h_6 | 1919958(11.06%) | 2383688(13.73%) | 7028630(40.49%) | 1802931(10.39%) | 3177847(18.31%) | 1044500(6.02%) |
| P1M_48h_8 | 1001806(8.07%) | 1702341(13.71%) | 3470623(27.95%) | 2257909(18.18%) | 3084293(24.84%) | 900703(7.25%) |
| P1M_72h_11 | 371365(3.57%) | 714781(6.87%) | 1687110(16.23%) | 3076848(29.59%) | 3557023(34.21%) | 990907(9.53%) |
| P1M_72h_4 | 313359(3.50%) | 721400(8.05%) | 1386611(15.47%) | 2380413(26.56%) | 3420347(38.16%) | 741496(8.27%) |
| P1M_72h_6 | 443174(4.30%) | 803077(7.80%) | 1729342(16.79%) | 2834871(27.52%) | 3612206(35.07%) | 877573(8.52%) |
| P2F_24h_32 | 1749517(13.06%) | 1973388(14.73%) | 6163549(46.00%) | 1073181(8.01%) | 1383550(10.33%) | 1055284(7.88%) |
| P2F_24h_34 | 2459275(13.57%) | 2462898(13.59%) | 8759410(48.34%) | 1349560(7.45%) | 1741661(9.61%) | 1348307(7.44%) |
| P2F_24h_35 | 2066128(13.07%) | 2247254(14.22%) | 7091240(44.86%) | 1378737(8.72%) | 1783982(11.28%) | 1241515(7.85%) |
| P2F_48h_18 | 1058551(7.43%) | 2215472(15.54%) | 5190168(36.41%) | 1073588(7.53%) | 3323920(23.32%) | 1391277(9.76%) |
| P2F_48h_29 | 1126707(7.39%) | 2402093(15.76%) | 5111052(33.54%) | 1336659(8.77%) | 3800658(24.94%) | 1460044(9.58%) |
| P2F_48h_30 | 1333359(8.78%) | 2261379(14.89%) | 5972206(39.31%) | 1080810(7.11%) | 3225446(21.23%) | 1319099(8.68%) |
| P2F_72h_22 | 1072476(7.68%) | 2043600(14.64%) | 5134080(36.77%) | 1078535(7.72%) | 2990614(21.42%) | 1644468(11.78%) |
| P2F_72h_25 | 1411290(7.85%) | 2511022(13.97%) | 6878237(38.28%) | 1344143(7.48%) | 3748192(20.86%) | 2075999(11.55%) |
| P2F_72h_29 | 1338086(8.62%) | 2016301(12.99%) | 6660749(42.91%) | 1052917(6.78%) | 2853722(18.38%) | 1601402(10.32%) |
| P2M_24h_11 | 1722806(11.50%) | 2397796(16.01%) | 6853998(45.76%) | 1230391(8.22%) | 1510555(10.09%) | 1261446(8.42%) |
| P2M_24h_8 | 1925936(13.32%) | 2160850(14.94%) | 6327372(43.75%) | 1363865(9.43%) | 1476198(10.21%) | 1207817(8.35%) |
| P2M_24h_9 | 1807009(11.72%) | 2439246(15.82%) | 6743503(43.73%) | 1430533(9.28%) | 1677314(10.88%) | 1323202(8.58%) |
| P2M_48h_21 | 912439(6.63%) | 1819030(13.21%) | 3461867(25.15%) | 3239066(23.53%) | 3197483(23.23%) | 1135858(8.25%) |
| P2M_48h_26 | 1499300(8.12%) | 2858891(15.48%) | 4989401(27.01%) | 3611058(19.55%) | 4078944(22.08%) | 1432590(7.76%) |
| P2M_48h_29 | 1327475(9.49%) | 1898059(13.57%) | 4252461(30.41%) | 2288440(16.37%) | 3301999(23.61%) | 915264(6.55%) |
| P2M_72h_17 | 498662(4.64%) | 901696(8.38%) | 1801972(16.75%) | 3032826(28.19%) | 3768148(35.03%) | 754738(7.02%) |
| P2M_72h_23 | 242311(2.34%) | 608446(5.87%) | 1317895(12.70%) | 3126957(30.14%) | 4361022(42.04%) | 716552(6.91%) |
| P2M_72h_24 | 226682(2.38%) | 512274(5.38%) | 1301644(13.67%) | 3238149(34.02%) | 3384347(35.55%) | 855820(8.99%) |

**Table S3.** Identification of lncRNA transcriptional direction by poly (A) signal. This list provided lncRNAs with determinate directions.

| **lncRNA gene** | **left_A** | **right_A** | **left_T** | **right_T** | **polyA_strand** | **type** |
| --- | --- | --- | --- | --- | --- | --- |
| XLOC_044968 | 0 | 0 | 12 | 0 | - | singleexon |
| XLOC_009473 | 1 | 0 | 3 | 0 | - | singleexon |
| XLOC_036102 | 0 | 0 | 11 | 0 | - | singleexon |
| XLOC_055994 | 1 | 0 | 3 | 0 | - | singleexon |
| XLOC_025899 | 0 | 0 | 14 | 0 | - | singleexon |
| XLOC_045056 | 0 | 0 | 5 | 0 | - | singleexon |
| XLOC_038253 | 0 | 0 | 38 | 0 | - | singleexon |
| XLOC_004309 | 0 | 0 | 1 | 5 | + | multiexon |
| XLOC_025027 | 0 | 0 | 4 | 0 | - | singleexon |
| XLOC_006884 | 1 | 0 | 8 | 0 | - | singleexon |
| XLOC_013745 | 0 | 0 | 8 | 0 | - | singleexon |
| XLOC_000458 | 1 | 0 | 4 | 1 | - | singleexon |
| XLOC_054435 | 0 | 0 | 15 | 2 | - | singleexon |
| XLOC_059729 | 0 | 0 | 0 | 10 | + | singleexon |
| XLOC_062052 | 1 | 0 | 1 | 3 | + | singleexon |
| XLOC_005074 | 0 | 0 | 4 | 1 | - | multiexon |
| XLOC_034925 | 0 | 0 | 0 | 5 | + | singleexon |
| XLOC_000851 | 3 | 0 | 1 | 3 | - | singleexon |
| XLOC_031502 | 0 | 0 | 0 | 3 | + | multiexon |
| XLOC_056234 | 0 | 0 | 1 | 5 | + | multiexon |
| XLOC_002209 | 0 | 0 | 0 | 11 | + | multiexon |
| XLOC_020136 | 0 | 3 | 0 | 1 | + | singleexon |
| XLOC_059306 | 0 | 0 | 5 | 0 | - | singleexon |
| XLOC_017527 | 0 | 1 | 2 | 2 | - | singleexon |
| XLOC_038192 | 3 | 0 | 0 | 0 | - | singleexon |
| XLOC_054572 | 0 | 0 | 26 | 0 | - | singleexon |
| XLOC_032593 | 0 | 0 | 11 | 0 | - | singleexon |
| XLOC_040111 | 0 | 0 | 1 | 3 | + | singleexon |
| XLOC_026867 | 5 | 0 | 0 | 0 | - | singleexon |
| XLOC_018348 | 0 | 0 | 0 | 3 | + | singleexon |
| XLOC_009780 | 0 | 0 | 3 | 1 | - | singleexon |
| XLOC_059407 | 0 | 0 | 3 | 1 | - | singleexon |
| XLOC_029593 | 0 | 0 | 0 | 5 | + | singleexon |
| XLOC_016008 | 4 | 0 | 2 | 0 | - | singleexon |
| XLOC_028003 | 0 | 0 | 2 | 3 | + | singleexon |
| XLOC_014063 | 0 | 0 | 0 | 5 | + | singleexon |
| XLOC_032296 | 1 | 0 | 5 | 2 | - | singleexon |
| XLOC_039254 | 0 | 1 | 0 | 2 | + | singleexon |
| XLOC_030473 | 0 | 0 | 0 | 6 | + | singleexon |
| XLOC_001311 | 0 | 0 | 0 | 5 | + | singleexon |
| XLOC_050623 | 0 | 0 | 0 | 7 | + | singleexon |
| XLOC_021629 | 0 | 0 | 0 | 8 | + | multiexon |
| XLOC_053057 | 1 | 0 | 2 | 0 | - | singleexon |
| XLOC_050280 | 0 | 1 | 0 | 2 | + | singleexon |
| XLOC_016704 | 2 | 0 | 1 | 0 | - | singleexon |
| XLOC_050055 | 1 | 0 | 15 | 0 | - | singleexon |
| XLOC_000895 | 0 | 0 | 10 | 1 | - | singleexon |
| XLOC_003641 | 0 | 0 | 18 | 0 | - | singleexon |
| XLOC_000185 | 0 | 2 | 1 | 3 | + | singleexon |
| XLOC_016162 | 0 | 0 | 27 | 4 | - | multiexon |
| XLOC_025268 | 0 | 0 | 0 | 16 | + | singleexon |
| XLOC_012733 | 1 | 0 | 2 | 0 | - | singleexon |
| XLOC_022734 | 1 | 0 | 0 | 9 | + | singleexon |
| XLOC_061719 | 0 | 0 | 0 | 11 | + | singleexon |
| XLOC_010279 | 0 | 0 | 0 | 12 | + | singleexon |
| XLOC_056288 | 0 | 59 | 0 | 9 | + | multiexon |
| XLOC_039888 | 0 | 0 | 3 | 1 | - | singleexon |
| XLOC_012048 | 0 | 0 | 0 | 8 | + | singleexon |
| XLOC_003986 | 1 | 0 | 4 | 1 | - | singleexon |
| XLOC_023151 | 0 | 0 | 0 | 3 | + | singleexon |
| XLOC_010178 | 1 | 0 | 3 | 1 | - | singleexon |
| XLOC_018397 | 0 | 1 | 2 | 3 | + | singleexon |
| XLOC_034240 | 0 | 0 | 1 | 8 | + | singleexon |
| XLOC_022430 | 0 | 0 | 44 | 1 | - | multiexon |
| XLOC_047476 | 2 | 0 | 31 | 1 | - | multiexon |
| XLOC_004395 | 0 | 0 | 3 | 0 | - | multiexon |
| XLOC_021865 | 0 | 0 | 0 | 6 | + | multiexon |
| XLOC_037346 | 1 | 0 | 9 | 0 | - | singleexon |
| XLOC_050160 | 0 | 0 | 8 | 0 | - | singleexon |
| XLOC_037280 | 0 | 0 | 4 | 0 | - | singleexon |
| XLOC_020893 | 0 | 0 | 0 | 40 | + | singleexon |
| XLOC_036813 | 0 | 0 | 7 | 19 | + | singleexon |
| XLOC_022699 | 0 | 0 | 0 | 6 | + | singleexon |
| XLOC_012664 | 0 | 0 | 5 | 0 | - | singleexon |
| XLOC_045264 | 0 | 0 | 34 | 0 | - | singleexon |
| XLOC_041649 | 0 | 0 | 4 | 0 | - | multiexon |
| XLOC_050661 | 0 | 0 | 0 | 9 | + | singleexon |
| XLOC_064508 | 0 | 0 | 0 | 3 | + | singleexon |
| XLOC_062848 | 0 | 0 | 0 | 8 | + | singleexon |
| XLOC_054562 | 0 | 0 | 32 | 0 | - | singleexon |
| XLOC_042837 | 0 | 0 | 0 | 6 | + | singleexon |
| XLOC_021848 | 0 | 1 | 4 | 3 | - | singleexon |
| XLOC_003739 | 0 | 0 | 0 | 4 | + | singleexon |
| XLOC_044663 | 1 | 1 | 0 | 7 | + | singleexon |
| XLOC_005000 | 0 | 0 | 7 | 1 | - | singleexon |
| XLOC_058532 | 1 | 3 | 6 | 184 | + | multiexon |
| XLOC_056045 | 0 | 0 | 10 | 0 | - | singleexon |
| XLOC_001403 | 0 | 2 | 0 | 16 | + | singleexon |
| XLOC_035369 | 0 | 0 | 0 | 4 | + | singleexon |
| XLOC_013506 | 0 | 1 | 3 | 0 | - | singleexon |
| XLOC_059941 | 0 | 0 | 0 | 103 | + | singleexon |
| XLOC_015718 | 0 | 1 | 0 | 12 | + | singleexon |
| XLOC_018975 | 0 | 0 | 0 | 3 | + | singleexon |
| XLOC_061870 | 1 | 0 | 6 | 0 | - | singleexon |
| XLOC_007947 | 0 | 0 | 3 | 0 | - | multiexon |
| XLOC_016268 | 0 | 0 | 0 | 20 | + | singleexon |
| XLOC_037394 | 0 | 0 | 29 | 1 | - | singleexon |
| XLOC_029252 | 0 | 1 | 1 | 5 | + | singleexon |
| XLOC_031021 | 0 | 0 | 5 | 0 | - | singleexon |
| XLOC_012002 | 0 | 1 | 10 | 6 | - | singleexon |
| XLOC_008335 | 2 | 0 | 16 | 0 | - | singleexon |
| XLOC_034939 | 0 | 2 | 0 | 12 | + | singleexon |
| XLOC_031124 | 0 | 0 | 9 | 0 | - | singleexon |
| XLOC_060027 | 0 | 0 | 78 | 0 | - | multiexon |
| XLOC_037444 | 1 | 0 | 3 | 1 | - | singleexon |
| XLOC_024875 | 0 | 0 | 5 | 0 | - | singleexon |
| XLOC_034162 | 0 | 0 | 0 | 26 | + | multiexon |
| XLOC_042762 | 0 | 1 | 8 | 0 | - | singleexon |
| XLOC_056743 | 0 | 0 | 9 | 2 | - | singleexon |
| XLOC_054836 | 0 | 9 | 0 | 0 | + | singleexon |
| XLOC_021564 | 0 | 2 | 0 | 1 | + | multiexon |
| XLOC_022502 | 0 | 0 | 3 | 0 | - | singleexon |
| XLOC_022213 | 0 | 0 | 3 | 0 | - | singleexon |
| XLOC_060703 | 0 | 0 | 45 | 0 | - | singleexon |
| XLOC_015660 | 0 | 0 | 3 | 1 | - | singleexon |
| XLOC_045121 | 0 | 0 | 5 | 0 | - | singleexon |
| XLOC_022180 | 0 | 1 | 1 | 2 | + | singleexon |
| XLOC_017314 | 3 | 0 | 0 | 0 | - | singleexon |
| XLOC_051929 | 1 | 0 | 5 | 0 | - | singleexon |
| XLOC_053214 | 0 | 0 | 0 | 10 | + | multiexon |
| XLOC_010822 | 0 | 0 | 10 | 1 | - | singleexon |
| XLOC_053420 | 0 | 0 | 9 | 0 | - | singleexon |
| XLOC_024887 | 0 | 3 | 0 | 0 | + | singleexon |
| XLOC_003616 | 0 | 0 | 0 | 4 | + | singleexon |
| XLOC_029268 | 0 | 0 | 0 | 3 | + | singleexon |
| XLOC_027293 | 0 | 1 | 0 | 4 | + | singleexon |
| XLOC_040356 | 0 | 1 | 6 | 4 | - | singleexon |
| XLOC_024981 | 0 | 0 | 3 | 0 | - | singleexon |
| XLOC_000395 | 0 | 3 | 7 | 1 | - | singleexon |
| XLOC_002827 | 0 | 0 | 7 | 0 | - | singleexon |
| XLOC_018953 | 0 | 0 | 0 | 3 | + | singleexon |
| XLOC_003664 | 0 | 0 | 6 | 0 | - | singleexon |
| XLOC_004847 | 0 | 0 | 4 | 1 | - | singleexon |
| XLOC_012267 | 0 | 2 | 1 | 2 | + | multiexon |
| XLOC_044048 | 0 | 3 | 1 | 2 | + | singleexon |
| XLOC_018566 | 0 | 0 | 0 | 7 | + | singleexon |
| XLOC_040643 | 0 | 0 | 5 | 1 | - | singleexon |
| XLOC_025620 | 0 | 0 | 9 | 3 | - | singleexon |
| XLOC_052097 | 0 | 0 | 0 | 16 | + | singleexon |
| XLOC_025114 | 1 | 0 | 2 | 0 | - | singleexon |
| XLOC_029448 | 1 | 0 | 0 | 91 | + | singleexon |
| XLOC_013587 | 0 | 1 | 0 | 6 | + | singleexon |
| XLOC_024525 | 0 | 1 | 0 | 5 | + | multiexon |
| XLOC_005228 | 0 | 0 | 4 | 0 | - | singleexon |
| XLOC_039892 | 1 | 0 | 4 | 0 | - | singleexon |
| XLOC_005090 | 0 | 0 | 3 | 0 | - | multiexon |
| XLOC_057447 | 1 | 2 | 1 | 13 | + | singleexon |
| XLOC_031160 | 0 | 1 | 0 | 2 | + | singleexon |
| XLOC_057642 | 0 | 0 | 3 | 0 | - | singleexon |
| XLOC_062620 | 1 | 0 | 10 | 3 | - | singleexon |
| XLOC_058290 | 0 | 0 | 0 | 3 | + | singleexon |
| XLOC_053400 | 0 | 0 | 3 | 9 | + | singleexon |
| XLOC_029219 | 0 | 0 | 1 | 4 | + | singleexon |
| XLOC_037387 | 0 | 0 | 6 | 0 | - | singleexon |
| XLOC_012642 | 0 | 0 | 0 | 4 | + | singleexon |
| XLOC_028004 | 0 | 0 | 0 | 5 | + | singleexon |
| XLOC_059822 | 0 | 0 | 2 | 5 | + | singleexon |
| XLOC_012940 | 0 | 0 | 3 | 0 | - | multiexon |
| XLOC_054788 | 0 | 1 | 0 | 27 | + | singleexon |
| XLOC_044517 | 0 | 0 | 3 | 3 | - | singleexon |
| XLOC_037516 | 0 | 1 | 0 | 12 | + | singleexon |
| XLOC_052807 | 0 | 0 | 0 | 4 | + | singleexon |
| XLOC_006925 | 0 | 1 | 0 | 3 | + | singleexon |
| XLOC_000636 | 67 | 0 | 404 | 0 | - | multiexon |
| XLOC_013293 | 0 | 0 | 0 | 3 | + | singleexon |
| XLOC_006952 | 0 | 0 | 0 | 4 | + | singleexon |
| XLOC_056957 | 2 | 0 | 2 | 2 | - | multiexon |
| XLOC_059302 | 1 | 0 | 2 | 0 | - | singleexon |
| XLOC_057557 | 6 | 0 | 0 | 0 | - | multiexon |
| XLOC_006934 | 0 | 1 | 0 | 2 | + | singleexon |
| XLOC_062300 | 0 | 0 | 0 | 8 | + | multiexon |
| XLOC_036875 | 0 | 0 | 3 | 3 | - | singleexon |
| XLOC_043889 | 0 | 1 | 0 | 45 | + | multiexon |
| XLOC_038969 | 0 | 0 | 5 | 0 | - | singleexon |
| XLOC_039191 | 0 | 0 | 1 | 4 | + | singleexon |
| XLOC_059267 | 0 | 0 | 5 | 0 | - | singleexon |
| XLOC_038236 | 1 | 0 | 3 | 0 | - | singleexon |
| XLOC_053729 | 0 | 0 | 35 | 0 | - | singleexon |
| XLOC_041778 | 0 | 1 | 0 | 7 | + | multiexon |
| XLOC_046885 | 0 | 0 | 12 | 0 | - | singleexon |
| XLOC_003643 | 0 | 0 | 1 | 3 | + | singleexon |
| XLOC_053081 | 0 | 0 | 2 | 3 | + | singleexon |
| XLOC_013255 | 0 | 0 | 21 | 0 | - | singleexon |
| XLOC_046523 | 0 | 0 | 0 | 4 | + | singleexon |
| XLOC_028258 | 0 | 0 | 0 | 3 | + | singleexon |
| XLOC_034290 | 1 | 0 | 10 | 1 | - | singleexon |
| XLOC_045265 | 1 | 0 | 59 | 0 | - | singleexon |
| XLOC_048567 | 0 | 10 | 1 | 9 | + | multiexon |
| XLOC_053307 | 0 | 0 | 3 | 4 | + | singleexon |
| XLOC_060809 | 0 | 0 | 0 | 4 | + | singleexon |
| XLOC_022703 | 0 | 0 | 2 | 6 | + | singleexon |
| XLOC_015773 | 1 | 0 | 0 | 8 | + | singleexon |
| XLOC_001534 | 0 | 0 | 0 | 4 | + | singleexon |
| XLOC_040065 | 0 | 0 | 1 | 15 | + | singleexon |
| XLOC_000839 | 0 | 0 | 0 | 6 | + | multiexon |
| XLOC_026854 | 0 | 1 | 0 | 2 | + | singleexon |
| XLOC_003835 | 0 | 0 | 7 | 1 | - | singleexon |
| XLOC_024600 | 2 | 3 | 0 | 54 | + | singleexon |
| XLOC_057933 | 0 | 0 | 3 | 1 | - | singleexon |
| XLOC_060444 | 0 | 0 | 3 | 0 | - | multiexon |
| XLOC_035950 | 4 | 0 | 1 | 0 | - | singleexon |
| XLOC_034924 | 0 | 0 | 0 | 83 | + | multiexon |
| XLOC_055144 | 0 | 0 | 4 | 0 | - | singleexon |
| XLOC_038973 | 1 | 0 | 9 | 0 | - | singleexon |
| XLOC_052760 | 0 | 0 | 0 | 7 | + | singleexon |
| XLOC_055844 | 0 | 1 | 0 | 4 | + | singleexon |
| XLOC_029773 | 0 | 0 | 4 | 0 | - | singleexon |
| XLOC_028225 | 0 | 0 | 1 | 4 | + | singleexon |
| XLOC_059112 | 0 | 1 | 4 | 0 | - | singleexon |
| XLOC_027414 | 0 | 3 | 1 | 6 | + | singleexon |
| XLOC_024506 | 0 | 0 | 3 | 0 | - | singleexon |
| XLOC_031242 | 0 | 0 | 4 | 0 | - | singleexon |
| XLOC_036856 | 0 | 0 | 11 | 0 | - | singleexon |
| XLOC_015031 | 0 | 0 | 0 | 14 | + | singleexon |
| XLOC_048770 | 1 | 0 | 2 | 2 | - | singleexon |
| XLOC_056051 | 0 | 0 | 4 | 0 | - | singleexon |
| XLOC_033642 | 0 | 0 | 0 | 25 | + | multiexon |
| XLOC_036757 | 0 | 0 | 1 | 3 | + | singleexon |
| XLOC_033723 | 0 | 0 | 0 | 6 | + | multiexon |
| XLOC_020595 | 0 | 4 | 0 | 1 | + | singleexon |
| XLOC_064016 | 10 | 0 | 6 | 2 | - | singleexon |
| XLOC_004467 | 2 | 1 | 2 | 5 | + | singleexon |
| XLOC_040526 | 0 | 0 | 0 | 5 | + | multiexon |
| XLOC_028274 | 0 | 1 | 6 | 0 | - | singleexon |
| XLOC_027278 | 0 | 1 | 3 | 0 | - | singleexon |
| XLOC_056080 | 0 | 0 | 4 | 0 | - | singleexon |
| XLOC_010199 | 0 | 0 | 0 | 3 | + | singleexon |
| XLOC_022990 | 0 | 0 | 7 | 0 | - | singleexon |
| XLOC_001265 | 0 | 1 | 0 | 2 | + | multiexon |
| XLOC_005978 | 0 | 0 | 2 | 3 | + | singleexon |
| XLOC_013717 | 0 | 0 | 1 | 10 | + | singleexon |
| XLOC_006805 | 0 | 0 | 0 | 7 | + | singleexon |
| XLOC_060741 | 0 | 0 | 0 | 21 | + | singleexon |
| XLOC_011481 | 0 | 0 | 0 | 5 | + | singleexon |
| XLOC_002728 | 0 | 1 | 0 | 3 | + | singleexon |
| XLOC_028331 | 0 | 0 | 0 | 8 | + | singleexon |
| XLOC_054892 | 0 | 0 | 0 | 3 | + | multiexon |
| XLOC_023398 | 5 | 2 | 7 | 9 | + | singleexon |
| XLOC_063051 | 0 | 19 | 0 | 0 | + | singleexon |
| XLOC_032779 | 0 | 0 | 0 | 4 | + | singleexon |
| XLOC_003981 | 0 | 1 | 1 | 2 | + | singleexon |
| XLOC_036081 | 0 | 0 | 3 | 0 | - | singleexon |
| XLOC_032906 | 2 | 0 | 2 | 0 | - | singleexon |
| XLOC_061906 | 1 | 0 | 5 | 0 | - | singleexon |
| XLOC_016124 | 2 | 0 | 0 | 4 | + | multiexon |
| XLOC_011575 | 0 | 0 | 5 | 0 | - | singleexon |
| XLOC_010693 | 0 | 0 | 17 | 1 | - | singleexon |
| XLOC_013895 | 0 | 0 | 0 | 3 | + | multiexon |
| XLOC_026911 | 0 | 0 | 3 | 0 | - | multiexon |
| XLOC_044943 | 1 | 0 | 5 | 0 | - | singleexon |
| XLOC_029283 | 0 | 0 | 3 | 0 | - | multiexon |
| XLOC_014068 | 0 | 0 | 0 | 6 | + | singleexon |
| XLOC_034941 | 0 | 0 | 5 | 0 | - | singleexon |
| XLOC_001839 | 6 | 72 | 0 | 2 | + | singleexon |
| XLOC_035225 | 1 | 0 | 3 | 1 | - | singleexon |
| XLOC_016502 | 0 | 0 | 6 | 0 | - | singleexon |
| XLOC_036812 | 0 | 0 | 0 | 7 | + | singleexon |
| XLOC_008603 | 0 | 0 | 26 | 0 | - | singleexon |
| XLOC_044057 | 0 | 0 | 4 | 0 | - | singleexon |
| XLOC_062295 | 0 | 0 | 0 | 4 | + | singleexon |
| XLOC_048591 | 0 | 0 | 3 | 0 | - | singleexon |
| XLOC_052622 | 0 | 1 | 10 | 0 | - | singleexon |
| XLOC_011410 | 0 | 0 | 0 | 11 | + | singleexon |
| XLOC_048598 | 0 | 0 | 6 | 0 | - | singleexon |
| XLOC_061614 | 0 | 1 | 4 | 2 | - | singleexon |
| XLOC_064232 | 2 | 1 | 1 | 1 | - | multiexon |
| XLOC_042263 | 18 | 0 | 17 | 1 | - | multiexon |
| XLOC_062453 | 0 | 0 | 25 | 1 | - | singleexon |
| XLOC_018141 | 0 | 0 | 5 | 0 | - | singleexon |
| XLOC_041753 | 3 | 0 | 0 | 0 | - | singleexon |
| XLOC_062675 | 0 | 0 | 1 | 8 | + | multiexon |
| XLOC_024696 | 0 | 0 | 2 | 3 | + | singleexon |
| XLOC_007611 | 0 | 0 | 0 | 4 | + | singleexon |
| XLOC_030992 | 0 | 0 | 0 | 4 | + | singleexon |
| XLOC_029596 | 0 | 45 | 20 | 0 | + | singleexon |
| XLOC_059918 | 0 | 52 | 0 | 0 | + | singleexon |
| XLOC_054429 | 0 | 1 | 0 | 7 | + | singleexon |
| XLOC_045392 | 0 | 0 | 6 | 0 | - | singleexon |
| XLOC_064545 | 1 | 0 | 3 | 1 | - | singleexon |
| XLOC_021641 | 6 | 0 | 0 | 1 | - | singleexon |
| XLOC_054880 | 0 | 3 | 1 | 28 | + | singleexon |
| XLOC_037984 | 0 | 0 | 3 | 1 | - | singleexon |
| XLOC_024738 | 0 | 0 | 0 | 12 | + | singleexon |
| XLOC_019430 | 0 | 0 | 3 | 0 | - | singleexon |
| XLOC_063415 | 4 | 0 | 4 | 0 | - | singleexon |
| XLOC_062800 | 0 | 0 | 0 | 7 | + | singleexon |
| XLOC_023835 | 2 | 0 | 49 | 4 | - | singleexon |
| XLOC_016297 | 1 | 0 | 2 | 0 | - | singleexon |
| XLOC_049948 | 1 | 0 | 4 | 1 | - | singleexon |
| XLOC_014847 | 0 | 0 | 4 | 0 | - | singleexon |
| XLOC_052875 | 1 | 0 | 3 | 0 | - | singleexon |
| XLOC_017512 | 0 | 0 | 3 | 0 | - | singleexon |
| XLOC_006822 | 0 | 0 | 2 | 8 | + | singleexon |
| XLOC_059323 | 0 | 0 | 17 | 0 | - | singleexon |
| XLOC_038017 | 3 | 1 | 1 | 9 | + | multiexon |
| XLOC_061634 | 3 | 0 | 3 | 7 | + | singleexon |
| XLOC_029846 | 2 | 0 | 1 | 2 | - | singleexon |
| XLOC_003086 | 0 | 0 | 5 | 0 | - | singleexon |
| XLOC_061205 | 0 | 0 | 0 | 6 | + | singleexon |
| XLOC_042491 | 0 | 0 | 5 | 0 | - | multiexon |
| XLOC_051542 | 0 | 0 | 10 | 1 | - | singleexon |
| XLOC_063213 | 0 | 0 | 5 | 0 | - | singleexon |
| XLOC_017287 | 0 | 1 | 5 | 0 | - | singleexon |
| XLOC_033105 | 0 | 0 | 0 | 8 | + | singleexon |
| XLOC_022181 | 0 | 0 | 0 | 7 | + | singleexon |
| XLOC_049550 | 0 | 0 | 3 | 0 | - | singleexon |
| XLOC_040437 | 0 | 0 | 1 | 11 | + | singleexon |
| XLOC_020953 | 0 | 0 | 3 | 0 | - | singleexon |
| XLOC_049361 | 0 | 0 | 0 | 5 | + | singleexon |
| XLOC_049410 | 2 | 1 | 5 | 5 | - | singleexon |
| XLOC_024030 | 0 | 3 | 0 | 0 | + | multiexon |
| XLOC_018622 | 0 | 0 | 1 | 5 | + | multiexon |
| XLOC_014600 | 0 | 1 | 3 | 0 | - | singleexon |
| XLOC_018904 | 1 | 0 | 2 | 1 | - | singleexon |
| XLOC_013461 | 0 | 0 | 5 | 0 | - | singleexon |
| XLOC_060698 | 0 | 0 | 0 | 5 | + | singleexon |
| XLOC_010280 | 0 | 0 | 6 | 3 | - | singleexon |
| XLOC_026579 | 0 | 3 | 3 | 10 | + | multiexon |
| XLOC_030469 | 0 | 1 | 10 | 0 | - | singleexon |
| XLOC_018954 | 0 | 0 | 1 | 4 | + | singleexon |
| XLOC_029828 | 0 | 0 | 0 | 8 | + | singleexon |
| XLOC_040462 | 18 | 0 | 18 | 1 | - | multiexon |
| XLOC_033467 | 0 | 1 | 1 | 2 | + | singleexon |
| XLOC_022932 | 0 | 0 | 5 | 0 | - | singleexon |
| XLOC_049923 | 0 | 0 | 2 | 4 | + | singleexon |
| XLOC_056917 | 1 | 0 | 10 | 7 | - | multiexon |
| XLOC_014861 | 0 | 0 | 9 | 0 | - | singleexon |
| XLOC_031135 | 0 | 1 | 0 | 2 | + | singleexon |
| XLOC_046014 | 0 | 0 | 3 | 0 | - | singleexon |
| XLOC_014817 | 0 | 0 | 8 | 0 | - | singleexon |
| XLOC_056858 | 0 | 0 | 2 | 3 | + | singleexon |
| XLOC_047089 | 0 | 0 | 2 | 4 | + | singleexon |
| XLOC_032897 | 0 | 0 | 1 | 9 | + | singleexon |
| XLOC_047049 | 0 | 0 | 4 | 0 | - | singleexon |
| XLOC_037501 | 0 | 0 | 3 | 0 | - | singleexon |
| XLOC_036251 | 1 | 0 | 20 | 0 | - | singleexon |
| XLOC_025795 | 0 | 0 | 7 | 0 | - | singleexon |
| XLOC_036751 | 0 | 0 | 0 | 10 | + | singleexon |
| XLOC_008275 | 1 | 1 | 2 | 1 | - | multiexon |
| XLOC_013278 | 0 | 0 | 3 | 0 | - | singleexon |
| XLOC_029092 | 4 | 0 | 0 | 0 | - | multiexon |
| XLOC_040555 | 0 | 0 | 3 | 0 | - | singleexon |
| XLOC_050950 | 0 | 0 | 0 | 27 | + | singleexon |
| XLOC_038908 | 0 | 0 | 4 | 0 | - | singleexon |
| XLOC_016746 | 0 | 1 | 2 | 513 | + | singleexon |
| XLOC_037359 | 0 | 0 | 11 | 0 | - | singleexon |
| XLOC_011385 | 0 | 0 | 0 | 3 | + | singleexon |
| XLOC_017291 | 0 | 0 | 3 | 0 | - | singleexon |
| XLOC_020479 | 4 | 1 | 1 | 0 | - | singleexon |
| XLOC_059550 | 0 | 0 | 4 | 0 | - | singleexon |
| XLOC_028336 | 0 | 0 | 2 | 4 | + | singleexon |
| XLOC_001612 | 0 | 0 | 0 | 5 | + | multiexon |
| XLOC_043374 | 0 | 0 | 0 | 11 | + | singleexon |
| XLOC_022697 | 0 | 0 | 0 | 6 | + | singleexon |
| XLOC_025556 | 0 | 0 | 5 | 0 | - | singleexon |
| XLOC_037125 | 0 | 0 | 3 | 0 | - | singleexon |
| XLOC_059857 | 1 | 0 | 22 | 0 | - | multiexon |
| XLOC_061862 | 0 | 0 | 0 | 4 | + | singleexon |
| XLOC_062980 | 0 | 1 | 0 | 4 | + | singleexon |
| XLOC_002287 | 0 | 0 | 6 | 123 | + | singleexon |
| XLOC_026829 | 0 | 0 | 1 | 19 | + | singleexon |
| XLOC_034797 | 0 | 0 | 4 | 2 | - | singleexon |
| XLOC_000228 | 1 | 0 | 10 | 0 | - | singleexon |
| XLOC_017646 | 0 | 0 | 3 | 2 | - | singleexon |
| XLOC_021041 | 0 | 0 | 7 | 0 | - | singleexon |
| XLOC_006584 | 0 | 0 | 0 | 3 | + | singleexon |
| XLOC_044926 | 0 | 0 | 4 | 0 | - | singleexon |
| XLOC_051660 | 5 | 0 | 19 | 0 | - | singleexon |
| XLOC_052909 | 0 | 0 | 4 | 2 | - | singleexon |
| XLOC_062029 | 63 | 9 | 57 | 25 | - | singleexon |
| XLOC_006806 | 0 | 0 | 0 | 5 | + | singleexon |
| XLOC_060836 | 0 | 0 | 3 | 0 | - | singleexon |
| XLOC_016060 | 0 | 0 | 0 | 5 | + | singleexon |
| XLOC_003836 | 1 | 0 | 15 | 0 | - | multiexon |
| XLOC_053296 | 0 | 1 | 3 | 15 | + | singleexon |
| XLOC_005442 | 0 | 0 | 0 | 4 | + | singleexon |
| XLOC_013884 | 1 | 0 | 3 | 0 | - | singleexon |
| XLOC_029043 | 1 | 0 | 7 | 0 | - | multiexon |
| XLOC_011220 | 0 | 0 | 7 | 0 | - | singleexon |
| XLOC_014764 | 2 | 0 | 6 | 4 | - | multiexon |
| XLOC_022178 | 0 | 0 | 4 | 0 | - | singleexon |
| XLOC_016752 | 0 | 1 | 1 | 2 | + | singleexon |
| XLOC_027950 | 0 | 0 | 0 | 16 | + | singleexon |
| XLOC_017060 | 0 | 0 | 5 | 0 | - | singleexon |
| XLOC_017131 | 0 | 3 | 0 | 0 | + | singleexon |
| XLOC_060808 | 0 | 0 | 0 | 4 | + | singleexon |
| XLOC_032774 | 0 | 0 | 0 | 12 | + | singleexon |
| XLOC_014593 | 42 | 2 | 6 | 17 | - | singleexon |
| XLOC_029629 | 0 | 0 | 2 | 36 | + | singleexon |
| XLOC_038484 | 0 | 1 | 3 | 40 | + | singleexon |
| XLOC_042755 | 0 | 1 | 4 | 0 | - | multiexon |
| XLOC_028845 | 0 | 20 | 0 | 1 | + | singleexon |
| XLOC_044700 | 1 | 1 | 2 | 1 | - | singleexon |
| XLOC_053308 | 0 | 0 | 13 | 0 | - | singleexon |
| XLOC_037165 | 0 | 1 | 3 | 0 | - | singleexon |
| XLOC_027746 | 0 | 0 | 1 | 3 | + | singleexon |
| XLOC_019454 | 0 | 0 | 0 | 7 | + | singleexon |
| XLOC_005351 | 0 | 0 | 4 | 0 | - | singleexon |
| XLOC_061918 | 1 | 0 | 6 | 0 | - | singleexon |
| XLOC_050498 | 0 | 0 | 0 | 4 | + | singleexon |
| XLOC_028218 | 0 | 0 | 4 | 0 | - | singleexon |
| XLOC_053059 | 0 | 0 | 0 | 11 | + | singleexon |
| XLOC_022161 | 0 | 0 | 4 | 0 | - | singleexon |
| XLOC_028158 | 0 | 0 | 0 | 6 | + | singleexon |
| XLOC_051967 | 0 | 0 | 9 | 3 | - | singleexon |
| XLOC_044711 | 3 | 0 | 0 | 5 | + | singleexon |
| XLOC_022912 | 1 | 0 | 2 | 0 | - | singleexon |
| XLOC_062435 | 0 | 0 | 0 | 3 | + | singleexon |
| XLOC_013531 | 0 | 0 | 21 | 0 | - | singleexon |
| XLOC_027104 | 0 | 1 | 9 | 0 | - | singleexon |
| XLOC_041631 | 0 | 0 | 1 | 11 | + | singleexon |
| XLOC_063210 | 1 | 1 | 2 | 0 | - | singleexon |
| XLOC_054672 | 0 | 0 | 8 | 0 | - | singleexon |
| XLOC_026479 | 0 | 0 | 1 | 7 | + | multiexon |
| XLOC_014368 | 4 | 0 | 0 | 0 | - | singleexon |
| XLOC_044046 | 0 | 0 | 38 | 0 | - | singleexon |
| XLOC_006626 | 0 | 17 | 4 | 8 | + | singleexon |
| XLOC_017219 | 0 | 0 | 3 | 0 | - | singleexon |
| XLOC_019510 | 0 | 0 | 0 | 5 | + | singleexon |
| XLOC_060007 | 7 | 0 | 0 | 0 | - | singleexon |
| XLOC_062875 | 0 | 1 | 3 | 0 | - | singleexon |
| XLOC_033215 | 1 | 2 | 0 | 1 | + | singleexon |
| XLOC_057128 | 0 | 1 | 0 | 3 | + | singleexon |
| XLOC_036223 | 0 | 0 | 0 | 4 | + | singleexon |
| XLOC_002672 | 4 | 0 | 6 | 0 | - | singleexon |
| XLOC_022196 | 0 | 2 | 0 | 1 | + | singleexon |
| XLOC_038203 | 0 | 0 | 3 | 0 | - | singleexon |
| XLOC_007720 | 0 | 0 | 18 | 0 | - | singleexon |
| XLOC_015620 | 0 | 0 | 18 | 0 | - | singleexon |
| XLOC_016315 | 0 | 0 | 0 | 3 | + | singleexon |
| XLOC_035315 | 0 | 0 | 0 | 7 | + | singleexon |
| XLOC_034848 | 0 | 0 | 0 | 3 | + | singleexon |
| XLOC_046488 | 0 | 0 | 0 | 3 | + | singleexon |
| XLOC_036717 | 0 | 0 | 0 | 5 | + | singleexon |
| XLOC_012680 | 0 | 1 | 3 | 0 | - | singleexon |
| XLOC_058434 | 2 | 0 | 6 | 1 | - | singleexon |
| XLOC_029519 | 0 | 0 | 0 | 11 | + | singleexon |
| XLOC_039631 | 7 | 0 | 13 | 0 | - | singleexon |
| XLOC_052625 | 0 | 0 | 6 | 0 | - | singleexon |
| XLOC_022679 | 0 | 0 | 0 | 3 | + | singleexon |
| XLOC_052545 | 0 | 0 | 0 | 4 | + | singleexon |
| XLOC_010234 | 0 | 0 | 0 | 24 | + | singleexon |
| XLOC_035367 | 1 | 0 | 11 | 1 | - | singleexon |
| XLOC_009404 | 0 | 0 | 1 | 13 | + | multiexon |
| XLOC_013585 | 1 | 12 | 1 | 28 | + | singleexon |
| XLOC_022977 | 0 | 2 | 1 | 8 | + | singleexon |
| XLOC_030630 | 0 | 0 | 4 | 0 | - | singleexon |
| XLOC_041093 | 0 | 0 | 0 | 5 | + | multiexon |
| XLOC_048232 | 0 | 0 | 1 | 16 | + | singleexon |
| XLOC_059351 | 0 | 0 | 5 | 1 | - | singleexon |
| XLOC_007367 | 1 | 0 | 1 | 21 | + | singleexon |
| XLOC_014080 | 1 | 0 | 1 | 4 | + | singleexon |
| XLOC_033748 | 0 | 0 | 0 | 40 | + | multiexon |
| XLOC_002582 | 0 | 0 | 3 | 0 | - | singleexon |
| XLOC_058477 | 0 | 0 | 0 | 3 | + | singleexon |
| XLOC_011711 | 0 | 0 | 9 | 2 | - | multiexon |
| XLOC_033534 | 0 | 0 | 0 | 37 | + | singleexon |
| XLOC_014964 | 0 | 0 | 9 | 25 | + | singleexon |
| XLOC_034061 | 0 | 1 | 0 | 3 | + | multiexon |
| XLOC_064469 | 1 | 1 | 3 | 8 | + | multiexon |
| XLOC_032782 | 2 | 2 | 2 | 12 | + | singleexon |
| XLOC_018964 | 0 | 0 | 1 | 3 | + | singleexon |
| XLOC_039078 | 0 | 0 | 20 | 3 | - | singleexon |
| XLOC_010827 | 0 | 0 | 0 | 14 | + | singleexon |
| XLOC_061768 | 2 | 4 | 1 | 4 | + | singleexon |
| XLOC_020436 | 0 | 0 | 4 | 0 | - | singleexon |
| XLOC_013783 | 0 | 4 | 3 | 3 | + | singleexon |
| XLOC_022711 | 0 | 0 | 0 | 14 | + | singleexon |
| XLOC_059931 | 3 | 0 | 6 | 0 | - | singleexon |
| XLOC_014458 | 0 | 3 | 0 | 1 | + | singleexon |
| XLOC_034919 | 0 | 2 | 0 | 5 | + | singleexon |
| XLOC_003843 | 0 | 0 | 0 | 17 | + | multiexon |
| XLOC_025964 | 0 | 0 | 0 | 8 | + | multiexon |
| XLOC_041924 | 0 | 0 | 6 | 0 | - | singleexon |
| XLOC_061445 | 1 | 0 | 4 | 2 | - | singleexon |
| XLOC_002548 | 0 | 21 | 4 | 2 | + | singleexon |
| XLOC_054789 | 0 | 1 | 0 | 4 | + | singleexon |
| XLOC_014021 | 0 | 0 | 1 | 16 | + | multiexon |
| XLOC_018570 | 0 | 0 | 1 | 16 | + | singleexon |
| XLOC_053973 | 0 | 0 | 3 | 3 | - | singleexon |
| XLOC_046362 | 0 | 0 | 0 | 5 | + | singleexon |
| XLOC_011246 | 0 | 0 | 4 | 0 | - | singleexon |
| XLOC_016759 | 3 | 0 | 0 | 1 | - | singleexon |
| XLOC_039272 | 0 | 4 | 0 | 0 | + | singleexon |
| XLOC_005417 | 0 | 0 | 0 | 13 | + | singleexon |
| XLOC_041754 | 0 | 0 | 5 | 0 | - | singleexon |
| XLOC_006167 | 1 | 0 | 0 | 4 | + | singleexon |
| XLOC_041836 | 4 | 0 | 0 | 2 | - | multiexon |
| XLOC_001838 | 0 | 0 | 1 | 3 | + | singleexon |
| XLOC_016859 | 0 | 0 | 3 | 0 | - | singleexon |
| XLOC_015334 | 0 | 0 | 0 | 4 | + | singleexon |
| XLOC_027085 | 0 | 2 | 0 | 2 | + | singleexon |
| XLOC_029513 | 3 | 0 | 0 | 0 | - | singleexon |
| XLOC_043426 | 0 | 0 | 3 | 3 | - | singleexon |
| XLOC_013619 | 0 | 0 | 3 | 1 | - | singleexon |
| XLOC_028851 | 0 | 2 | 1 | 3 | + | singleexon |
| XLOC_052212 | 0 | 0 | 52 | 0 | - | singleexon |
| XLOC_004177 | 2 | 0 | 1 | 0 | - | multiexon |
| XLOC_022772 | 0 | 0 | 0 | 4 | + | singleexon |
| XLOC_050188 | 0 | 0 | 6 | 1 | - | singleexon |
| XLOC_035695 | 192 | 0 | 0 | 24 | - | singleexon |
| XLOC_017004 | 1 | 0 | 0 | 17 | + | multiexon |
| XLOC_019104 | 0 | 1 | 0 | 3 | + | singleexon |
| XLOC_028885 | 25 | 2 | 29 | 0 | - | singleexon |
| XLOC_059904 | 1 | 0 | 1 | 4 | + | singleexon |
| XLOC_015052 | 0 | 0 | 5 | 0 | - | singleexon |
| XLOC_007773 | 0 | 0 | 2 | 21 | + | multiexon |
| XLOC_019892 | 1 | 0 | 0 | 98 | + | multiexon |
| XLOC_018768 | 0 | 0 | 6 | 0 | - | singleexon |
| XLOC_004280 | 0 | 0 | 3 | 0 | - | singleexon |
| XLOC_027419 | 0 | 0 | 8 | 0 | - | singleexon |
| XLOC_053770 | 0 | 0 | 0 | 10 | + | multiexon |
| XLOC_040671 | 0 | 0 | 3 | 0 | - | singleexon |
| XLOC_039298 | 0 | 0 | 0 | 6 | + | singleexon |
| XLOC_043745 | 2 | 0 | 1 | 0 | - | singleexon |
| XLOC_023379 | 0 | 1 | 0 | 9 | + | singleexon |
| XLOC_023612 | 0 | 0 | 3 | 12 | + | singleexon |
| XLOC_011905 | 0 | 0 | 4 | 3 | - | singleexon |
| XLOC_022818 | 0 | 0 | 0 | 4 | + | singleexon |
| XLOC_059273 | 0 | 1 | 9 | 0 | - | singleexon |
| XLOC_061861 | 0 | 0 | 0 | 3 | + | singleexon |
| XLOC_058988 | 0 | 1 | 0 | 8 | + | multiexon |
| XLOC_039826 | 3 | 4 | 0 | 2 | + | singleexon |
| XLOC_035057 | 1 | 1 | 2 | 0 | - | singleexon |
| XLOC_032898 | 0 | 0 | 0 | 3 | + | singleexon |
| XLOC_018829 | 0 | 0 | 0 | 3 | + | singleexon |
| XLOC_018464 | 0 | 0 | 0 | 5 | + | singleexon |
| XLOC_032956 | 0 | 1 | 24 | 1 | - | singleexon |
| XLOC_047347 | 0 | 0 | 7 | 0 | - | singleexon |
| XLOC_017310 | 0 | 0 | 11 | 0 | - | singleexon |
| XLOC_007125 | 0 | 0 | 3 | 0 | - | singleexon |
| XLOC_036208 | 0 | 0 | 4 | 0 | - | multiexon |
| XLOC_012644 | 0 | 0 | 8 | 0 | - | singleexon |
| XLOC_036015 | 3 | 0 | 3 | 8 | + | multiexon |
| XLOC_052573 | 2 | 1 | 16 | 1 | - | singleexon |
| XLOC_054449 | 1 | 0 | 0 | 3 | + | singleexon |
| XLOC_017802 | 0 | 0 | 4 | 0 | - | singleexon |
| XLOC_016670 | 0 | 0 | 4 | 11 | + | singleexon |
| XLOC_017301 | 0 | 0 | 11 | 0 | - | singleexon |
| XLOC_040112 | 0 | 0 | 0 | 58 | + | singleexon |
| XLOC_020782 | 0 | 0 | 0 | 33 | + | multiexon |
| XLOC_059406 | 0 | 0 | 18 | 6 | - | singleexon |
| XLOC_024368 | 1 | 2 | 0 | 2 | + | singleexon |
| XLOC_004897 | 0 | 0 | 1 | 7 | + | singleexon |
| XLOC_044834 | 0 | 0 | 4 | 0 | - | singleexon |
| XLOC_032415 | 1 | 0 | 7 | 0 | - | singleexon |
| XLOC_007569 | 0 | 0 | 6 | 1 | - | singleexon |
| XLOC_039042 | 0 | 0 | 4 | 0 | - | singleexon |
| XLOC_043778 | 1 | 0 | 11 | 0 | - | singleexon |
| XLOC_056068 | 0 | 0 | 6 | 0 | - | singleexon |
| XLOC_035664 | 0 | 0 | 30 | 0 | - | singleexon |
| XLOC_044292 | 0 | 3 | 1 | 0 | + | singleexon |
| XLOC_019548 | 0 | 1 | 1 | 9 | + | singleexon |
| XLOC_058209 | 0 | 0 | 0 | 9 | + | multiexon |
| XLOC_016140 | 0 | 0 | 3 | 0 | - | multiexon |
| XLOC_048622 | 0 | 0 | 3 | 1 | - | singleexon |
| XLOC_022710 | 0 | 0 | 0 | 3 | + | singleexon |
| XLOC_010124 | 0 | 1 | 12 | 0 | - | multiexon |
| XLOC_019990 | 0 | 0 | 7 | 0 | - | singleexon |
| XLOC_034854 | 0 | 0 | 3 | 1 | - | singleexon |
| XLOC_049920 | 0 | 0 | 3 | 0 | - | singleexon |
| XLOC_038212 | 0 | 0 | 4 | 0 | - | singleexon |
| XLOC_046030 | 0 | 0 | 12 | 0 | - | multiexon |
| XLOC_024386 | 0 | 1 | 7 | 0 | - | singleexon |
| XLOC_007324 | 0 | 0 | 5 | 0 | - | multiexon |
| XLOC_035359 | 0 | 0 | 1 | 11 | + | singleexon |
| XLOC_015587 | 0 | 0 | 0 | 3 | + | singleexon |
| XLOC_053732 | 0 | 0 | 0 | 4 | + | singleexon |
| XLOC_045394 | 0 | 0 | 0 | 3 | + | multiexon |
| XLOC_027979 | 0 | 0 | 0 | 9 | + | singleexon |
| XLOC_010477 | 1 | 0 | 6 | 0 | - | multiexon |
| XLOC_031110 | 0 | 0 | 0 | 3 | + | singleexon |
| XLOC_041987 | 0 | 0 | 6 | 0 | - | singleexon |
| XLOC_004871 | 0 | 0 | 1 | 3 | + | singleexon |
| XLOC_018479 | 0 | 0 | 12 | 0 | - | singleexon |
| XLOC_033086 | 0 | 0 | 0 | 4 | + | singleexon |
| XLOC_055118 | 0 | 0 | 3 | 0 | - | singleexon |
| XLOC_050883 | 0 | 0 | 11 | 0 | - | singleexon |
| XLOC_040030 | 0 | 0 | 0 | 11 | + | singleexon |
| XLOC_007710 | 0 | 0 | 3 | 0 | - | singleexon |
| XLOC_027479 | 0 | 0 | 2 | 4 | + | singleexon |
| XLOC_035713 | 0 | 0 | 7 | 0 | - | singleexon |
| XLOC_028339 | 0 | 0 | 1 | 8 | + | singleexon |
| XLOC_039632 | 0 | 0 | 3 | 0 | - | singleexon |
| XLOC_040350 | 0 | 0 | 12 | 0 | - | singleexon |
| XLOC_012559 | 0 | 0 | 3 | 2 | - | singleexon |
| XLOC_028470 | 0 | 0 | 3 | 0 | - | singleexon |
| XLOC_007416 | 0 | 0 | 0 | 5 | + | singleexon |
| XLOC_039705 | 0 | 0 | 1 | 4 | + | singleexon |
| XLOC_052071 | 0 | 0 | 1 | 18 | + | singleexon |
| XLOC_050156 | 2 | 0 | 3 | 0 | - | singleexon |
| XLOC_005395 | 1 | 2 | 1 | 6 | + | singleexon |
| XLOC_015764 | 0 | 0 | 10 | 0 | - | singleexon |
| XLOC_008581 | 0 | 0 | 5 | 0 | - | singleexon |
| XLOC_001808 | 0 | 2 | 3 | 0 | - | multiexon |
| XLOC_037094 | 0 | 0 | 17 | 0 | - | singleexon |
| XLOC_008087 | 1 | 0 | 4 | 1 | - | singleexon |
| XLOC_017627 | 0 | 0 | 0 | 5 | + | singleexon |
| XLOC_026208 | 0 | 0 | 1 | 4 | + | multiexon |
| XLOC_018837 | 0 | 0 | 0 | 19 | + | singleexon |
| XLOC_061494 | 0 | 1 | 0 | 3 | + | singleexon |
| XLOC_017362 | 0 | 1 | 0 | 4 | + | singleexon |
| XLOC_040562 | 0 | 0 | 8 | 0 | - | singleexon |
| XLOC_058688 | 0 | 0 | 6 | 0 | - | singleexon |
| XLOC_048682 | 9 | 2 | 0 | 1 | - | singleexon |
| XLOC_025643 | 0 | 0 | 0 | 3 | + | singleexon |
| XLOC_051906 | 1 | 14 | 0 | 6 | + | singleexon |
| XLOC_013458 | 0 | 0 | 9 | 0 | - | singleexon |
| XLOC_058235 | 2 | 0 | 2 | 0 | - | singleexon |
| XLOC_015859 | 0 | 1 | 12 | 0 | - | singleexon |
| XLOC_011730 | 0 | 1 | 35 | 0 | - | multiexon |
| XLOC_027811 | 0 | 1 | 16 | 43 | + | singleexon |
| XLOC_020140 | 0 | 0 | 0 | 12 | + | singleexon |
| XLOC_035948 | 0 | 0 | 0 | 6 | + | singleexon |
| XLOC_022668 | 1 | 0 | 0 | 7 | + | multiexon |
| XLOC_013983 | 0 | 0 | 3 | 0 | - | singleexon |
| XLOC_042167 | 0 | 0 | 1 | 4 | + | singleexon |
| XLOC_019670 | 0 | 0 | 4 | 0 | - | multiexon |
| XLOC_038049 | 0 | 0 | 0 | 14 | + | singleexon |
| XLOC_038344 | 0 | 0 | 10 | 0 | - | singleexon |
| XLOC_057709 | 0 | 0 | 6 | 2 | - | multiexon |
| XLOC_019962 | 0 | 1 | 73 | 0 | - | singleexon |
| XLOC_008622 | 7 | 0 | 2 | 3 | - | singleexon |
| XLOC_049357 | 0 | 0 | 0 | 6 | + | singleexon |
| XLOC_007992 | 1 | 0 | 0 | 4 | + | singleexon |
| XLOC_047271 | 0 | 0 | 3 | 0 | - | multiexon |
| XLOC_021845 | 1 | 0 | 60 | 4 | - | singleexon |
| XLOC_003718 | 0 | 0 | 0 | 6 | + | singleexon |
| XLOC_040426 | 0 | 0 | 1 | 3 | + | singleexon |
| XLOC_022685 | 0 | 0 | 0 | 6 | + | singleexon |
| XLOC_035247 | 0 | 0 | 0 | 7 | + | singleexon |
| XLOC_037339 | 0 | 0 | 5 | 0 | - | singleexon |
| XLOC_020285 | 0 | 1 | 0 | 3 | + | singleexon |
| XLOC_002109 | 0 | 0 | 0 | 3 | + | multiexon |
| XLOC_017923 | 0 | 0 | 5 | 0 | - | singleexon |
| XLOC_003912 | 0 | 0 | 0 | 10 | + | singleexon |
| XLOC_031442 | 0 | 0 | 1 | 4 | + | singleexon |
| XLOC_016685 | 1 | 1 | 2 | 0 | - | singleexon |
| XLOC_013292 | 0 | 0 | 3 | 0 | - | singleexon |
| XLOC_037642 | 0 | 0 | 5 | 0 | - | multiexon |
| XLOC_000246 | 1 | 0 | 14 | 1 | - | singleexon |
| XLOC_052849 | 0 | 0 | 0 | 8 | + | singleexon |
| XLOC_026876 | 0 | 0 | 0 | 3 | + | singleexon |
| XLOC_030640 | 0 | 0 | 4 | 0 | - | singleexon |
| XLOC_057967 | 0 | 0 | 0 | 9 | + | singleexon |
| XLOC_045863 | 0 | 0 | 22 | 2 | - | singleexon |
| XLOC_010686 | 0 | 0 | 0 | 6 | + | singleexon |
| XLOC_005121 | 0 | 0 | 1 | 3 | + | singleexon |
| XLOC_062404 | 0 | 0 | 1 | 3 | + | singleexon |
| XLOC_023769 | 0 | 0 | 1 | 4 | + | multiexon |
| XLOC_006366 | 0 | 6 | 6 | 0 | + | singleexon |
| XLOC_045171 | 7 | 3 | 0 | 56 | + | multiexon |
| XLOC_005278 | 1 | 0 | 4 | 0 | - | singleexon |
| XLOC_059689 | 0 | 0 | 0 | 3 | + | singleexon |
| XLOC_062051 | 0 | 0 | 1 | 5 | + | singleexon |
| XLOC_003626 | 0 | 0 | 3 | 0 | - | singleexon |
| XLOC_049806 | 0 | 0 | 3 | 0 | - | singleexon |
| XLOC_046650 | 0 | 0 | 1 | 3 | + | singleexon |
| XLOC_021864 | 0 | 2 | 3 | 3 | - | multiexon |
| XLOC_056314 | 0 | 0 | 3 | 1 | - | singleexon |
| XLOC_020133 | 1 | 0 | 4 | 0 | - | singleexon |
| XLOC_017800 | 0 | 0 | 0 | 4 | + | singleexon |
| XLOC_052085 | 0 | 0 | 0 | 4 | + | singleexon |
| XLOC_020451 | 0 | 0 | 4 | 0 | - | singleexon |
| XLOC_034870 | 0 | 0 | 0 | 3 | + | singleexon |
| XLOC_020596 | 1 | 0 | 7 | 0 | - | singleexon |
| XLOC_020163 | 0 | 2 | 0 | 1 | + | multiexon |
| XLOC_011756 | 1 | 0 | 5 | 0 | - | singleexon |
| XLOC_027348 | 0 | 0 | 1 | 8 | + | singleexon |
| XLOC_053246 | 0 | 0 | 8 | 0 | - | singleexon |
| XLOC_012518 | 0 | 0 | 13 | 0 | - | singleexon |
| XLOC_012622 | 1 | 0 | 0 | 3 | + | singleexon |
| XLOC_019131 | 0 | 0 | 7 | 0 | - | singleexon |
| XLOC_033508 | 3 | 0 | 5 | 0 | - | singleexon |
| XLOC_017825 | 0 | 1 | 0 | 2 | + | singleexon |
| XLOC_032784 | 0 | 2 | 1 | 3 | + | singleexon |
| XLOC_012826 | 0 | 0 | 0 | 14 | + | singleexon |
| XLOC_058277 | 0 | 0 | 0 | 23 | + | singleexon |
| XLOC_062069 | 0 | 0 | 6 | 17 | + | multiexon |
| XLOC_003655 | 0 | 0 | 9 | 0 | - | singleexon |
| XLOC_028879 | 0 | 0 | 0 | 6 | + | singleexon |
| XLOC_017023 | 0 | 0 | 1 | 6 | + | singleexon |
| XLOC_014434 | 0 | 0 | 31 | 1 | - | singleexon |
| XLOC_063000 | 0 | 0 | 0 | 3 | + | multiexon |
| XLOC_016317 | 2 | 0 | 1 | 0 | - | singleexon |
| XLOC_056070 | 0 | 0 | 4 | 0 | - | singleexon |
| XLOC_025558 | 0 | 0 | 7 | 0 | - | singleexon |
| XLOC_017074 | 1 | 0 | 0 | 9 | + | singleexon |
| XLOC_048289 | 1 | 4 | 1 | 45 | + | singleexon |
| XLOC_018463 | 0 | 0 | 0 | 4 | + | singleexon |
| XLOC_014815 | 0 | 0 | 4 | 6 | + | singleexon |
| XLOC_013618 | 0 | 0 | 0 | 4 | + | singleexon |
| XLOC_063399 | 0 | 0 | 0 | 7 | + | singleexon |
| XLOC_025493 | 0 | 0 | 11 | 0 | - | multiexon |
| XLOC_007994 | 1 | 0 | 7 | 1 | - | singleexon |
| XLOC_006831 | 0 | 0 | 0 | 3 | + | singleexon |
| XLOC_004458 | 1 | 0 | 2 | 0 | - | multiexon |
| XLOC_018014 | 0 | 0 | 19 | 1192 | + | singleexon |
| XLOC_005823 | 0 | 0 | 1 | 3 | + | singleexon |
| XLOC_056623 | 0 | 0 | 1 | 6 | + | singleexon |
| XLOC_012617 | 2 | 0 | 2 | 6 | + | singleexon |
| XLOC_003174 | 0 | 1 | 0 | 98 | + | multiexon |
| XLOC_014986 | 0 | 0 | 4 | 0 | - | singleexon |
| XLOC_024588 | 0 | 0 | 3 | 15 | + | singleexon |
| XLOC_036396 | 1 | 0 | 63 | 1 | - | singleexon |
| XLOC_010009 | 0 | 3 | 1 | 2 | + | singleexon |
| XLOC_044065 | 0 | 0 | 7 | 0 | - | singleexon |
| XLOC_051253 | 1 | 0 | 10 | 1 | - | singleexon |
| XLOC_003717 | 4 | 0 | 0 | 0 | - | singleexon |
| XLOC_046350 | 0 | 0 | 0 | 3 | + | singleexon |
| XLOC_031125 | 0 | 0 | 2 | 4 | + | singleexon |
| XLOC_055778 | 0 | 0 | 4 | 1 | - | singleexon |
| XLOC_028200 | 0 | 0 | 11 | 0 | - | singleexon |
| XLOC_032302 | 0 | 25 | 0 | 18 | + | singleexon |
| XLOC_031238 | 1 | 0 | 9 | 1 | - | singleexon |
| XLOC_050664 | 0 | 1 | 0 | 4 | + | singleexon |
| XLOC_029339 | 0 | 0 | 1 | 3 | + | multiexon |
| XLOC_039464 | 0 | 0 | 0 | 3 | + | singleexon |
| XLOC_041181 | 2 | 3 | 1 | 0 | + | multiexon |
| XLOC_002575 | 0 | 0 | 0 | 4 | + | singleexon |
| XLOC_036996 | 1 | 1 | 11 | 0 | - | singleexon |
| XLOC_058694 | 0 | 0 | 8 | 0 | - | multiexon |
| XLOC_058429 | 0 | 0 | 0 | 4 | + | multiexon |
| XLOC_052921 | 1 | 1 | 3 | 1 | - | singleexon |
| XLOC_049371 | 0 | 1 | 0 | 16 | + | singleexon |
| XLOC_040538 | 0 | 0 | 6 | 0 | - | singleexon |
| XLOC_051076 | 0 | 0 | 0 | 3 | + | singleexon |
| XLOC_006914 | 0 | 3 | 0 | 0 | + | singleexon |
| XLOC_046004 | 1 | 0 | 24 | 0 | - | singleexon |
| XLOC_061317 | 3 | 0 | 11 | 5 | - | multiexon |
| XLOC_027395 | 0 | 1 | 2 | 6 | + | singleexon |
| XLOC_010867 | 0 | 0 | 0 | 25 | + | singleexon |
| XLOC_061245 | 1 | 0 | 0 | 76 | + | multiexon |
| XLOC_057761 | 0 | 0 | 8 | 0 | - | singleexon |
| XLOC_025035 | 0 | 0 | 4 | 0 | - | singleexon |
| XLOC_061479 | 1 | 1 | 0 | 10 | + | multiexon |
| XLOC_028852 | 0 | 0 | 0 | 99 | + | singleexon |
| XLOC_036229 | 0 | 0 | 0 | 5 | + | singleexon |
| XLOC_044709 | 0 | 107 | 1 | 2 | + | singleexon |
| XLOC_034304 | 0 | 0 | 0 | 5 | + | singleexon |
| XLOC_048461 | 0 | 0 | 0 | 7 | + | singleexon |
| XLOC_064045 | 0 | 0 | 3 | 0 | - | multiexon |
| XLOC_062040 | 2 | 0 | 1 | 2 | - | singleexon |
| XLOC_015828 | 0 | 0 | 3 | 2 | - | singleexon |
| XLOC_053669 | 0 | 0 | 1 | 4 | + | singleexon |
| XLOC_026089 | 0 | 0 | 2 | 3 | + | singleexon |
| XLOC_025178 | 0 | 0 | 0 | 31 | + | multiexon |
| XLOC_058063 | 0 | 0 | 1 | 4 | + | singleexon |
| XLOC_020795 | 0 | 0 | 4 | 1 | - | singleexon |
| XLOC_037127 | 0 | 1 | 3 | 2 | - | singleexon |
| XLOC_033515 | 8 | 0 | 0 | 10 | + | singleexon |
| XLOC_042407 | 0 | 0 | 6 | 1 | - | singleexon |
| XLOC_022852 | 10 | 0 | 6 | 6 | - | singleexon |
| XLOC_036742 | 0 | 0 | 58 | 0 | - | multiexon |
| XLOC_029634 | 0 | 1 | 0 | 4 | + | singleexon |
| XLOC_057961 | 0 | 0 | 0 | 3 | + | singleexon |
| XLOC_039642 | 1 | 0 | 2 | 0 | - | multiexon |
| XLOC_028266 | 0 | 0 | 0 | 3 | + | singleexon |
| XLOC_062032 | 0 | 0 | 0 | 7 | + | singleexon |
| XLOC_060492 | 0 | 0 | 0 | 4 | + | singleexon |
| XLOC_042243 | 0 | 0 | 0 | 4 | + | singleexon |
| XLOC_006711 | 0 | 0 | 5 | 0 | - | multiexon |
| XLOC_025153 | 0 | 0 | 0 | 3 | + | multiexon |
| XLOC_014794 | 0 | 2 | 1 | 7 | + | singleexon |
| XLOC_051217 | 0 | 0 | 8 | 0 | - | singleexon |
| XLOC_016837 | 4 | 0 | 0 | 0 | - | singleexon |
| XLOC_059926 | 4 | 0 | 7 | 0 | - | multiexon |
| XLOC_048879 | 0 | 0 | 0 | 10 | + | singleexon |
| XLOC_060270 | 1 | 4 | 3 | 19 | + | singleexon |
| XLOC_024394 | 0 | 0 | 3 | 0 | - | singleexon |
| XLOC_031553 | 19 | 1 | 0 | 0 | - | multiexon |
| XLOC_049107 | 0 | 0 | 0 | 7 | + | singleexon |
| XLOC_026409 | 0 | 1 | 2 | 3 | + | multiexon |
| XLOC_015543 | 0 | 0 | 0 | 4 | + | singleexon |
| XLOC_056732 | 1 | 0 | 2 | 0 | - | singleexon |
| XLOC_000487 | 0 | 0 | 9 | 0 | - | singleexon |
| XLOC_057398 | 0 | 0 | 28 | 0 | - | multiexon |
| XLOC_014057 | 0 | 0 | 0 | 3 | + | singleexon |
| XLOC_004354 | 5 | 1 | 6 | 6 | - | singleexon |
| XLOC_048900 | 0 | 6 | 0 | 0 | + | singleexon |
| XLOC_015250 | 0 | 0 | 0 | 10 | + | singleexon |
| XLOC_035694 | 0 | 0 | 0 | 4 | + | singleexon |
| XLOC_034674 | 0 | 1 | 3 | 0 | - | singleexon |
| XLOC_033956 | 0 | 0 | 3 | 1 | - | singleexon |
| XLOC_017266 | 0 | 0 | 10 | 0 | - | singleexon |
| XLOC_061962 | 0 | 0 | 12 | 3 | - | singleexon |
| XLOC_018656 | 1 | 0 | 0 | 4 | + | singleexon |
| XLOC_013850 | 0 | 0 | 18 | 0 | - | singleexon |
| XLOC_043017 | 0 | 0 | 0 | 9 | + | singleexon |
| XLOC_033758 | 0 | 0 | 3 | 0 | - | singleexon |
| XLOC_027905 | 0 | 0 | 4 | 0 | - | multiexon |
| XLOC_004613 | 0 | 0 | 4 | 0 | - | multiexon |
| XLOC_012426 | 0 | 1 | 0 | 2 | + | multiexon |
| XLOC_002574 | 0 | 0 | 0 | 3 | + | singleexon |
| XLOC_010944 | 0 | 0 | 3 | 0 | - | singleexon |
| XLOC_035423 | 0 | 1 | 0 | 2 | + | multiexon |
| XLOC_058913 | 2 | 1 | 9 | 2 | - | multiexon |
| XLOC_018166 | 0 | 0 | 3 | 0 | - | singleexon |
| XLOC_033225 | 0 | 0 | 0 | 8 | + | singleexon |
| XLOC_018557 | 0 | 2 | 0 | 12 | + | multiexon |
| XLOC_012586 | 0 | 0 | 0 | 4 | + | multiexon |
| XLOC_017354 | 0 | 0 | 11 | 0 | - | singleexon |
| XLOC_054829 | 0 | 0 | 3 | 0 | - | singleexon |
| XLOC_016908 | 0 | 0 | 0 | 3 | + | singleexon |
| XLOC_023039 | 0 | 0 | 3 | 0 | - | singleexon |
| XLOC_062899 | 0 | 1 | 1 | 121 | + | singleexon |
| XLOC_050221 | 13 | 0 | 0 | 0 | - | multiexon |
| XLOC_037379 | 0 | 0 | 0 | 4 | + | singleexon |
| XLOC_049090 | 0 | 0 | 0 | 4 | + | multiexon |
| XLOC_021218 | 0 | 0 | 12 | 0 | - | singleexon |
| XLOC_052427 | 0 | 0 | 20 | 0 | - | multiexon |
| XLOC_039314 | 0 | 2 | 3 | 0 | - | singleexon |
| XLOC_005024 | 2 | 0 | 11 | 0 | - | multiexon |
| XLOC_013734 | 1 | 0 | 10 | 0 | - | singleexon |
| XLOC_026261 | 0 | 2 | 0 | 5 | + | singleexon |
| XLOC_022565 | 0 | 0 | 59 | 5 | - | multiexon |
| XLOC_013718 | 0 | 0 | 1 | 8 | + | singleexon |
| XLOC_055464 | 0 | 0 | 2 | 6 | + | singleexon |
| XLOC_016278 | 0 | 0 | 4 | 1 | - | singleexon |
| XLOC_029103 | 0 | 0 | 6 | 5 | - | multiexon |
| XLOC_022737 | 0 | 0 | 0 | 87 | + | singleexon |
| XLOC_062566 | 0 | 1 | 3 | 1 | - | multiexon |
| XLOC_015742 | 0 | 0 | 5 | 1 | - | singleexon |
| XLOC_037284 | 0 | 0 | 13 | 0 | - | singleexon |
| XLOC_018427 | 0 | 0 | 0 | 18 | + | singleexon |
| XLOC_007163 | 0 | 0 | 3 | 0 | - | singleexon |
| XLOC_004934 | 0 | 0 | 9 | 0 | - | multiexon |
| XLOC_055241 | 0 | 0 | 4 | 0 | - | singleexon |
| XLOC_050659 | 1 | 0 | 0 | 5 | + | singleexon |
| XLOC_050164 | 0 | 0 | 157 | 0 | - | singleexon |
| XLOC_051910 | 38 | 0 | 4 | 124 | + | multiexon |
| XLOC_009893 | 0 | 0 | 0 | 15 | + | multiexon |
| XLOC_013050 | 1 | 0 | 2 | 1 | - | multiexon |
| XLOC_002184 | 0 | 0 | 0 | 4 | + | singleexon |
| XLOC_051647 | 0 | 0 | 1 | 25 | + | multiexon |
| XLOC_044473 | 0 | 6 | 1 | 1 | + | multiexon |
| XLOC_018124 | 0 | 0 | 5 | 1 | - | singleexon |
| XLOC_056963 | 0 | 2 | 1 | 4 | + | multiexon |
| XLOC_017321 | 0 | 0 | 4 | 0 | - | singleexon |
| XLOC_045823 | 0 | 0 | 10 | 3 | - | multiexon |
| XLOC_060418 | 0 | 0 | 0 | 21 | + | singleexon |
| XLOC_000064 | 3 | 0 | 0 | 0 | - | singleexon |
| XLOC_050346 | 1 | 3 | 10 | 2 | - | multiexon |
| XLOC_022158 | 0 | 0 | 0 | 9 | + | singleexon |
| XLOC_025925 | 1 | 0 | 7 | 0 | - | singleexon |
| XLOC_010940 | 0 | 2 | 0 | 2 | + | singleexon |
| XLOC_041948 | 1 | 3 | 0 | 1 | + | multiexon |
| XLOC_013465 | 0 | 0 | 29 | 1 | - | singleexon |
| XLOC_013617 | 1 | 0 | 2 | 2 | - | singleexon |
| XLOC_049369 | 0 | 0 | 3 | 0 | - | multiexon |
| XLOC_014992 | 0 | 0 | 2 | 3 | + | singleexon |
| XLOC_060286 | 1 | 0 | 2 | 2 | - | multiexon |
| XLOC_063277 | 0 | 0 | 24 | 0 | - | multiexon |
| XLOC_009801 | 0 | 0 | 5 | 0 | - | singleexon |
| XLOC_014934 | 0 | 0 | 0 | 5 | + | singleexon |
| XLOC_039154 | 0 | 0 | 15 | 0 | - | singleexon |
| XLOC_014085 | 2 | 0 | 1 | 2 | - | singleexon |
| XLOC_024930 | 0 | 0 | 9 | 0 | - | singleexon |
| XLOC_046978 | 0 | 0 | 0 | 3 | + | singleexon |
| XLOC_028528 | 0 | 0 | 9 | 0 | - | singleexon |
| XLOC_017827 | 0 | 0 | 7 | 0 | - | singleexon |
| XLOC_039465 | 0 | 0 | 1 | 6 | + | singleexon |
| XLOC_034509 | 0 | 6 | 1 | 0 | + | singleexon |
| XLOC_039491 | 0 | 0 | 3 | 0 | - | multiexon |
| XLOC_028972 | 0 | 4 | 0 | 3 | + | singleexon |
| XLOC_031400 | 0 | 0 | 0 | 5 | + | singleexon |
| XLOC_046447 | 0 | 0 | 3 | 0 | - | singleexon |
| XLOC_057112 | 0 | 0 | 0 | 3 | + | singleexon |
| XLOC_057305 | 0 | 0 | 7 | 0 | - | multiexon |
| XLOC_016276 | 0 | 0 | 11 | 0 | - | singleexon |
| XLOC_026141 | 0 | 0 | 14 | 99 | + | singleexon |
| XLOC_053243 | 0 | 0 | 22 | 0 | - | singleexon |
| XLOC_047731 | 0 | 1 | 6 | 0 | - | singleexon |
| XLOC_006015 | 0 | 4 | 0 | 5 | + | singleexon |
| XLOC_044336 | 0 | 0 | 7 | 0 | - | singleexon |
| XLOC_029592 | 0 | 0 | 18 | 0 | - | singleexon |
| XLOC_008507 | 0 | 0 | 17 | 0 | - | singleexon |
| XLOC_042253 | 0 | 0 | 3 | 0 | - | multiexon |
| XLOC_016316 | 0 | 0 | 3 | 0 | - | singleexon |
| XLOC_028866 | 1 | 0 | 0 | 6 | + | singleexon |
| XLOC_019232 | 0 | 0 | 11 | 0 | - | singleexon |
| XLOC_056138 | 1 | 1 | 48 | 1 | - | multiexon |
| XLOC_022680 | 0 | 0 | 5 | 0 | - | singleexon |
| XLOC_018852 | 0 | 0 | 0 | 6 | + | singleexon |
| XLOC_025776 | 0 | 0 | 4 | 0 | - | singleexon |
| XLOC_002630 | 0 | 0 | 5 | 0 | - | singleexon |
| XLOC_049424 | 0 | 0 | 0 | 6 | + | singleexon |
| XLOC_040165 | 0 | 2 | 7 | 0 | - | singleexon |
| XLOC_043623 | 11 | 0 | 0 | 0 | - | singleexon |
| XLOC_003606 | 0 | 1 | 0 | 2 | + | singleexon |
| XLOC_051640 | 0 | 0 | 5 | 0 | - | singleexon |
| XLOC_018563 | 0 | 0 | 0 | 6 | + | singleexon |
| XLOC_062291 | 0 | 0 | 0 | 3 | + | singleexon |
| XLOC_064286 | 1 | 0 | 7 | 0 | - | multiexon |
| XLOC_018766 | 0 | 0 | 2 | 3 | + | singleexon |
| XLOC_038430 | 1 | 4 | 6 | 0 | - | singleexon |
| XLOC_027735 | 0 | 0 | 3 | 1 | - | singleexon |
| XLOC_016916 | 0 | 0 | 0 | 18 | + | singleexon |
| XLOC_009009 | 0 | 0 | 0 | 4 | + | multiexon |
| XLOC_059012 | 0 | 0 | 5 | 0 | - | singleexon |
| XLOC_005187 | 0 | 0 | 0 | 10 | + | singleexon |
| XLOC_022033 | 0 | 0 | 19 | 0 | - | singleexon |
| XLOC_034410 | 0 | 0 | 3 | 0 | - | multiexon |
| XLOC_058479 | 0 | 0 | 3 | 1 | - | multiexon |
| XLOC_060436 | 1 | 0 | 2 | 0 | - | multiexon |
| XLOC_043285 | 0 | 5 | 0 | 43 | + | singleexon |
| XLOC_034888 | 1 | 0 | 7 | 0 | - | multiexon |
| XLOC_018076 | 0 | 0 | 0 | 3 | + | singleexon |
| XLOC_013521 | 1 | 0 | 6 | 0 | - | singleexon |
| XLOC_025188 | 0 | 0 | 0 | 6 | + | multiexon |
| XLOC_000397 | 2 | 1 | 2 | 1 | - | singleexon |
| XLOC_050828 | 10 | 0 | 5 | 2 | - | multiexon |
| XLOC_027489 | 0 | 1 | 0 | 3 | + | singleexon |
| XLOC_026276 | 0 | 0 | 7 | 1 | - | singleexon |
| XLOC_015257 | 0 | 0 | 0 | 8 | + | singleexon |
| XLOC_005237 | 0 | 0 | 0 | 5 | + | singleexon |
| XLOC_036977 | 0 | 0 | 3 | 1 | - | singleexon |
| XLOC_051426 | 0 | 0 | 3 | 0 | - | singleexon |
| XLOC_040657 | 0 | 0 | 5 | 0 | - | singleexon |
| XLOC_033109 | 0 | 0 | 2 | 4 | + | singleexon |
| XLOC_032642 | 3 | 0 | 0 | 1 | - | singleexon |
| XLOC_030465 | 0 | 0 | 0 | 3 | + | singleexon |
| XLOC_044143 | 0 | 0 | 5 | 5 | - | singleexon |
| XLOC_039666 | 1 | 0 | 2 | 0 | - | multiexon |
| XLOC_018591 | 2 | 0 | 25 | 0 | - | singleexon |
| XLOC_025218 | 0 | 0 | 0 | 3 | + | singleexon |
| XLOC_014972 | 0 | 0 | 0 | 11 | + | singleexon |
| XLOC_030957 | 0 | 0 | 14 | 0 | - | singleexon |
| XLOC_049374 | 0 | 0 | 1 | 5 | + | singleexon |
| XLOC_001855 | 0 | 0 | 4 | 0 | - | singleexon |
| XLOC_014051 | 3 | 0 | 1 | 0 | - | singleexon |
| XLOC_047919 | 0 | 0 | 3 | 0 | - | singleexon |
| XLOC_020610 | 0 | 0 | 3 | 0 | - | singleexon |
| XLOC_037069 | 0 | 0 | 7 | 0 | - | singleexon |
| XLOC_062919 | 0 | 1 | 1 | 5 | + | singleexon |
| XLOC_028791 | 0 | 0 | 6 | 0 | - | singleexon |
| XLOC_027892 | 1 | 1 | 2 | 0 | - | singleexon |
| XLOC_011878 | 0 | 0 | 4 | 1 | - | singleexon |
| XLOC_041025 | 0 | 1 | 4 | 0 | - | singleexon |
| XLOC_034125 | 0 | 4 | 1 | 0 | + | singleexon |
| XLOC_055898 | 11 | 0 | 0 | 0 | - | multiexon |
| XLOC_001051 | 0 | 1 | 0 | 6 | + | singleexon |
| XLOC_043895 | 0 | 0 | 1 | 3 | + | singleexon |
| XLOC_034934 | 0 | 0 | 0 | 13 | + | singleexon |
| XLOC_033126 | 0 | 1 | 0 | 16 | + | multiexon |
| XLOC_029819 | 0 | 0 | 0 | 17 | + | singleexon |
| XLOC_029886 | 0 | 1 | 9 | 6 | - | singleexon |
| XLOC_060222 | 0 | 0 | 5 | 0 | - | multiexon |
| XLOC_015007 | 0 | 0 | 0 | 6 | + | singleexon |
| XLOC_006733 | 0 | 0 | 5 | 1 | - | singleexon |
| XLOC_043869 | 0 | 0 | 22 | 0 | - | singleexon |
| XLOC_031576 | 0 | 0 | 0 | 3 | + | singleexon |
| XLOC_030238 | 0 | 0 | 3 | 0 | - | singleexon |
| XLOC_053297 | 0 | 0 | 6 | 0 | - | singleexon |
| XLOC_024313 | 0 | 0 | 0 | 4 | + | multiexon |
| XLOC_030751 | 0 | 0 | 22 | 0 | - | singleexon |
| XLOC_049751 | 0 | 1 | 0 | 2 | + | singleexon |
| XLOC_062443 | 15 | 0 | 6 | 1 | - | singleexon |
| XLOC_054430 | 12 | 0 | 0 | 0 | - | singleexon |
| XLOC_017925 | 0 | 0 | 5 | 0 | - | singleexon |
| XLOC_028704 | 0 | 0 | 0 | 4 | + | multiexon |
| XLOC_029824 | 0 | 0 | 0 | 10 | + | multiexon |
| XLOC_007737 | 0 | 0 | 6 | 0 | - | singleexon |
| XLOC_008557 | 0 | 0 | 3 | 1 | - | singleexon |
| XLOC_024604 | 0 | 1 | 6 | 41 | + | singleexon |
| XLOC_056835 | 0 | 0 | 10 | 0 | - | singleexon |
| XLOC_038202 | 1 | 0 | 3 | 0 | - | singleexon |
| XLOC_058284 | 0 | 3 | 1 | 1 | + | singleexon |
| XLOC_010249 | 0 | 0 | 0 | 24 | + | singleexon |
| XLOC_036865 | 0 | 0 | 3 | 0 | - | singleexon |
| XLOC_000180 | 0 | 1 | 1 | 5 | + | singleexon |
| XLOC_020771 | 0 | 0 | 3 | 87 | + | singleexon |
| XLOC_035116 | 1 | 0 | 6 | 0 | - | multiexon |
| XLOC_058726 | 1 | 0 | 2 | 0 | - | singleexon |
| XLOC_031105 | 0 | 1 | 0 | 4 | + | singleexon |
| XLOC_040049 | 1 | 1 | 3 | 0 | - | singleexon |
| XLOC_029591 | 0 | 1 | 15 | 0 | - | singleexon |
| XLOC_032955 | 0 | 1 | 112 | 0 | - | singleexon |
| XLOC_015741 | 0 | 1 | 0 | 4 | + | singleexon |
| XLOC_037975 | 0 | 0 | 0 | 10 | + | singleexon |
| XLOC_055511 | 0 | 2 | 0 | 8 | + | singleexon |
| XLOC_047628 | 1 | 0 | 5 | 0 | - | singleexon |
| XLOC_047091 | 0 | 1 | 0 | 2 | + | multiexon |
| XLOC_048914 | 0 | 0 | 0 | 10 | + | singleexon |
| XLOC_014076 | 1 | 0 | 3 | 1 | - | singleexon |
| XLOC_031258 | 1 | 0 | 1 | 3 | + | singleexon |
| XLOC_033224 | 0 | 0 | 0 | 234 | + | singleexon |
| XLOC_061231 | 0 | 0 | 3 | 0 | - | multiexon |
| XLOC_018460 | 0 | 0 | 0 | 14 | + | singleexon |
| XLOC_024882 | 0 | 0 | 6 | 0 | - | singleexon |
| XLOC_007847 | 0 | 0 | 0 | 3 | + | singleexon |
| XLOC_048547 | 0 | 0 | 0 | 4 | + | singleexon |
| XLOC_013626 | 0 | 0 | 5 | 1 | - | singleexon |
| XLOC_057125 | 0 | 0 | 0 | 5 | + | singleexon |
| XLOC_043355 | 0 | 0 | 0 | 44 | + | singleexon |
| XLOC_024782 | 0 | 0 | 3 | 0 | - | singleexon |
| XLOC_037008 | 2 | 0 | 8 | 0 | - | singleexon |
| XLOC_004116 | 0 | 0 | 0 | 5 | + | singleexon |
| XLOC_035246 | 0 | 0 | 3 | 1 | - | singleexon |
| XLOC_014962 | 1 | 0 | 0 | 18 | + | singleexon |
| XLOC_023576 | 0 | 0 | 0 | 3 | + | singleexon |
| XLOC_017360 | 0 | 0 | 0 | 58 | + | singleexon |
| XLOC_002324 | 3 | 1 | 94 | 0 | - | singleexon |
| XLOC_034518 | 1 | 2 | 1 | 5 | + | multiexon |
| XLOC_045423 | 0 | 0 | 7 | 0 | - | multiexon |
| XLOC_040646 | 1 | 0 | 5 | 0 | - | singleexon |
| XLOC_033112 | 0 | 1 | 0 | 2 | + | singleexon |
| XLOC_002851 | 0 | 0 | 0 | 4 | + | singleexon |
| XLOC_034688 | 0 | 0 | 7 | 1 | - | singleexon |
| XLOC_007800 | 1 | 0 | 32 | 6 | - | singleexon |
| XLOC_017281 | 0 | 0 | 11 | 0 | - | singleexon |
| XLOC_015974 | 0 | 0 | 0 | 3 | + | singleexon |
| XLOC_011450 | 0 | 0 | 3 | 0 | - | singleexon |
| XLOC_015670 | 0 | 0 | 5 | 0 | - | singleexon |
| XLOC_028234 | 0 | 0 | 6 | 1 | - | singleexon |
| XLOC_061896 | 0 | 0 | 6 | 0 | - | singleexon |
| XLOC_027447 | 0 | 0 | 7 | 13 | + | singleexon |
| XLOC_062354 | 0 | 0 | 0 | 3 | + | singleexon |
| XLOC_009454 | 0 | 0 | 0 | 42 | + | singleexon |
| XLOC_054961 | 0 | 0 | 11 | 2 | - | singleexon |
| XLOC_053064 | 0 | 2 | 4 | 0 | - | singleexon |
| XLOC_045327 | 0 | 0 | 1 | 4 | + | multiexon |
| XLOC_043159 | 0 | 0 | 7 | 0 | - | multiexon |
| XLOC_028300 | 0 | 0 | 0 | 3 | + | singleexon |
| XLOC_055194 | 0 | 0 | 0 | 3 | + | singleexon |
| XLOC_010644 | 0 | 0 | 0 | 10 | + | singleexon |
| XLOC_014810 | 0 | 0 | 12 | 0 | - | singleexon |
| XLOC_032078 | 1 | 0 | 2 | 0 | - | singleexon |
| XLOC_007105 | 0 | 2 | 285 | 2 | - | multiexon |
| XLOC_015003 | 0 | 0 | 1 | 18 | + | singleexon |
| XLOC_037907 | 0 | 1 | 3 | 0 | - | singleexon |
| XLOC_025043 | 0 | 9 | 0 | 1 | + | singleexon |
| XLOC_025483 | 0 | 1 | 2 | 7 | + | multiexon |
| XLOC_046347 | 0 | 0 | 9 | 25 | + | singleexon |
| XLOC_013117 | 0 | 0 | 1 | 6 | + | singleexon |
| XLOC_043575 | 1 | 0 | 2 | 0 | - | multiexon |
| XLOC_039689 | 5 | 250 | 1 | 0 | + | multiexon |
| XLOC_059169 | 2 | 7 | 70 | 45 | - | multiexon |
| XLOC_005162 | 0 | 0 | 0 | 3 | + | singleexon |
| XLOC_027275 | 4 | 0 | 0 | 3 | - | singleexon |
| XLOC_008878 | 0 | 2 | 0 | 19 | + | singleexon |
| XLOC_012635 | 0 | 0 | 3 | 0 | - | singleexon |
| XLOC_021584 | 0 | 0 | 3 | 2 | - | singleexon |
| XLOC_015990 | 0 | 0 | 12 | 0 | - | multiexon |
| XLOC_027457 | 0 | 1 | 5 | 0 | - | singleexon |
| XLOC_018362 | 0 | 0 | 0 | 9 | + | singleexon |
| XLOC_048384 | 0 | 0 | 17 | 0 | - | singleexon |
| XLOC_017093 | 0 | 0 | 56 | 0 | - | singleexon |
| XLOC_054535 | 0 | 0 | 2 | 5 | + | singleexon |
| XLOC_026484 | 1 | 1 | 72 | 2 | - | singleexon |
| XLOC_005963 | 0 | 0 | 3 | 3 | - | multiexon |
| XLOC_038515 | 0 | 0 | 0 | 4 | + | singleexon |
| XLOC_024748 | 0 | 0 | 7 | 0 | - | singleexon |
| XLOC_029138 | 1 | 0 | 12 | 3 | - | multiexon |
| XLOC_020196 | 0 | 0 | 7 | 0 | - | singleexon |
| XLOC_020793 | 0 | 0 | 3 | 1 | - | singleexon |
| XLOC_000396 | 0 | 0 | 5 | 9 | + | singleexon |
| XLOC_024464 | 0 | 0 | 5 | 0 | - | singleexon |
| XLOC_042140 | 0 | 0 | 7 | 2 | - | singleexon |
| XLOC_028680 | 0 | 0 | 0 | 3 | + | singleexon |
| XLOC_054551 | 0 | 0 | 9 | 0 | - | multiexon |
| XLOC_049645 | 0 | 0 | 0 | 3 | + | singleexon |
| XLOC_005248 | 0 | 0 | 1 | 14 | + | singleexon |
| XLOC_022584 | 0 | 0 | 13 | 0 | - | singleexon |
| XLOC_023952 | 2 | 0 | 1 | 0 | - | singleexon |
| XLOC_008579 | 0 | 0 | 3 | 0 | - | singleexon |
| XLOC_033825 | 4 | 0 | 0 | 1 | - | singleexon |
| XLOC_013967 | 0 | 1 | 3 | 1 | - | singleexon |
| XLOC_045167 | 1 | 0 | 0 | 8 | + | multiexon |
| XLOC_000249 | 5 | 0 | 3 | 1 | - | singleexon |
| XLOC_003907 | 0 | 0 | 1 | 5 | + | singleexon |
| XLOC_003727 | 0 | 0 | 3 | 0 | - | singleexon |
| XLOC_040064 | 0 | 4 | 12 | 0 | - | singleexon |
| XLOC_043653 | 0 | 0 | 11 | 0 | - | singleexon |
| XLOC_064122 | 0 | 1 | 8 | 1 | - | multiexon |
| XLOC_024659 | 0 | 0 | 7 | 0 | - | singleexon |
| XLOC_031657 | 1 | 1 | 9 | 0 | - | singleexon |
| XLOC_035280 | 0 | 2 | 0 | 1 | + | singleexon |
| XLOC_061069 | 0 | 0 | 2 | 3 | + | singleexon |
| XLOC_040113 | 0 | 0 | 3 | 0 | - | singleexon |
| XLOC_023976 | 0 | 1 | 5 | 0 | - | singleexon |
| XLOC_013161 | 0 | 6 | 0 | 25 | + | multiexon |
| XLOC_037015 | 0 | 0 | 4 | 2 | - | singleexon |
| XLOC_054770 | 0 | 0 | 3 | 1 | - | singleexon |
| XLOC_032727 | 0 | 0 | 11 | 2 | - | singleexon |
| XLOC_037911 | 0 | 0 | 12 | 0 | - | singleexon |
| XLOC_020520 | 0 | 0 | 3 | 0 | - | singleexon |
| XLOC_009848 | 3 | 0 | 11 | 0 | - | multiexon |
| XLOC_048391 | 0 | 0 | 17 | 0 | - | singleexon |
| XLOC_034900 | 0 | 4 | 0 | 0 | + | singleexon |
| XLOC_009559 | 0 | 0 | 0 | 4 | + | singleexon |
| XLOC_009374 | 1 | 0 | 3 | 0 | - | singleexon |
| XLOC_028814 | 0 | 1 | 0 | 2 | + | singleexon |
| XLOC_023291 | 0 | 0 | 1 | 10 | + | singleexon |
| XLOC_062407 | 0 | 0 | 2 | 6 | + | multiexon |
| XLOC_046105 | 0 | 0 | 0 | 3 | + | singleexon |
| XLOC_025838 | 0 | 0 | 6 | 0 | - | singleexon |
| XLOC_029847 | 4 | 0 | 0 | 0 | - | singleexon |
| XLOC_054565 | 0 | 0 | 0 | 3 | + | singleexon |
| XLOC_046356 | 0 | 0 | 0 | 3 | + | singleexon |
| XLOC_015353 | 0 | 0 | 2 | 3 | + | singleexon |
| XLOC_013466 | 0 | 0 | 3 | 0 | - | singleexon |
| XLOC_053617 | 0 | 0 | 0 | 11 | + | singleexon |
| XLOC_027091 | 1 | 0 | 3 | 0 | - | singleexon |
| XLOC_015352 | 1 | 0 | 0 | 9 | + | singleexon |
| XLOC_020164 | 0 | 0 | 6 | 0 | - | singleexon |
| XLOC_060050 | 0 | 0 | 0 | 3 | + | multiexon |
| XLOC_034207 | 0 | 3 | 0 | 0 | + | multiexon |
| XLOC_009772 | 0 | 0 | 10 | 0 | - | singleexon |
| XLOC_003469 | 0 | 0 | 0 | 3 | + | singleexon |
| XLOC_058116 | 0 | 0 | 3 | 0 | - | singleexon |
| XLOC_052129 | 0 | 0 | 25 | 6 | - | singleexon |
| XLOC_031771 | 0 | 0 | 0 | 3 | + | singleexon |
| XLOC_050520 | 0 | 0 | 0 | 3 | + | singleexon |
| XLOC_037528 | 0 | 0 | 5 | 0 | - | singleexon |
| XLOC_060307 | 0 | 1 | 0 | 5 | + | multiexon |
| XLOC_052155 | 1 | 0 | 30 | 0 | - | multiexon |
| XLOC_040533 | 0 | 0 | 3 | 2 | - | singleexon |
| XLOC_015749 | 1 | 0 | 21 | 0 | - | singleexon |
| XLOC_062468 | 1 | 2 | 3 | 0 | - | singleexon |
| XLOC_003798 | 0 | 0 | 0 | 20 | + | multiexon |
| XLOC_037403 | 5 | 0 | 0 | 0 | - | singleexon |
| XLOC_005190 | 0 | 0 | 0 | 4 | + | singleexon |
| XLOC_039882 | 0 | 0 | 3 | 0 | - | singleexon |
| XLOC_021851 | 0 | 2 | 0 | 1 | + | singleexon |
| XLOC_052824 | 0 | 3 | 0 | 1 | + | singleexon |
| XLOC_049831 | 0 | 0 | 5 | 0 | - | multiexon |
| XLOC_036017 | 0 | 2 | 1 | 1 | + | singleexon |
| XLOC_029776 | 0 | 0 | 0 | 80 | + | multiexon |
| XLOC_006784 | 0 | 1 | 0 | 2 | + | singleexon |
| XLOC_031410 | 0 | 0 | 0 | 4 | + | singleexon |
| XLOC_009647 | 1 | 0 | 2 | 0 | - | singleexon |
| XLOC_006181 | 0 | 0 | 0 | 6 | + | singleexon |
| XLOC_050746 | 2 | 0 | 4 | 0 | - | multiexon |
| XLOC_040114 | 0 | 1 | 0 | 30 | + | singleexon |
| XLOC_031822 | 2 | 0 | 11 | 0 | - | singleexon |
| XLOC_007480 | 0 | 0 | 0 | 10 | + | singleexon |
| XLOC_050166 | 0 | 0 | 14 | 0 | - | singleexon |
| XLOC_053084 | 0 | 0 | 26 | 1 | - | singleexon |
| XLOC_053055 | 0 | 4 | 0 | 0 | + | singleexon |
| XLOC_038217 | 0 | 0 | 0 | 3 | + | singleexon |
| XLOC_063215 | 0 | 0 | 4 | 0 | - | singleexon |
| XLOC_052977 | 0 | 0 | 0 | 4 | + | singleexon |
| XLOC_008320 | 0 | 0 | 9 | 1 | - | multiexon |
| XLOC_054942 | 1 | 0 | 8 | 0 | - | singleexon |
| XLOC_007425 | 0 | 0 | 0 | 17 | + | singleexon |
| XLOC_006900 | 0 | 0 | 3 | 1 | - | singleexon |
| XLOC_014796 | 0 | 0 | 13 | 0 | - | singleexon |
| XLOC_040652 | 0 | 0 | 3 | 1 | - | singleexon |
| XLOC_014529 | 0 | 0 | 0 | 6 | + | singleexon |
| XLOC_057461 | 0 | 0 | 9 | 22 | + | singleexon |
| XLOC_039871 | 0 | 0 | 6 | 0 | - | singleexon |
| XLOC_063342 | 0 | 4 | 0 | 2 | + | multiexon |
| XLOC_016025 | 0 | 0 | 7 | 0 | - | multiexon |
| XLOC_039844 | 0 | 0 | 3 | 0 | - | singleexon |
| XLOC_044898 | 0 | 0 | 14 | 0 | - | singleexon |
| XLOC_056921 | 0 | 0 | 0 | 5 | + | singleexon |
| XLOC_030232 | 0 | 0 | 0 | 6 | + | singleexon |
| XLOC_054514 | 0 | 0 | 15 | 0 | - | multiexon |
| XLOC_006080 | 0 | 0 | 0 | 6 | + | singleexon |
| XLOC_019538 | 0 | 0 | 0 | 9 | + | singleexon |
| XLOC_009850 | 0 | 1 | 7 | 0 | - | multiexon |
| XLOC_049473 | 0 | 0 | 16 | 0 | - | singleexon |
| XLOC_049408 | 0 | 0 | 0 | 7 | + | singleexon |
| XLOC_058757 | 1 | 0 | 3 | 0 | - | singleexon |
| XLOC_041691 | 0 | 0 | 3 | 0 | - | singleexon |
| XLOC_027942 | 0 | 0 | 4 | 0 | - | singleexon |
| XLOC_027491 | 3 | 0 | 0 | 12 | + | singleexon |
| XLOC_049924 | 4 | 0 | 3 | 0 | - | singleexon |
| XLOC_001289 | 0 | 3 | 0 | 87 | + | multiexon |
| XLOC_034927 | 0 | 0 | 0 | 7 | + | singleexon |
| XLOC_008319 | 1 | 0 | 2 | 1 | - | multiexon |
| XLOC_051701 | 9 | 29 | 9 | 0 | + | multiexon |
| XLOC_063349 | 1 | 0 | 3 | 0 | - | singleexon |
| XLOC_049189 | 0 | 0 | 0 | 8 | + | singleexon |
| XLOC_028340 | 0 | 0 | 6 | 1 | - | singleexon |
| XLOC_014185 | 0 | 0 | 0 | 5 | + | singleexon |
| XLOC_013179 | 0 | 0 | 2 | 3 | + | singleexon |
| XLOC_020427 | 0 | 0 | 0 | 4 | + | singleexon |
| XLOC_033123 | 0 | 0 | 1 | 17 | + | singleexon |
| XLOC_019695 | 0 | 1 | 21 | 2 | - | singleexon |
| XLOC_039893 | 0 | 1 | 12 | 0 | - | singleexon |
| XLOC_027279 | 3 | 0 | 1 | 0 | - | singleexon |
| XLOC_038423 | 0 | 0 | 0 | 23 | + | singleexon |
| XLOC_047210 | 3 | 0 | 1 | 19 | + | singleexon |
| XLOC_036411 | 1 | 0 | 7 | 0 | - | multiexon |
| XLOC_045400 | 0 | 2 | 10 | 0 | - | multiexon |
| XLOC_038480 | 0 | 0 | 0 | 4 | + | singleexon |
| XLOC_013332 | 0 | 0 | 2 | 37 | + | singleexon |
| XLOC_053362 | 0 | 0 | 18 | 2 | - | singleexon |
| XLOC_053566 | 0 | 0 | 3 | 0 | - | singleexon |
| XLOC_041487 | 0 | 0 | 1 | 3 | + | multiexon |
| XLOC_051088 | 0 | 1 | 0 | 2 | + | singleexon |
| XLOC_009867 | 0 | 0 | 3 | 0 | - | singleexon |
| XLOC_042116 | 1 | 0 | 22 | 1 | - | singleexon |
| XLOC_047793 | 1 | 0 | 1 | 7 | + | singleexon |
| XLOC_033916 | 2 | 1 | 3 | 0 | - | singleexon |
| XLOC_006621 | 1 | 0 | 4 | 0 | - | singleexon |
| XLOC_048347 | 0 | 0 | 3 | 0 | - | singleexon |
| XLOC_020883 | 0 | 0 | 3 | 0 | - | singleexon |
| XLOC_029831 | 0 | 0 | 0 | 3 | + | singleexon |
| XLOC_009217 | 0 | 0 | 3 | 0 | - | singleexon |
| XLOC_017842 | 0 | 0 | 8 | 0 | - | singleexon |
| XLOC_055737 | 0 | 0 | 10 | 0 | - | singleexon |
| XLOC_041182 | 0 | 0 | 6 | 3 | - | multiexon |
| XLOC_036684 | 0 | 0 | 24 | 2 | - | multiexon |
| XLOC_046691 | 2 | 0 | 5 | 5 | - | singleexon |
| XLOC_007424 | 0 | 0 | 0 | 4 | + | singleexon |
| XLOC_018040 | 0 | 0 | 3 | 0 | - | singleexon |
| XLOC_050139 | 2 | 0 | 33 | 0 | - | singleexon |
| XLOC_013821 | 0 | 0 | 6 | 0 | - | singleexon |
| XLOC_017892 | 0 | 0 | 7 | 0 | - | multiexon |
| XLOC_051216 | 0 | 0 | 33 | 0 | - | singleexon |
| XLOC_025610 | 0 | 0 | 0 | 11 | + | singleexon |
| XLOC_010315 | 0 | 0 | 0 | 3 | + | singleexon |
| XLOC_048366 | 0 | 0 | 3 | 1 | - | singleexon |
| XLOC_003144 | 0 | 0 | 5 | 1 | - | singleexon |
| XLOC_003674 | 0 | 0 | 0 | 4 | + | singleexon |
| XLOC_041513 | 2 | 0 | 1 | 0 | - | multiexon |
| XLOC_029226 | 0 | 0 | 0 | 3 | + | singleexon |
| XLOC_042386 | 0 | 0 | 3 | 0 | - | singleexon |
| XLOC_020291 | 1 | 0 | 2 | 0 | - | singleexon |
| XLOC_017521 | 0 | 0 | 0 | 3 | + | singleexon |
| XLOC_026139 | 0 | 2 | 3 | 3 | - | singleexon |
| XLOC_032133 | 0 | 0 | 0 | 11 | + | singleexon |
| XLOC_055775 | 0 | 0 | 7 | 0 | - | singleexon |
| XLOC_036817 | 0 | 0 | 61 | 3 | - | singleexon |
| XLOC_035325 | 0 | 0 | 0 | 5 | + | singleexon |
| XLOC_012623 | 0 | 0 | 0 | 8 | + | singleexon |
| XLOC_031457 | 0 | 0 | 5 | 3 | - | multiexon |
| XLOC_038553 | 4 | 0 | 3 | 2 | - | singleexon |
| XLOC_048907 | 0 | 0 | 5 | 0 | - | singleexon |
| XLOC_049204 | 0 | 0 | 0 | 3 | + | singleexon |
| XLOC_056804 | 0 | 0 | 25 | 1 | - | singleexon |
| XLOC_043523 | 0 | 0 | 4 | 0 | - | singleexon |
| XLOC_053418 | 0 | 0 | 11 | 0 | - | singleexon |
| XLOC_041751 | 0 | 0 | 6 | 2 | - | singleexon |
| XLOC_037910 | 0 | 3 | 4 | 1 | - | singleexon |
| XLOC_028505 | 0 | 1 | 0 | 4 | + | singleexon |
| XLOC_007223 | 0 | 0 | 5 | 0 | - | singleexon |
| XLOC_023975 | 0 | 0 | 1 | 3 | + | singleexon |
| XLOC_018270 | 1 | 0 | 7 | 22 | + | multiexon |
| XLOC_059836 | 0 | 0 | 0 | 14 | + | singleexon |
| XLOC_019668 | 0 | 0 | 0 | 4 | + | singleexon |
| XLOC_046976 | 0 | 0 | 0 | 29 | + | singleexon |
| XLOC_007722 | 0 | 4 | 2 | 1 | + | singleexon |
| XLOC_020903 | 0 | 0 | 4 | 0 | - | singleexon |
| XLOC_009332 | 0 | 0 | 4 | 0 | - | singleexon |
| XLOC_033787 | 0 | 0 | 42 | 1 | - | singleexon |
| XLOC_039469 | 0 | 0 | 0 | 9 | + | singleexon |
| XLOC_063617 | 0 | 0 | 2 | 6 | + | singleexon |
| XLOC_060149 | 0 | 0 | 4 | 0 | - | singleexon |
| XLOC_061900 | 0 | 0 | 4 | 0 | - | singleexon |
| XLOC_047007 | 0 | 2 | 0 | 3 | + | singleexon |
| XLOC_028900 | 1 | 0 | 3 | 1 | - | singleexon |
| XLOC_035088 | 0 | 0 | 12 | 0 | - | multiexon |
| XLOC_030990 | 2 | 0 | 2 | 0 | - | singleexon |
| XLOC_026205 | 0 | 2 | 0 | 6 | + | multiexon |
| XLOC_001007 | 1 | 0 | 1 | 20 | + | singleexon |
| XLOC_033576 | 0 | 0 | 3 | 1 | - | multiexon |
| XLOC_005427 | 0 | 0 | 4 | 1 | - | singleexon |
| XLOC_012329 | 0 | 0 | 0 | 3 | + | multiexon |
| XLOC_000501 | 0 | 0 | 11 | 0 | - | multiexon |
| XLOC_037463 | 0 | 0 | 4 | 3 | - | singleexon |
| XLOC_060387 | 0 | 1 | 0 | 6 | + | singleexon |
| XLOC_008510 | 1 | 0 | 5 | 0 | - | singleexon |
| XLOC_053421 | 0 | 3 | 26 | 0 | - | singleexon |
| XLOC_054427 | 0 | 0 | 2 | 5 | + | singleexon |
| XLOC_029470 | 0 | 1 | 10 | 0 | - | singleexon |
| XLOC_055108 | 0 | 0 | 0 | 3 | + | singleexon |
| XLOC_007393 | 0 | 0 | 0 | 5 | + | singleexon |
| XLOC_059908 | 0 | 0 | 0 | 3 | + | singleexon |
| XLOC_028868 | 0 | 0 | 22 | 1 | - | singleexon |
| XLOC_020732 | 0 | 0 | 2 | 6 | + | singleexon |
| XLOC_016003 | 0 | 0 | 6 | 0 | - | singleexon |
| XLOC_034926 | 0 | 0 | 0 | 5 | + | singleexon |
| XLOC_061860 | 0 | 0 | 3 | 0 | - | singleexon |
| XLOC_032812 | 0 | 0 | 0 | 21 | + | multiexon |
| XLOC_010267 | 0 | 0 | 0 | 9 | + | singleexon |
| XLOC_062056 | 0 | 1 | 0 | 2 | + | singleexon |
| XLOC_032759 | 0 | 0 | 0 | 5 | + | singleexon |
| XLOC_063683 | 0 | 0 | 0 | 4 | + | singleexon |
| XLOC_002820 | 0 | 1 | 1 | 2 | + | singleexon |
| XLOC_021852 | 2 | 0 | 11 | 0 | - | singleexon |
| XLOC_061420 | 0 | 0 | 3 | 0 | - | singleexon |
| XLOC_051691 | 2 | 0 | 79 | 0 | - | singleexon |
| XLOC_040200 | 0 | 0 | 6 | 0 | - | singleexon |
| XLOC_042936 | 0 | 0 | 6 | 2 | - | singleexon |
| XLOC_011694 | 0 | 0 | 7 | 0 | - | multiexon |
| XLOC_012181 | 4 | 0 | 0 | 0 | - | singleexon |
| XLOC_037217 | 0 | 0 | 3 | 0 | - | singleexon |
| XLOC_061929 | 0 | 0 | 11 | 0 | - | multiexon |
| XLOC_015508 | 0 | 0 | 0 | 5 | + | singleexon |
| XLOC_034272 | 1 | 3 | 0 | 0 | + | singleexon |
| XLOC_052213 | 0 | 0 | 3 | 0 | - | singleexon |
| XLOC_017250 | 1 | 0 | 2 | 0 | - | singleexon |
| XLOC_039634 | 10 | 0 | 3 | 20 | + | singleexon |
| XLOC_038808 | 0 | 0 | 3 | 0 | - | singleexon |
| XLOC_006888 | 0 | 0 | 7 | 0 | - | singleexon |
| XLOC_035042 | 0 | 0 | 1 | 86 | + | multiexon |
| XLOC_048181 | 0 | 0 | 0 | 3 | + | singleexon |
| XLOC_055989 | 0 | 0 | 7 | 2 | - | multiexon |
| XLOC_059036 | 1 | 0 | 0 | 4 | + | singleexon |
| XLOC_050612 | 0 | 0 | 0 | 3 | + | singleexon |
| XLOC_014385 | 0 | 0 | 67 | 0 | - | singleexon |
| XLOC_059299 | 1 | 0 | 31 | 0 | - | singleexon |
| XLOC_028256 | 0 | 0 | 4 | 0 | - | singleexon |
| XLOC_051607 | 2 | 1 | 16 | 2 | - | singleexon |
| XLOC_035462 | 0 | 0 | 5 | 1 | - | singleexon |
| XLOC_017603 | 7 | 0 | 0 | 0 | - | singleexon |
| XLOC_026020 | 0 | 0 | 2 | 28 | + | singleexon |
| XLOC_050368 | 1 | 0 | 53 | 1 | - | singleexon |
| XLOC_057118 | 0 | 0 | 0 | 6 | + | singleexon |
| XLOC_037381 | 0 | 0 | 0 | 3 | + | singleexon |
| XLOC_033615 | 0 | 0 | 7 | 0 | - | singleexon |
| XLOC_049967 | 0 | 0 | 0 | 3 | + | singleexon |
| XLOC_013650 | 2 | 2 | 1 | 0 | - | singleexon |
| XLOC_025826 | 0 | 0 | 4 | 0 | - | singleexon |
| XLOC_014921 | 0 | 0 | 3 | 0 | - | singleexon |
| XLOC_013578 | 0 | 0 | 3 | 0 | - | singleexon |
| XLOC_014805 | 0 | 0 | 3 | 4 | + | singleexon |
| XLOC_028545 | 0 | 2 | 2 | 4 | + | singleexon |
| XLOC_017352 | 0 | 2 | 0 | 74 | + | singleexon |
| XLOC_041733 | 0 | 2 | 0 | 3 | + | multiexon |
| XLOC_064580 | 0 | 1 | 0 | 2 | + | singleexon |
| XLOC_001309 | 0 | 0 | 1 | 4 | + | singleexon |
| XLOC_030468 | 0 | 0 | 0 | 8 | + | singleexon |
| XLOC_026847 | 0 | 1 | 1 | 5 | + | singleexon |
| XLOC_058633 | 0 | 1 | 0 | 8 | + | multiexon |
| XLOC_030204 | 1 | 0 | 9 | 0 | - | singleexon |
| XLOC_005956 | 0 | 0 | 4 | 3 | - | singleexon |
| XLOC_047914 | 0 | 1 | 12 | 0 | - | singleexon |
| XLOC_027492 | 0 | 0 | 2 | 3 | + | singleexon |
| XLOC_005436 | 0 | 0 | 0 | 3 | + | singleexon |
| XLOC_052505 | 0 | 0 | 4 | 0 | - | multiexon |
| XLOC_050667 | 0 | 0 | 0 | 7 | + | singleexon |
| XLOC_013638 | 0 | 0 | 0 | 11 | + | singleexon |
| XLOC_052456 | 1 | 0 | 3 | 0 | - | singleexon |
| XLOC_049370 | 0 | 0 | 14 | 0 | - | singleexon |
| XLOC_030890 | 3 | 0 | 3 | 0 | - | singleexon |
| XLOC_017243 | 0 | 4 | 1 | 0 | + | singleexon |
| XLOC_036570 | 0 | 0 | 0 | 3 | + | singleexon |
| XLOC_003804 | 2 | 4 | 3 | 0 | + | singleexon |
| XLOC_064172 | 0 | 2 | 0 | 1 | + | multiexon |
| XLOC_059186 | 0 | 1 | 0 | 3 | + | singleexon |
| XLOC_010118 | 0 | 0 | 3 | 1 | - | singleexon |
| XLOC_048639 | 0 | 0 | 4 | 0 | - | singleexon |
| XLOC_045866 | 0 | 0 | 10 | 0 | - | singleexon |
| XLOC_049188 | 0 | 0 | 0 | 7 | + | singleexon |
| XLOC_050245 | 0 | 0 | 11 | 0 | - | singleexon |
| XLOC_003899 | 0 | 0 | 0 | 6 | + | singleexon |
| XLOC_011560 | 0 | 0 | 3 | 0 | - | singleexon |
| XLOC_055997 | 3 | 8 | 5 | 2 | + | multiexon |
| XLOC_048697 | 0 | 2 | 0 | 1 | + | singleexon |
| XLOC_017348 | 0 | 0 | 5 | 0 | - | singleexon |
| XLOC_012673 | 0 | 0 | 0 | 3 | + | singleexon |
| XLOC_054542 | 1 | 0 | 3 | 0 | - | singleexon |
| XLOC_028899 | 1 | 2 | 1 | 3 | + | singleexon |
| XLOC_063216 | 0 | 0 | 3 | 1 | - | singleexon |
| XLOC_016553 | 0 | 0 | 0 | 14 | + | singleexon |
| XLOC_061409 | 0 | 0 | 0 | 8 | + | singleexon |
| XLOC_018349 | 0 | 1 | 0 | 3 | + | singleexon |
| XLOC_038697 | 0 | 1 | 23 | 1 | - | singleexon |
| XLOC_034266 | 0 | 0 | 3 | 0 | - | singleexon |
| XLOC_036855 | 0 | 0 | 7 | 0 | - | singleexon |
| XLOC_035258 | 0 | 0 | 0 | 5 | + | singleexon |
| XLOC_058562 | 0 | 51 | 4 | 0 | + | singleexon |
| XLOC_031252 | 0 | 0 | 0 | 3 | + | singleexon |
| XLOC_056159 | 0 | 0 | 2 | 15 | + | singleexon |
| XLOC_061913 | 0 | 0 | 3 | 0 | - | singleexon |
| XLOC_005053 | 1 | 2 | 28 | 0 | - | multiexon |
| XLOC_027532 | 0 | 1 | 7 | 0 | - | multiexon |
| XLOC_056854 | 0 | 0 | 10 | 0 | - | singleexon |
| XLOC_028232 | 0 | 0 | 0 | 3 | + | singleexon |
| XLOC_059600 | 0 | 0 | 2 | 3 | + | singleexon |
| XLOC_010488 | 0 | 0 | 11 | 0 | - | singleexon |
| XLOC_006041 | 0 | 0 | 6 | 0 | - | singleexon |
| XLOC_001022 | 0 | 0 | 0 | 33 | + | multiexon |
| XLOC_050666 | 0 | 0 | 1 | 21 | + | singleexon |
| XLOC_013595 | 0 | 0 | 1 | 3 | + | singleexon |
| XLOC_012003 | 1 | 0 | 76 | 1 | - | singleexon |
| XLOC_032752 | 0 | 2 | 0 | 7 | + | singleexon |

**Table S4.** Primer Table.

| **ISH primers** |  |  |
| --- | --- | --- |
| **Gene** | **Forward** | **Reverse** |
| *Nb-1* | GGGTTCGAATCCTCGTTGGT | TAGGGCATCTCGAGAGGGTT |
| *Csd* | AGACGCGAAGTGTGGTTGAT | AGGCTTAGATCCTTCTCCCGT |
| *Tra2* | GGAATCATCATTTCCGGCTGTTA | GATGACATTGGACTGCGGGA |
| *Sxl* | GAGTGGTGTAGTGGGGTAGC | GACAAGATTCCACAGGCCCA |
|  |  |  |
| **qRT-PCR primers** |  |  |
| **Gene** | **Forward** | **Reverse** |
| *Nb-1* | TCGGCTTTCTGACAAGAAGTTC | TGACCTTAATCGTGTTGTGGAA |
| *Csd* | TGCGTTCAAGAACAAAAG | CACGACTATCTGGGGATC |
| *Tra2* near | TCTCGATCACGCTCTTATTCAC | AGGTCAAAGGGTCAAGTCACA |
| *Tra2* far | TCTCGATCACGCTCTTATTCAC | CGAGCAAGACAAACATCTTCAG |

**Table S5.** Identification of alternative splicing events of sex determination genes (SDGs). Alternative splicing events were classified into two major types, including intron retention (IR) and non-intron retention (NIR). See the attached Excel file.
